# Structure–Activity Relationships and Target Selectivity of Phenylsulfonylamino-Benzanilide Inhibitors Based on S1647 at the SLC10 Carriers ASBT, NTCP, and SOAT

**DOI:** 10.1021/acs.jmedchem.4c01743

**Published:** 2024-10-17

**Authors:** Marie Wannowius, Christopher Neelen, Philipp Lotz, Michael Daude, Anita Neubauer, Bärbel Fühler, Wibke E. Diederich, Joachim Geyer

**Affiliations:** † Institute of Pharmacology and Toxicology, Faculty of Veterinary Medicine, Biomedical Research Center Seltersberg (BFS), Justus Liebig University of Giessen, Schubertstr. 81, Giessen 35392, Germany; ‡ Department of Medicinal Chemistry and Core Facility Medicinal Chemistry, Center for Tumor- and Immune Biology, Philipps University Marburg, Hans-Meerwein-Str. 3, Marburg 35043, Germany

## Abstract

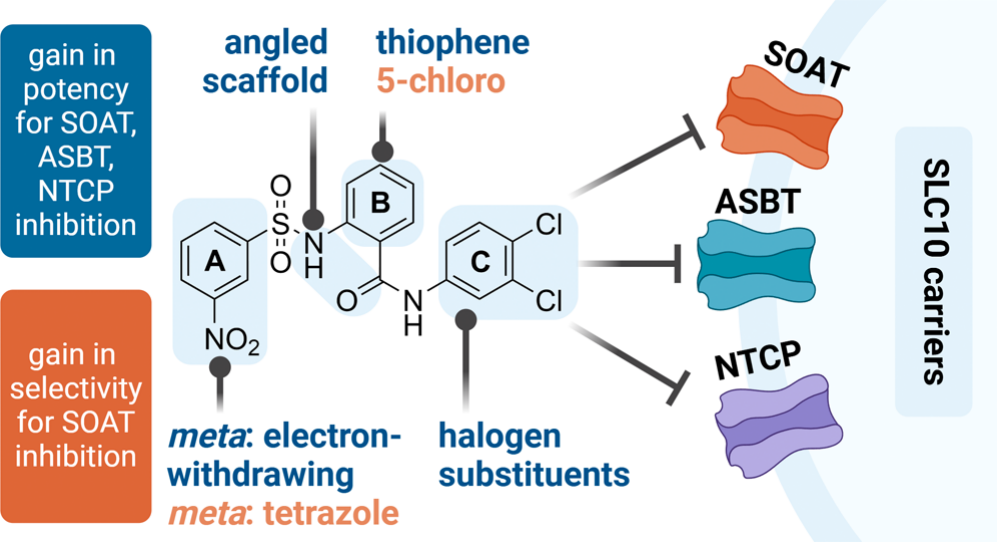

The intestinal bile acid carrier ASBT (*SLC10A2*), the hepatic bile acid carrier NTCP (*SLC10A1*), and the steroid sulfate carrier SOAT (*SLC10A6*), all members of the solute carrier family SLC10, are established drug targets. The ASBT inhibitors odevixibat, maralixibat, and elobixibat are used to treat intrahepatic cholestasis, cholestatic pruritus, and obstipation. The peptide drug bulevirtide blocks binding of the hepatitis B and D viruses to NTCP and thereby inhibits the virus’s entry into hepatocytes. Experimental SOAT inhibitors have antiproliferative effects on hormone-dependent breast cancer cells. The phenylsulfonylamino-benzanilide S1647 is an inhibitor of ASBT and SOAT. The present study aimed to comparatively analyze a set of newly synthesized and commercially available S1647 derivatives for their transport inhibition against ASBT, NTCP, and SOAT. Structure–activity relationships were systematically analyzed regarding potency and target specificity to elucidate whether this compound class is worth being further developed in preclinical studies for pharmacological ASBT, NTCP, and/or SOAT inhibition.

## Introduction

The solute carrier family SLC10 is composed of four orphan transporters (SLC10A3-SLC10A5 and SLC10A7) for which no transporter substrate could be identified yet and three functionally well-characterized carriers, namely, the intestinal bile acid carrier apical sodium-dependent bile acid transporter (ASBT, encoded by *SLC10A2*), the hepatic bile acid carrier Na^+^/taurocholate cotransporting polypeptide (NTCP, encoded by *SLC10A1*), and the steroid sulfate uptake carrier sodium-dependent organic anion transporter (SOAT, encoded by *SLC10A6*).^1−3^

All three carriers have been identified as valuable drug targets. ASBT in the gut is responsible for the reabsorption of bile salt (BS) from the intestinal lumen.^4^ ASBT inhibitors increase fecal BS excretion, lower the hepatic BS load, and thereby protect hepatocytes from elevated toxic BS levels during cholestasis.^5−7^ In addition, ASBT inhibitors lower serum LDL-cholesterol levels by increased *de novo* BS synthesis from cholesterol in the liver.^8^ Clinically, odevixibat, maralixibat, and elobixibat have been approved for the treatment of progressive familial intrahepatic cholestasis, cholestatic pruritus in patients with the Alagille syndrome, and chronic constipation, respectively.

NTCP is the most important uptake carrier for BS in the liver^9^ and more recently has been identified as the high-affinity hepatic entry receptor for the hepatitis B and D viruses (HBV/HDV).^10^ Virus attachment to NTCP occurs via the myristoylated preS1 domain (myr-preS1) of the large virus envelope protein and represents the first essential step of HBV/HDV entry into hepatocytes.^11^ Based on this mechanism, pharmacological inhibition of myr-preS1 peptide binding to NTCP is an attractive new strategy for the development of HBV/HDV antiviral drugs acting as virus entry inhibitors.^12−14^ The first representative of this new drug class, the myr-preS1 analogue bulevirtide, has recently been approved for the treatment of chronic HDV infection. In addition, two orally available NTCP inhibitors successfully passed preclinical development as novel HBV/HDV entry inhibitors, namely, A2342^15,16^ and FRI-231.^17^

SOAT transports sulfated steroid hormones into specific target cells and thereby contributes to the overall regulation of steroid responsive cells and organs.^18−21^ SOAT is highly expressed in the breast cancer tissue, in particular in specimens showing ductal hyperplasia, intraductal papilloma, atypical ductal hyperplasia, intraductal carcinoma, and invasive ductal carcinoma.^22,23^ In breast cancer cells, SOAT-mediated transport of estrone-3-sulfate significantly stimulated *in vitro* cell proliferation, while SOAT inhibitors blocked this effect. Based on these observations, SOAT inhibitors are novel anticancer drug candidates.^22^

**Figure 1:**
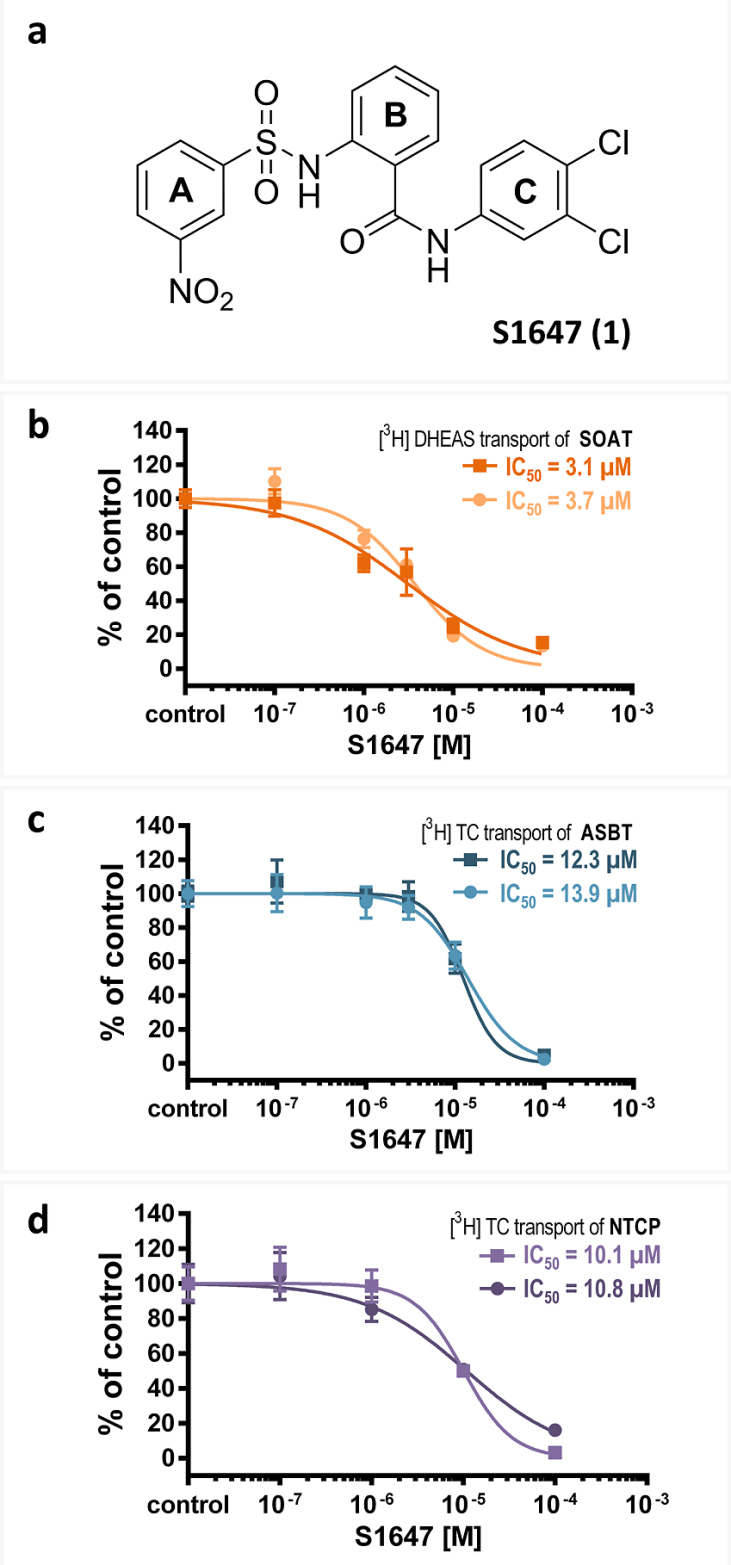
Characterization of S1647 (**1**) as a transport inhibitor (a). Determination of half maximal inhibitory concentration (IC_50_) of S1647 (**1**) against (b) SOAT, (c) ASBT, and (d) NTCP. IC_50_ values are calculated by nonlinear regression analysis and are indicated for two independent experiments each with quadruplicate determinations of each data point [means ± standard deviation (SD)].

Recently, phenylsulfonylamino-benzanilide derivatives showed antihepatic fibrosis activity.^24^ Much earlier, the phenylsulfonylamino-benzanilide compound S1647 (**1**; Figure 1a) has been identified as an inhibitor of ASBT and SOAT,^25,26^ but its interaction with NTCP was unknown so far. The present study aimed to comparatively analyze a set of newly synthesized and commercially available S1647 derivatives for their transport inhibition against ASBT, NTCP, and SOAT. Structure–activity relationships were systematically analyzed regarding potency and target specificity to elucidate if this compound class is worth being further developed in preclinical studies for pharmacological ASBT, NTCP, and/or SOAT inhibition.

## Results and Discussion

The phenylsulfonylamino-benzanilide compound S1647 (**1**; Figure 1a) is composed of three phenyl rings, A, B, and C, whereby the phenyl rings A and B are connected by a sulfonamide group and the phenyl rings B and C are linked by an amide group (Figure 1a). At the B-ring, the sulfonamide and the amide groups are oriented in the *ortho* position. Ring A is additionally substituted with a nitro group in the *meta* position (3-nitro) and the C-ring has two chlorides in *meta* and *para* positions (3,4-dichloro). A previous study already described that regarding potent ASBT inhibition (I) connection of the B-ring is best in the *ortho* position of the sulfonamide and amide bridges [as in S1647 (**1**)], (II) the nitro group of the A-ring in the *meta* position [3-nitro, as in S1647 (**1**)] shows higher activity than the corresponding 4-nitro analogue, (III) the 3-nitro group can be replaced by another electron-withdrawing 3-trifluoromethoxy group, and (IV) substitution of the C-ring is preferred with 3-chloro-4-fluoro or 2,4-dichloro substitutions.^27^ Finally, the best performing ASBT inhibiting compound had a 3-trifluoromethoxy substitution of the A-ring and a 3-chloro-4-fluoro substitution of the C-ring.

### S1647 (**1**) is a Nonselective Multitarget Inhibitor of SOAT, ASBT, and NTCP

In the present study, we first comparatively analyzed the inhibitory potency of S1647 (**1**) on the closely related carriers SOAT, ASBT, and NTCP. As shown in Figure 1b–d, S1647 (**1**) had mean IC_50_ values against SOAT, ASBT, and NTCP of 3.5, 13.4, and 10.4 μM, respectively. These data confirm the previously identified activity of S1647 (**1**) to inhibit ASBT^25^ and SOAT^26^ and shows for the first time comparable inhibition of NTCP, indicating that S1647 (**1**) represents a nonselective SLC10 inhibitor. We therefore chose S1647 as the starting molecule to elucidate whether a certain target selectivity can be achieved by structural modifications. Due to the simplicity of the synthesis, we initially focused only on the variation of the substitution pattern of the three phenyl rings.

### Substitutions at the A-Ring

**Figure 2:**
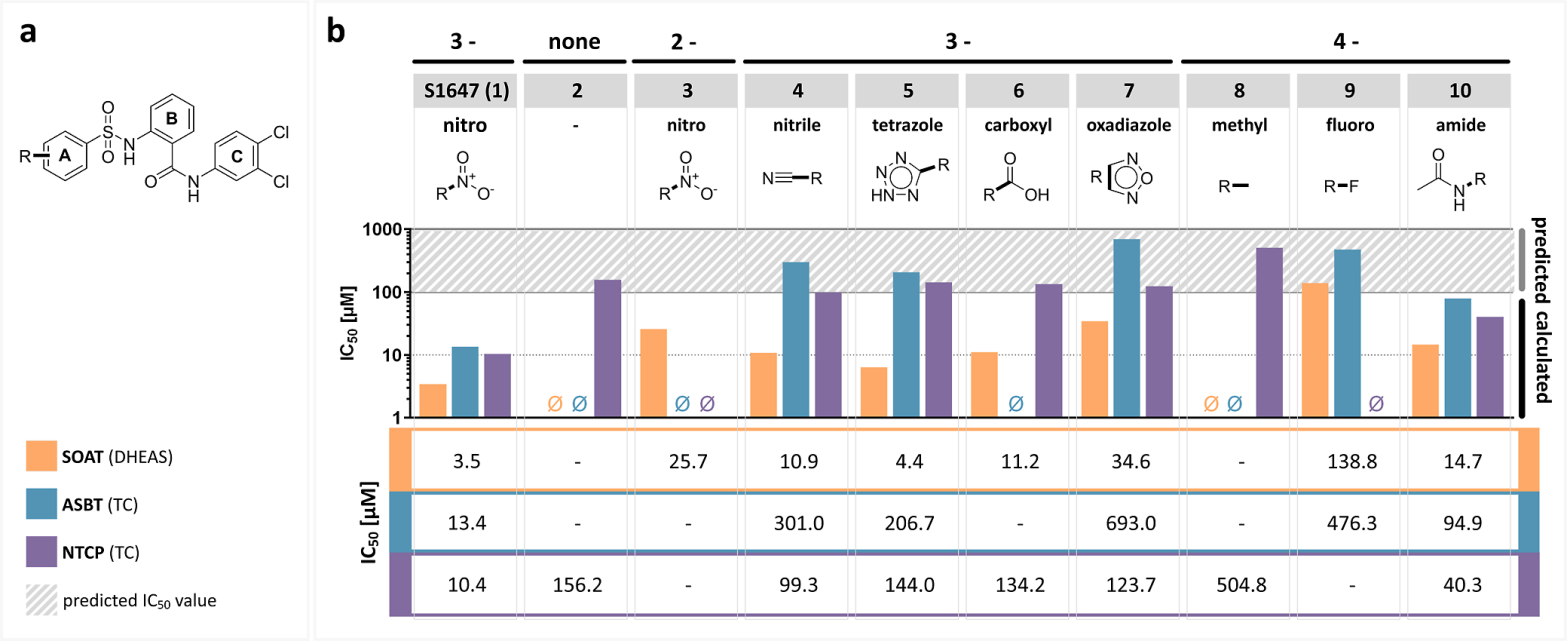
Modifications of the A-ring in *ortho*, *meta*, and *para* positions. (a) Scaffold structure. (b) Substituents R of the A-ring. IC_50_ values of the corresponding compounds were determined by nonlinear regression analysis and are presented as bar graphs in the log scale and numerically as means of two independent experiments each with quadruplicate determinations. The maximum inhibitor concentration was at 100 μM. Accordingly, IC_50_ values >100 μM were extrapolated (predicted). “⌀”, IC_50_ values could not be determined. The full IC_50_-curves and 95% confidence intervals of the IC_50_ values are listed in the Supporting Information section.

First, the A-ring of the S1647 (**1**) molecule was modified to verify the importance and position of the nitro group (**1** with 3-nitro, **2** without the nitro group, and **3** with 2-nitro) and the 3-nitro group was replaced by sterically comparable electron-withdrawing groups, namely, nitrile (**4**), tetrazole (**5**), carboxyl (**6**), and oxadiazole (**7**). In addition, 4-methyl (**8**), 4-fluoro (**9**), and 4-amide (**10**) substitutions were tested in the *para* position of the A-ring. As reported before, a shift of the nitro group to the *para* position significantly reduced the inhibitory potency against ASBT (*meta*: 99.1% inhibition vs. *para*: 66.9% inhibition).^27^ To complete this series, we analyzed the effect of the nitro group in the *ortho* position (**3**) (Figure 2). Here, the inhibitory potency against ASBT as well as against NTCP was completely lost. However, this compound was still active against SOAT, but with a much lower potency (IC_50_ values for the 2- and 3-nitro derivatives of 25.7 and 3.5 μM, respectively).

In contrast, the A-ring without any substitution (**2**), made the compound inactive against SOAT and ASBT, but retained some residual activity against NTCP (IC_50_ = 156.2 μM). Regarding the 3-nitrile (**4**), 3-tetrazole (**5**), 3-carboxyl (**6**), and 3-oxadiazole (**7**) substitutions at the A-ring, none of these were superior to the 3-nitro group of the parent compound S1647 (**1**). However, the 3-tetrazole (**5**) substitution was equipotent against SOAT (IC_50_ = 4.4 μM) compared to the 3-nitro substitution, while losing potency against ASBT (IC_50_ = 206.7 μM) and NTCP (IC_50_ = 144.0 μM), indicating that a certain SOAT selectivity can be achieved at this position. Interestingly, the IC_50_ pattern was SOAT < NTCP < ASBT for all the 3-substituted derivates. Regarding the analyzed substitutions in the *para* position of the A-ring, 4-amide substitution (**10**) uniformly reduced the potency against all three carriers, SOAT (IC_50_ = 14.7 μM), ASBT (IC_50_ = 94.9 μM), and NTCP (IC_50_ = 40.3 μM). The 4-fluoro substitutions (**9**) reduced the potency against all three carriers even more (IC_50_ > 100 μM), and 4-methyl substitution (**8**) made the molecule completely inactive at SOAT and ASBT.

### Halogen Substitutions

**Figure 3:**
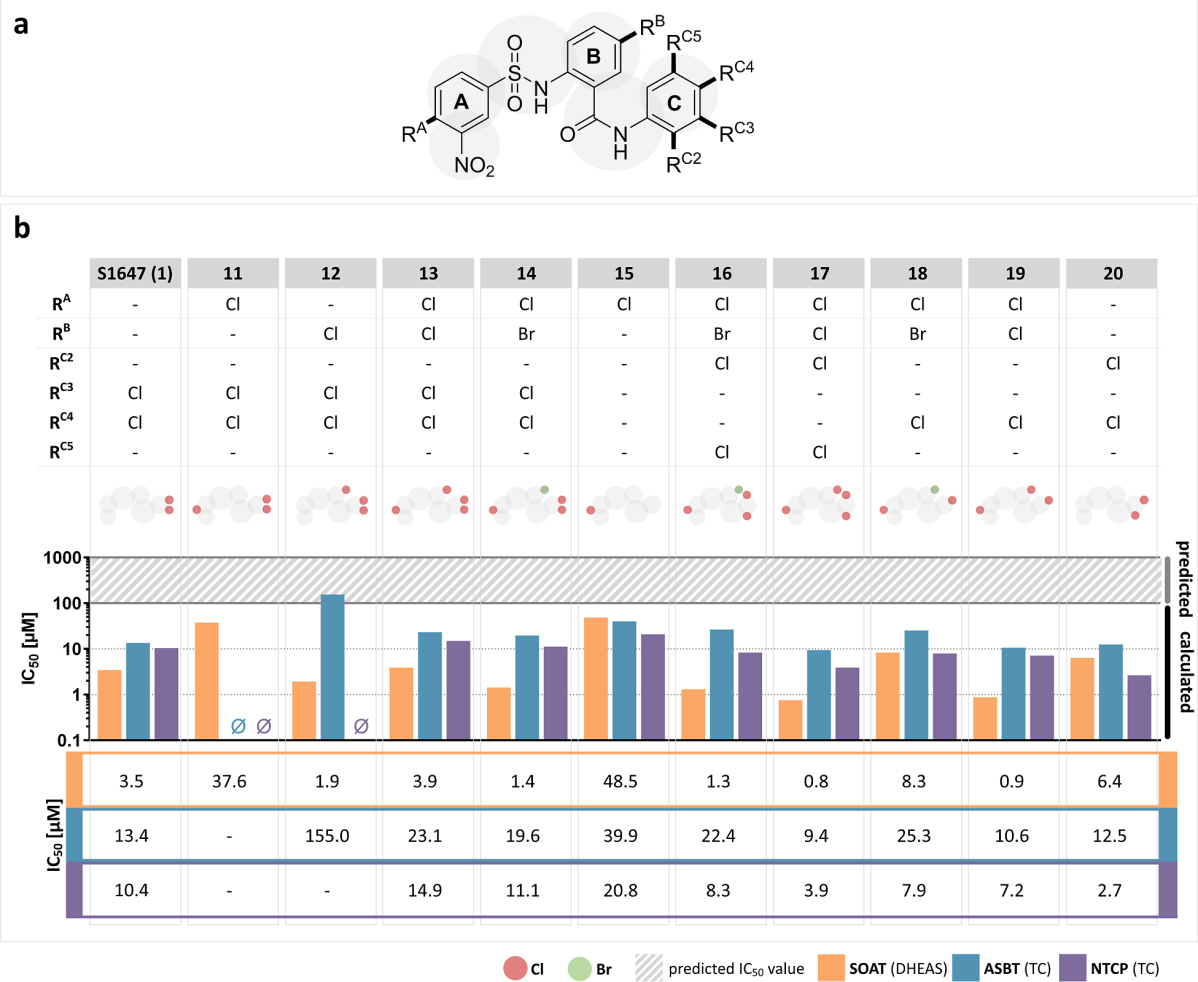
Halogen substitutions at the A-, B-, and C-rings. (a) Scaffold structure. (b) Substituents at A-, B-, and C-rings are indicated. IC_50_ values of the corresponding compounds were determined by nonlinear regression analysis and are presented as bar graphs in the log scale and numerically as means of two independent experiments each with quadruplicate determinations. The maximum inhibitor concentration was at 100 μM. Accordingly, IC_50_ values >100 μM were extrapolated (predicted). “⌀”, IC_50_ values could not be determined or were above the cutoff at 1000 μM. The full IC_50_-curves and 95% confidence intervals of the IC_50_ values are listed in the Supporting Information section.

**Figure 4:**
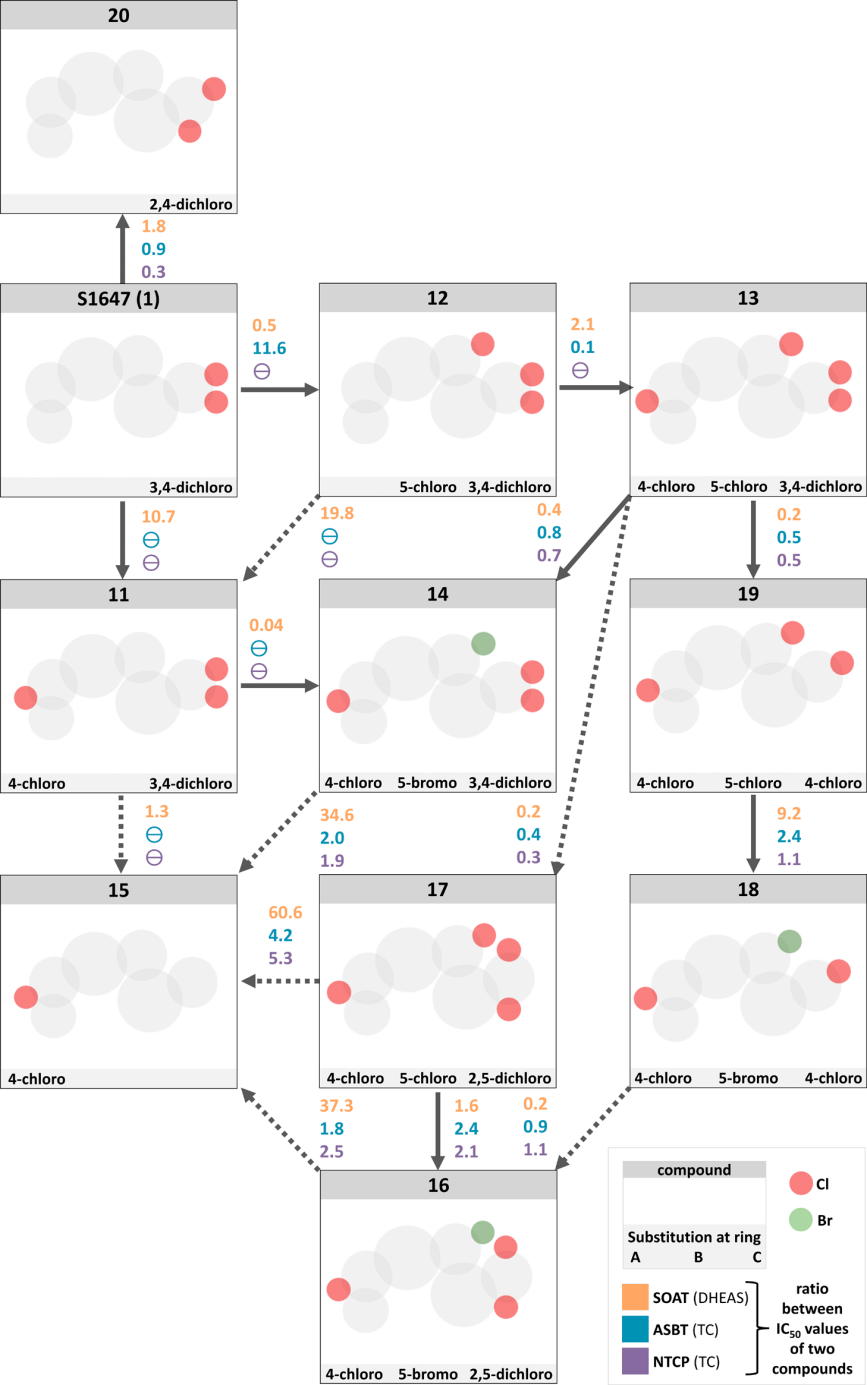
Structure–activity relationships for different halogen substitution patterns of the compounds derived from S1647 (**1**). For each single-position (solid lines) or multiposition (dotted lines) exchange, the effect is indicated as the ratio of the corresponding IC_50_ values at SOAT, ASBT, and NTCP. Values <1 indicate an increase of potency and values >1 indicate decreased potencies in the direction of the arrow. “θ” could not be calculated.

As shown before for ASBT, the number, position, and type of halogen substitutions of S1647 (**1**) had a significant effect on the inhibitory potency.^27^ Therefore, we next tested if the halogen substitution pattern influenced the potency against all three carriers and expanded the halogen substitutions to all three rings. In a first set of compounds, chloro-substitutions were introduced at the *para* positions of the A- and B-rings of S1647 (**1**). As indicated in Figure 3, an additional 4-chloro substitution at the A-ring (**11**) completely abolished the inhibitory effect against ASBT and NTCP and reduced the potency against SOAT to an IC_50_ of 37.6 μM (Figures 3 and 4). In contrast, 5-chloro substitution of the B-ring (**12**) shifted this compound against SOAT selectivity, by retaining the potency against SOAT (IC_50_ = 1.9 μM), significantly reducing the potency against ASBT (IC_50_ = 155.0 μM), and completely abolishing the effect against NTCP. Noteworthy, dichloro substitutions (A-ring: 4-chloro; B-ring: 5-chloro; **13**) restored the inhibition pattern against all three carriers almost to the level of the parent compound S1647 (**1**; Figure 4). For ASBT and NTCP, there was not much difference when the 5-chloro group at the B-ring was replaced by 5-bromo substitution (**14**). However, 5-bromo substitution of the B-ring increased the potency against SOAT to an IC_50_ of 1.4 μM. These data clearly indicate that halogen substitution at the B-ring can be used to increase the selectivity (**12**) and potency (**14** and **12**) toward SOAT. Next, the role of halogen substitutions at the C-ring was analyzed. Interestingly, the ASBT and NTCP loss-of-function compound **11** restored its inhibitory potency by deletion of the 3,4-dichloro substitution of the C-ring (**15**). In contrast, this modification had no effect on SOAT, where **11** and **15** showed comparable IC_50_ values. Therefore, we closer analyzed the halogen substitution pattern of the C-ring. A shift of the 3,4-dichloro substitution of **14** to the 2,5-positions in **16** did not significantly change the activity toward SOAT and ASBT and only slightly increased the potency toward NTCP. In a direct comparison of **16** with **17**, there was a slight increase of the inhibitory potency at all three carriers, indicating that a compound carrying a 2,5-dichloro substitution at the ring C is more potent with a chloro substitution at ring B instead of a bromo substitution at the same position. In contrast, deletion of the 3-chloro substitution of **14** to obtain **18** retained the activity for ASBT and NTCP but reduced the potency toward SOAT to an IC_50_ of 8.3 μM. Even if the chloro-to-bromo exchange of **13** to obtain **14** increased the inhibitory potency against SOAT, chloro-to-bromo exchange of **19** to obtain **18** decreased the potency, indicating that the effect of different halogen substitutions at the B-ring depends on the actual substitution pattern of the C-ring. Finally, in direct comparison of S1647 (**1**) with **20**, the 2,4-dichloro substitution of the C ring is only favorable for NTCP compared to the 3,4-dichloro substitution of S1647 (**1**). Overall, the variation of the halogen substitutions at the C-ring were not appropriate to increase the potency toward ASBT and NTCP or to increase the carrier selectivity. However, bromo-to-chloro exchange at the B-ring (**14** vs. **13**) as well as deletion of 3-chloro substitution (**19** vs. **13**) or a shift of the 3,4-dichloro substitution to the 2,5-positions (**17** vs. **13**) at the C-ring all increased the inhibitory potency toward SOAT. Compounds **19** and **12** are the most attractive molecules from this compound set with high potency at SOAT (IC_50_ = 0.9 μM for **19** and 1.9 μM for **12**) and relatively high target preference toward SOAT compared to ASBT (IC_50_ = 10.6 μM for **19** and 155.0 μM for **12**) and NTCP (IC_50_ = 7.2 μM for **19** and no inhibition for **12**).

### Bridging of the B-Ring

**Figure 5:**
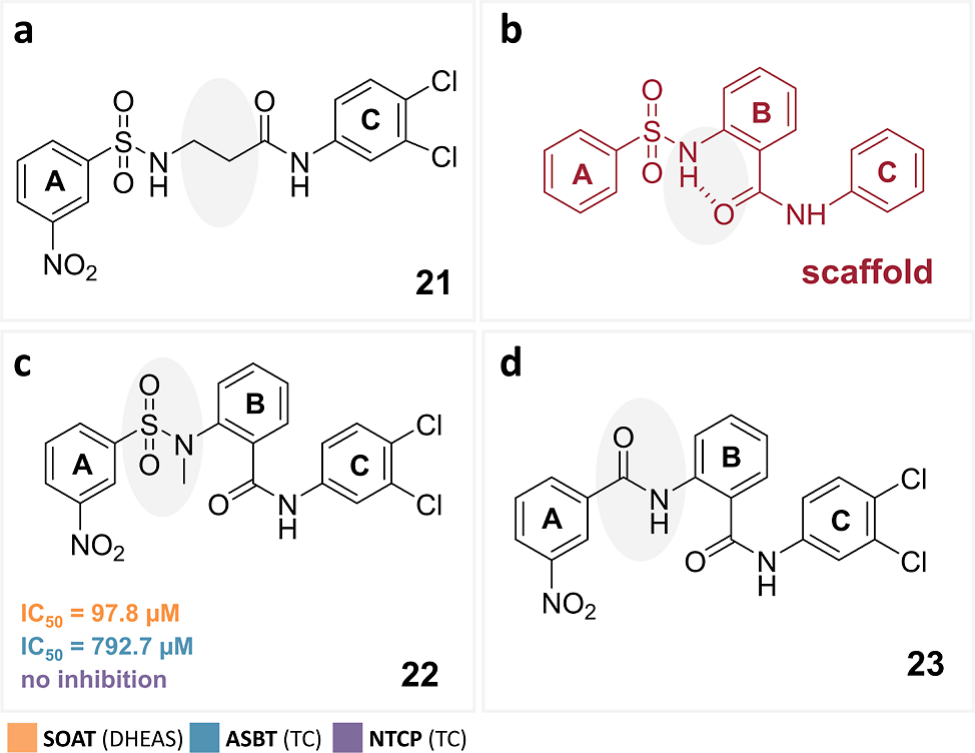
Structures of S1647 (**1**) derivatives with modifications at the B-ring bridge. If the compound was still active as an inhibitor, the corresponding IC_50_ values are indicated. The full IC_50_-curves and 95% confidence intervals of the IC_50_ values are listed in the Supporting Information section.

It was already shown that bridging of the B-ring via the sulfonamide and amide groups is most favorable in the *ortho* position regarding the inhibitory potency against ASBT, while *meta*- or *para*-coupling significantly reduced the activity.^27^ Based on these data, we also analyzed the bridging of the B-ring and replaced the B-ring by a simple aliphatic moiety (**21**, Figure 5a). In addition, it was also evaluated whether a potential intramolecular hydrogen bond (Figure 5b) between the nitrogen of the sulfonamide group and the oxygen of the amide group is relevant for the activity or whether this hydrogen might be involved in an important hydrogen bond to the transporter. In both cases, *N*-methylation should have a clear influence on the affinity (**22**; Figure 5c). Finally, the sulfonyl group connecting ring A with ring B was replaced by an amide group, ending up with two amide bridges (**23**; Figure 5d). Compounds **21** and **23** were entirely inactive. Compound **22** was inactive against NTCP and only retained weak inhibitory potency for SOAT and ASBT with IC_50_ values of 97.8 and 792.7 μM, respectively, clearly indicating that the proposed hydrogen bond plays a significant role for potent SOAT, ASBT, and NTCP inhibition.

### B-Thiophene-Based Inhibitor **24**

**Figure 6:**
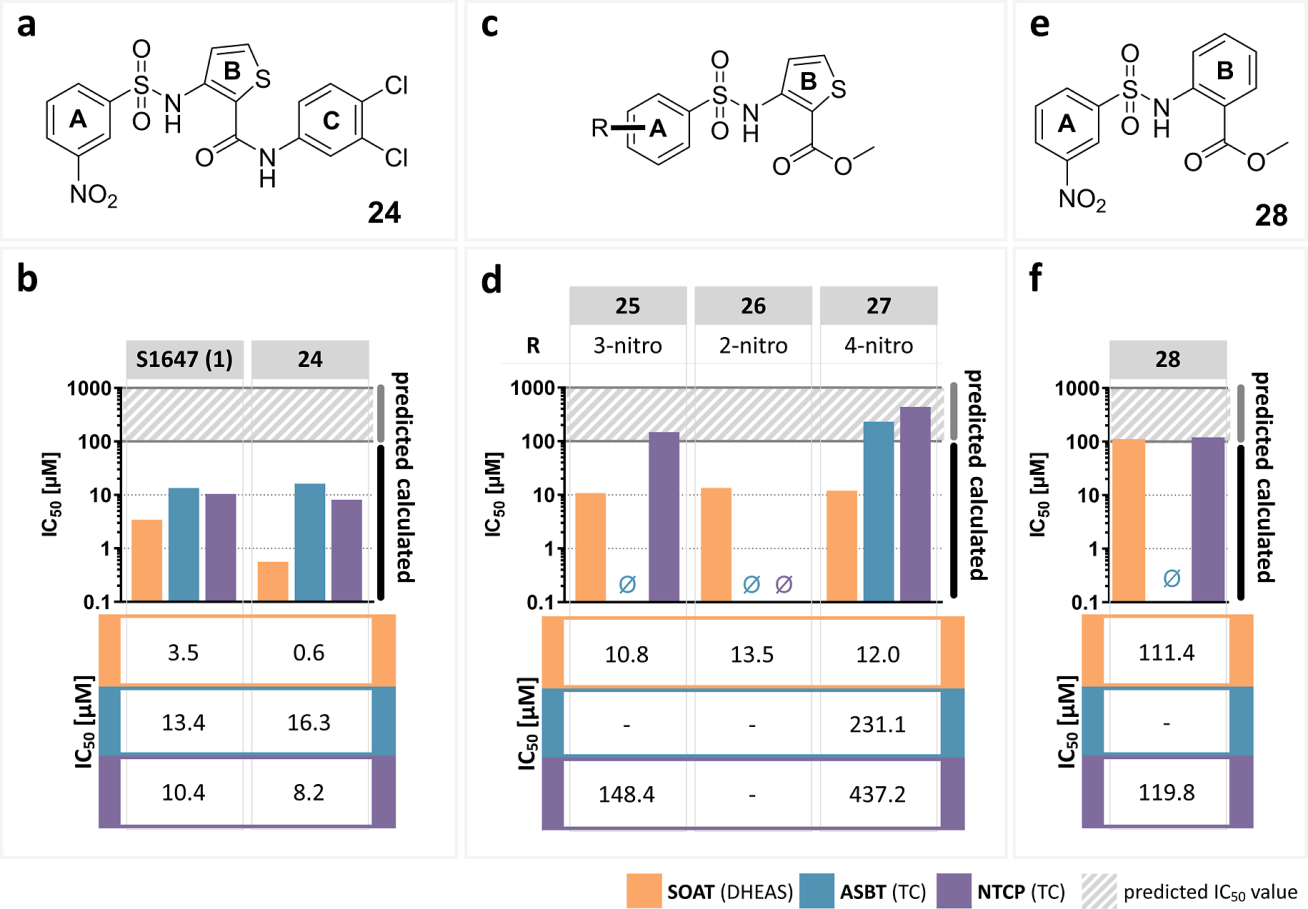
B-thiophene-based inhibitors of SOAT, ASBT, and NTCP. (a) Structure of **24**. (b) Comparison of the IC_50_ values of **24** and S1647 (**1**) for NTCP, ASBT, and SOAT. (c) Scaffold structure of A-phenyl-B-thiophene two ring structures. The nitro group was introduced at different positions on the A-ring. (d) IC_50_ values of the A-phenyl-B-thiophene two-ring-structured compounds. The position of the nitro substitution is indicated in line *R*. (e) Structure of **28**. (f) IC_50_ values of the A-phenyl-B-phenyl compound **28**. IC_50_ values of the corresponding compounds were determined by nonlinear regression analysis and are presented as bar graphs in the log scale and numerically as a means of two independent experiments each with quadruplicate determinations. The maximum inhibitor concentration was at 100 μM. Accordingly, IC_50_ values >100 μM were extrapolated (predicted). “⌀”, IC_50_ values could not be determined. The full IC_50_-curves and 95% confidence intervals of the IC_50_ values are listed in the Supporting Information section.

Next, it was analyzed whether the torsion angle of the *ortho* substitution at the B-ring has an influence on activity. Therefore, the B-phenyl ring was replaced by a planar five-membered ring, namely, thiophene. It was already shown for other receptor ligands that bioisosteric replacement of a phenyl ring by a thiophene ring is well tolerated.^28^ In the present study, the B-thiophene ring significantly increased the inhibitory potency of **24** compared to S1647 (**1**) toward SOAT (IC_50_ = 0.6 μM vs. IC_50_ = 3.5 μM), while there was not much difference for NTCP and ASBT (Figure 6a,b). These data can most likely be explained by the different shape of the molecules with a five-membered B-ring. It can be concluded that the B-thiophene ring significantly increased the inhibitory potency toward SOAT.

### A-Phenyl–B-Thiophene Two Ring Structures

Furthermore, it was elucidated if the full three-ring structure is required for active inhibitors or if the peripheral C-ring can be deleted. For these studies, we used fragments only containing rings A and B connected via a sulfonamide bridge (Figure 6c,d). Interestingly, compound **25**, which comprises only the A and B rings of **24**, was still an active inhibitor of SOAT, however, with a 18-fold lower inhibitory potency (IC_50_ = 10.8 μM), whereas the potency dramatically dropped for NTCP and even more for ASBT. Based on **25**, different positions of the nitro group were tested as before (see Figure 2). Moving the nitro group to the *ortho* (**26**) or *para* position (**27**) did not significantly change the inhibition pattern. Both compounds **26** and **27** demonstrated no or very low activity against ASBT and NTCP but retained comparable inhibitory potency against the SOAT carrier (IC_50_ = 10.8 μM for **25**, IC_50_ = 13.5 μM for **26**, and IC_50_ = 12.0 μM for **27**), indicating that the position of the nitro group has no critical role for SOAT inhibition for the A-phenyl-B-thiophene two ring structures. This contrasts with the three-ring structures, where the position of the nitro group had a significant effect on the inhibitory potency [see Figure 2 and S1647 (**1**) vs. **3**]. Based on these structure–activity relationships, phenylsulfonylamino-thiophene is another promising scaffold structure for further development of potent and selective SOAT inhibitors.

### A-Phenyl–B-Phenyl Two Ring Inhibitor 28

To analyze if the activity of the two-ring structure-based inhibitors depends on the presence of the B-thiophene ring of **25**, the thiophene ring was replaced by a B-phenyl ring in **28** (Figure 6e). As for **25**, this compound was completely inactive against ASBT and showed moderate comparable inhibition of NTCP. However, the potency for SOAT inhibition dropped from an IC_50_ of 10.8 μM (**25** with a B-thiophene ring) down to 111.4 μM (**28** with a B-phenyl ring; Figure 6f), clearly underlining that the B-thiophene ring is much more favorable for potent SOAT inhibition.

### Screening of S1647 (**1**) Derivatives

**Figure 7:**
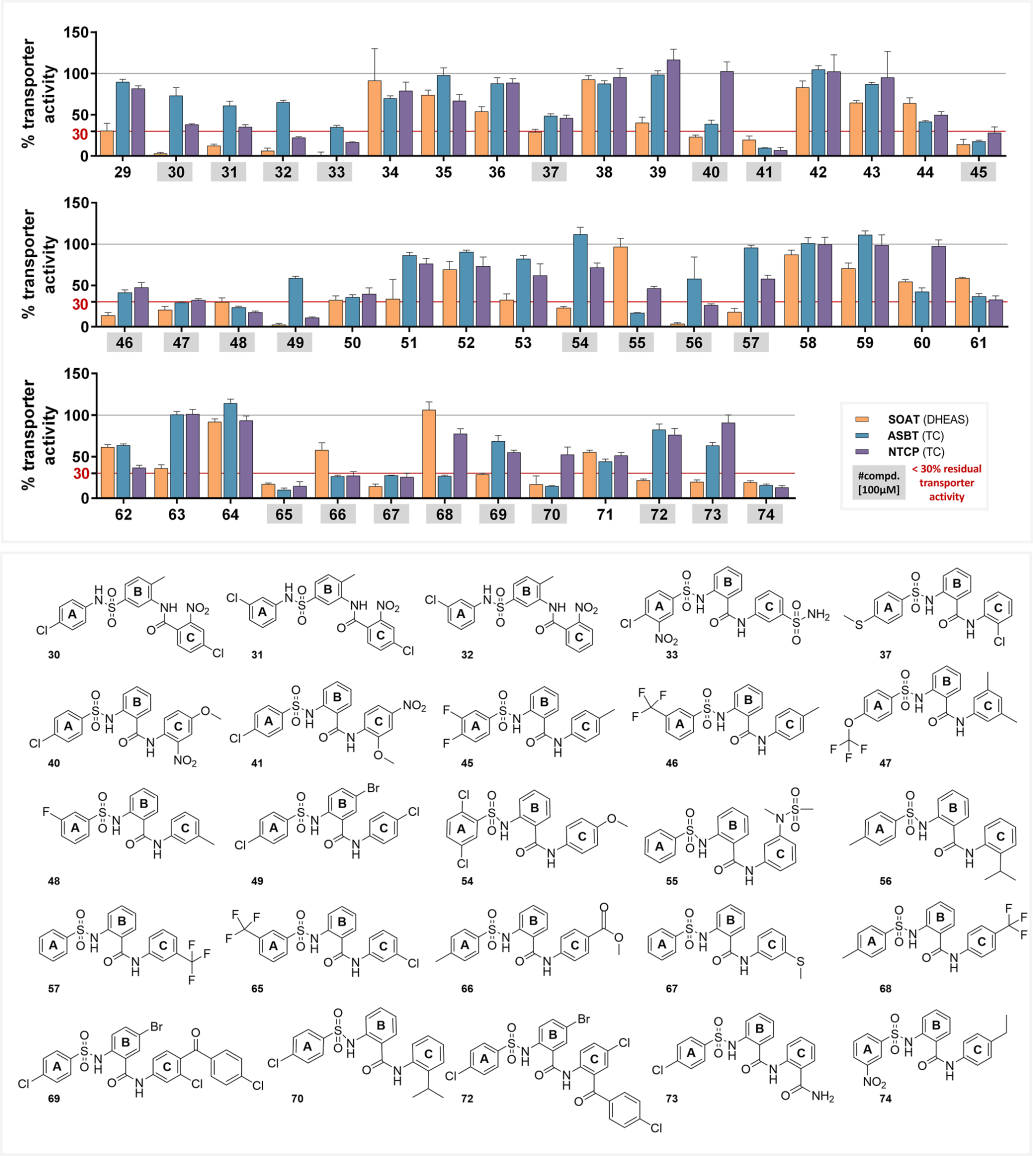
SOAT, ASBT, and NTCP inhibition patterns of S1647 (**1**) derivatives obtained from MolPort. Each compound was tested at 100 μM inhibitor concentration and the residual transport activity (in % of the solvent control) in the presence of inhibitor is indicated. Data represent means ± SD of one screening experiment with quadruplicate determinations. Compounds that inhibit one of the three transporters to a residual activity of less than 30% (red line) are highlighted in gray. The chemical structures of these compounds are presented in the lower panel. These compounds were selected for IC_50_ determinations. The full IC_50_-curves and 95% confidence intervals of the IC_50_ values are listed in the Supporting Information section. The IC_50_ values are listed in Table 1.

Finally, we used the S1647 (**1**) molecule as the scaffold query to search for similar molecules at the MolPort platform. After data curation, we ended up with 46 molecules. All molecules were obtained from MolPort and were in a first approach screened at a 100 μM inhibitor concentration to inhibit DHEAS transport via SOAT as well as BS transport via ASBT and NTCP. Full screening data are shown in Figure 7 and reveal some active inhibitors for all three carriers. The most potent compounds (<30% residual activity at 100 μM inhibitor concentration for at least one carrier) were selected for full IC_50_ determination at all three carriers. All IC_50_ data are summarized in Table 1.

**Table 1:** IC_50_ Determination of S1647 (**1**) Derivatives Obtained from MolPort^a^

compound	SOAT IC_50_ (μM)	ASBT IC_50_ (μM)	NTCP IC_50_ (μM)
30	31.6	no inhibition	113.5
31	10.6	194.5	131.7
32	11.5	130.6	155.3
33	2.4	65.3	17.7
37	5.5	55.9	11.1
40	1.6	no inhibition	14.3
41	4.9	8.6	7.7
45	10.5	16.4	11.4
46	11.5	17.9	8.9
47	19.9	17.1	16.0
48	20.0	34.0	13.8
49	7.1	38.6	17.7
54	no inhibition	15.9	45.6
55	175.7	8.7	88.9
56	2.6	52.0	35.8
57	23.7	no inhibition	332.6
65	6.3	179.2	12.1
66	44.3	62.5	55.7
67	14.0	20.5	28.9
68	no inhibition	39.0	13.1
69	35.9	664.9	40.7
70	15.0	22.9	11.4
72	4.0	374.7	178.2
73	36.6	182.2	95.8
74	16.2	19.32	12.4

a For SOAT, DHEAS was used as the transport substrate; for ASBT and NTCP, TC was used as the transport substrate. The full IC_50_-curves and 95% confidence intervals of the IC_50_ values are listed in the Supporting Information section.

### Potent SOAT, ASBT, and NTCP Inhibitors from the Screening Approach

**Figure 8:**
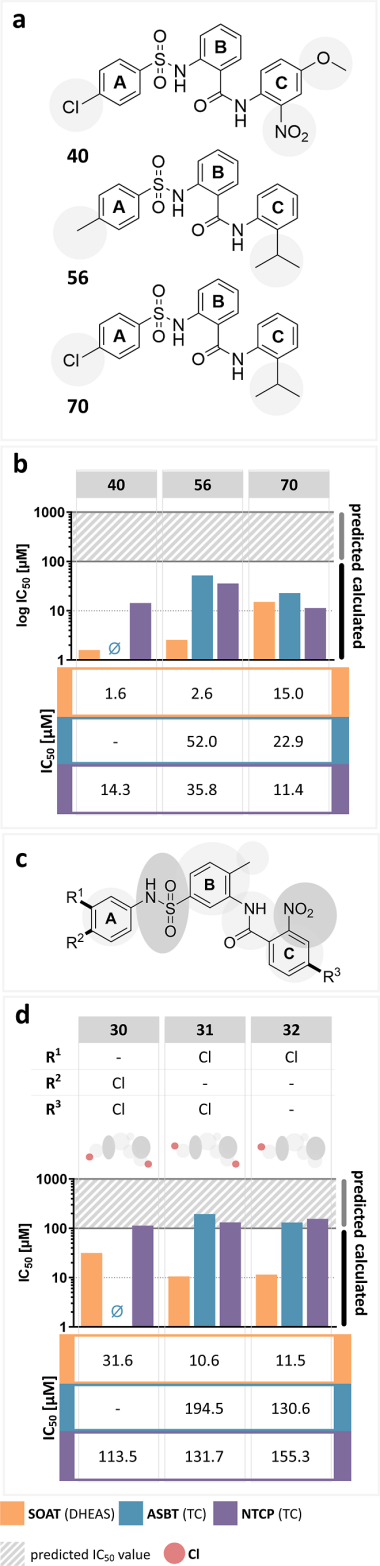
(a) Chemical structures and (b) activities of active SOAT, ASBT, and NTCP inhibitors that were selected from the compound screening set. All compounds slightly differ from the scaffold structure of S1647 (**1**) at the sites marked by gray shading. (c) Chemical structures of compounds **30–32** that have inverted sulfonamide and amide bridges in *meta* position and only differ in their chloro-substitution pattern at the A- and C-rings and (d) their inhibition activities against SOAT, ASBT, and NTCP. IC_50_ values of the corresponding compounds were determined by nonlinear regression analysis and are presented as bar graphs in the log scale and numerically as means of two independent experiments each with quadruplicate determinations. The maximum inhibitor concentration was at 100 μM. Accordingly, IC_50_ values >100 μM were extrapolated (predicted). “⌀”, IC_50_ values could not be determined. The full IC_50_-curves and 95% confidence intervals of the IC_50_ values are listed in the Supporting Information section.

Some of the potent inhibitors share the core structure with S1647 (**1**) with few additional modifications at the A, B, and C rings that have not been systematically analyzed in the present study regarding structure–activity relationships. The structures of all compounds are presented in the Supporting Information, section. In **40**, the nitrogen group is shifted to the C-ring, **56** has an additional methyl group at the A-ring, and **70** has a 2-propyl modification of the C-ring (Figure 8a). Of note, none of these compounds has a nitro-substitution at the A-ring. All three compounds show a quite different inhibition pattern. While **40** is a very potent SOAT inhibitor (IC_50_ = 1.6 μM) and moderate inhibitor against NTCP (IC_50_ = 14.3 μM), it is inactive at ASBT. Compound **56** is active against all three carriers, but in direct comparison to compound S1647 (**1**) is more potent against SOAT (IC_50_ = 2.6 μM), but less potent against ASBT and NTCP, indicating higher target specificity toward SOAT. In contrast, **70** showed moderate comparable inhibition of all three carriers with IC_50_ values ranging from 11.4 μM (for NTCP) to 22.9 μM (for ASBT).

In another subgroup of molecules (**30–32**), the nitro group is shifted from the A-ring, as in compound S1647 (**1**), to the C-ring (Figure 8c). In addition, these molecules have inverted sulfonamide and amide bridges connecting the A–B and B–C rings, respectively, and this bridging is in the *meta* position. These compounds only differ in the substitution pattern with chloride at the A- and C-rings. Compound **30** with A-4-chloro and C-4-chloro substitutions showed a relatively lower potency against SOAT and absent activity against ASBT. **31** with 3-chloro substitution at the A-ring and 4-chloro substitution at the C-ring and **32** with a single 3-chloro substitution of the A-ring both showed comparable IC_50_ values for all three carriers but compared to compound S1647 (**1**) at much higher levels. Overall, these molecules **30–32** are less potent than the S1647-based phenylsulfonylamino-benzanilide derivatives and so are less attractive candidates for SOAT, ASBT, and NTCP inhibition.

### Carrier Selectivity

A particular focus of the present study was to identify structural requirements for the development of target specific SOAT, ASBT, and NTCP inhibitors. Another important question was if potency and selectivity of the inhibitory compounds can structurally be differentiated in a way that target-specific and potent inhibitors can be developed for the carriers of interest.

**Figure 9:**
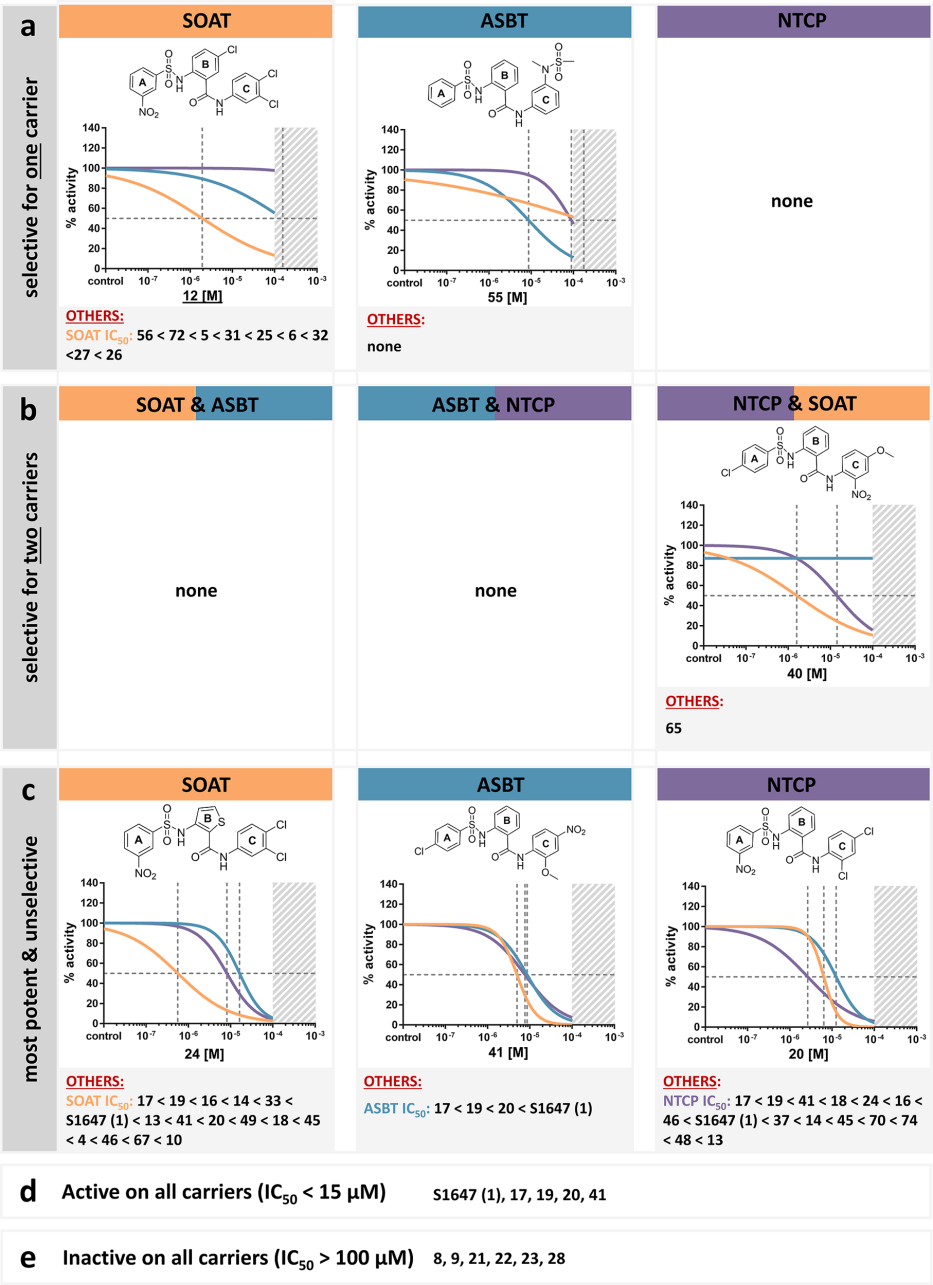
Most selective and potent inhibitors for the respective carriers SOAT, ASBT, and NTCP. (a) Compounds that selectively or dominantly inhibited one particular carrier. (b) Compounds that selectively inhibited two carriers. (c) Nonselective but potent (IC_50_ < 15 μM) compounds. (d) Compounds that were active (IC_50_ < 15 μM) at all three carriers. (e) Compounds that were inactive (IC_50_ > 100 μM) at all three carriers. A compound is described as selective for one carrier, when the IC_50_ value was below 15 μM for the carrier of interest and more than a power of ten higher for the other two carriers. Additionally, the IC_50_ of the other two carriers was required to be above 15 μM. IC_50_ curves and chemical structures are shown for the most potent compounds from each group. Data points and standard deviations are omitted from the IC_50_ curves for better clarity. The other compounds are listed in the descending order of inhibitory potency. Chemical structures, full IC_50_ curves, and 95% confidence intervals of the IC_50_ values are listed in the Supporting Information section for all compounds.

**Figure 10:**
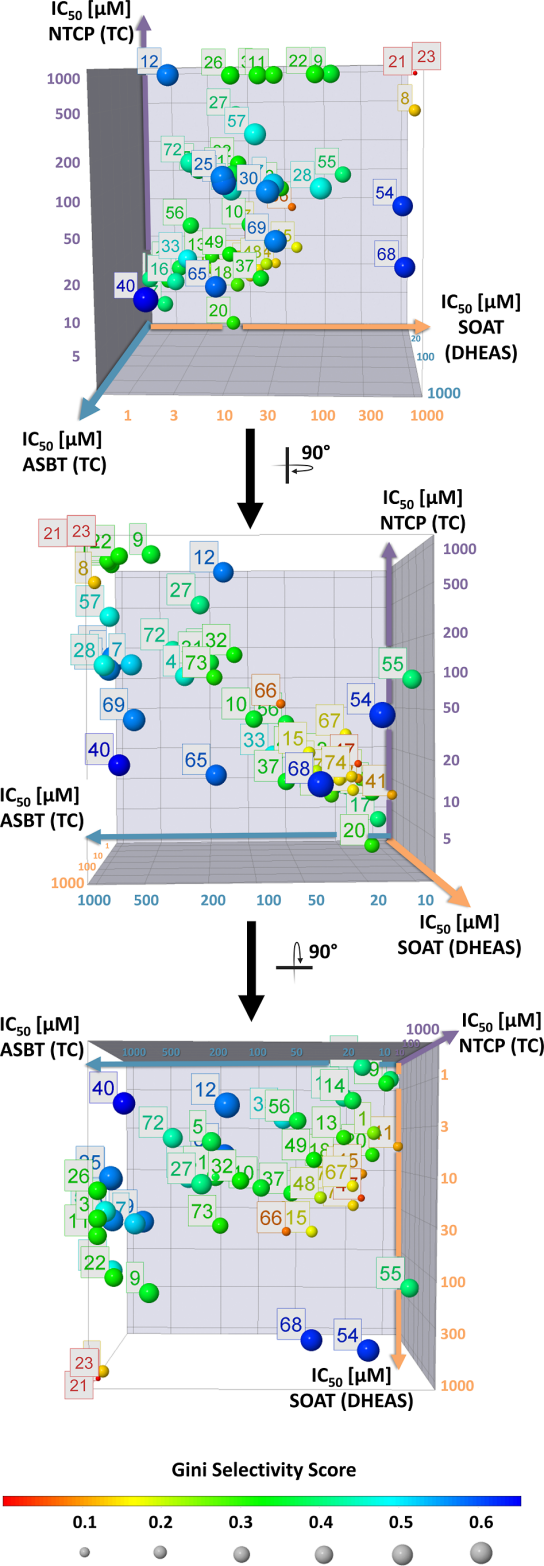
Gini selectivity score. The 3D diagram shows the selectivity score for 53 derivatives as a function of the measured IC_50_ values for the three carriers SOAT, ASBT, and NTCP. The selectivity was calculated based on the Gini selectivity score using the DataWarrior software V6.1.0. It indicates the selectivity of an inhibitor for one carrier in comparison to the other two carriers. Large dark blue dots symbolize a higher selectivity, whereas smaller red dots indicate low selectivity. The closer the dot is to the origin of an axis, the higher the potency of the compound for this carrier.

**Figure 11:**
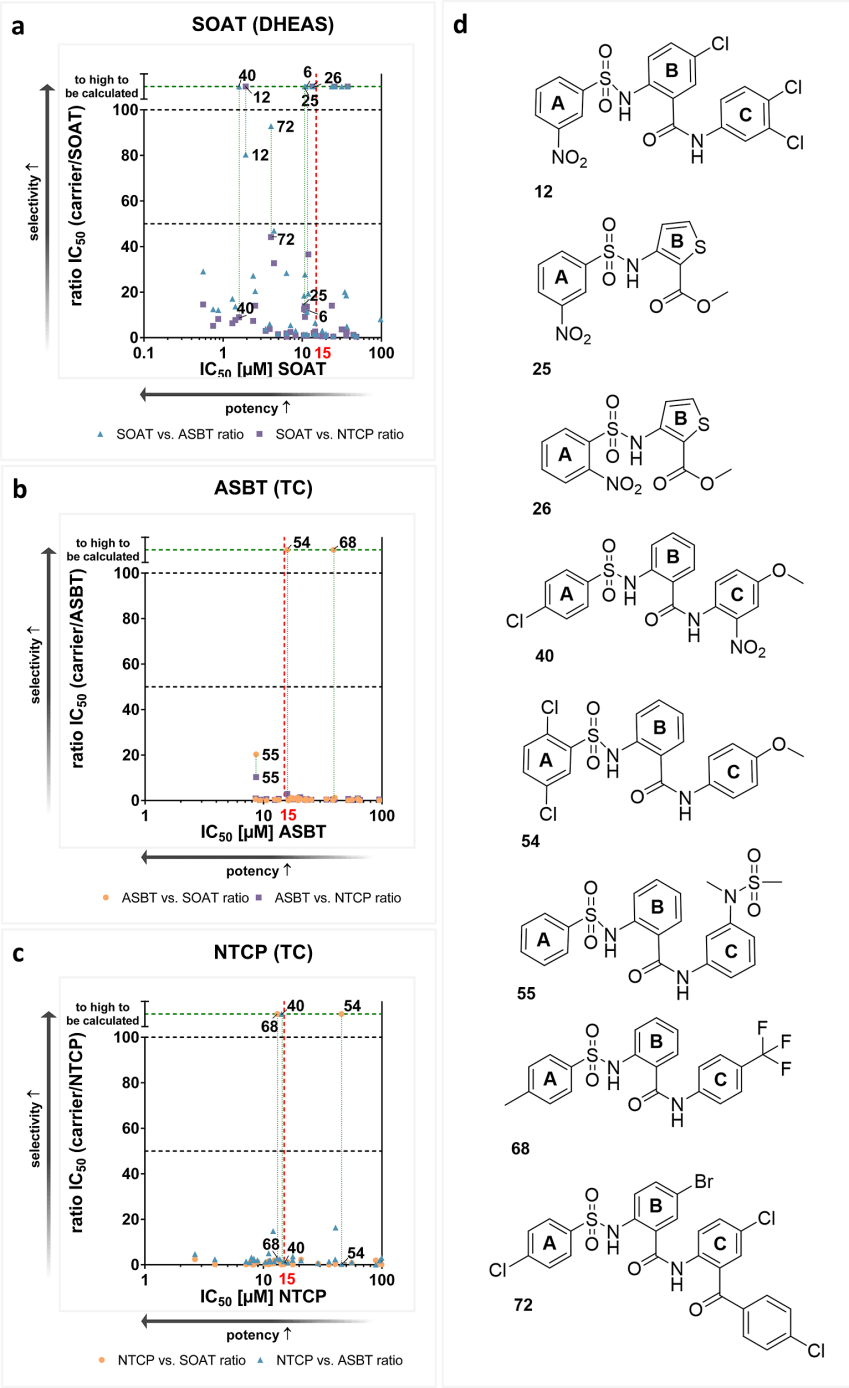
Carrier selectivity and inhibition potencies of the S1647 (**1**) derivatives. (a–c) For each compound, the carrier-specific IC_50_ values (measure of inhibitory potency) were plotted against the IC_50_ ratios of the other two carriers (measure of target specificity). The IC_50_ ratio to ASBT is illustrated with blue triangles, the ratio to NTCP with purple squares and the ratio to the IC_50_ values of SOAT with orange dots. The respective ratios are shown as a function of the IC_50_ value (μM) of the indicated carrier. Compounds for which no ratio could be calculated because no inhibition was measured for one single carrier are shown on a the top of the diagram. (d) Structures of the most selective and potent compounds.

We used basically three different approaches to characterize and analyze the target selectivity and potency of each inhibitor. (I) In the first approach, we defined cutoff values for inhibitory potency based on the IC_50_ values (Figure 9). (II) The second approach used the Gini selectivity score to analyze in parallel selectivity and potency of the compounds (Figure 10). (III) Finally, ratios between the IC_50_ values for the individual carriers were calculated and interpreted for each inhibitor (Figure 11).

#### Cut-Off Values

We defined an inhibitor as meaningful and selective for one particular carrier, when the IC_50_ value was below 15 μM for the carrier of interest and more than a power of ten higher for the other two carriers. Additionally, the IC_50_ of the other two carriers was required to be above 15 μM.

While no compound met these criteria for NTCP, **55** was the only potent and selective inhibitor for ASBT with IC_50_ values of 8.7, 88.9, and 175.7 μM for ASBT, NTCP, and SOAT, respectively (Figure 9a). In contrast, several compounds were potent and selective for SOAT, namely, (with decreasing potency) **12**, **56**, **72**, **5**, **31**, **25**, **6**, **32**, **27**, and **26**. Among them, **12** was most selective with an IC_50_ of 1.9 μM for SOAT, IC_50_ of 155.0 μM for ASBT, and absent potency for NTCP (see Figure 3). Apart from target-specific inhibitors, also dual inhibitors might be of interest (see below). Therefore, the data were also analyzed for dual potent and selective inhibition (Figure 9b). Although no compound fulfilled these strong selectivity criteria neither for SOAT plus ASBT nor for ASBT plus NTCP inhibition, it is noteworthy that two compounds (**54** and **68**) showed at least moderate inhibition and selectivity profiles against ASBT and NTCP with IC_50_ values of 15.9 and 45.6 μM (**54**), as well as 39.0 and 13.1 μM (**68**), respectively, without inhibiting SOAT. These data indicate that dual ASBT/NTCP inhibitors without SOAT cross-reactivity can likely be developed. Furthermore, two compounds showed preferential inhibition of SOAT plus NTCP with low cross-reactivity at ASBT, namely, **40** and **65**. Additionally, all compounds were evaluated for their inhibitory potency against each carrier, without meeting the defined selectivity criteria, but with fulfilling the potency criterion of IC_50_ < 15 μM. In this subanalysis, several representatives were identified for each carrier (see Figure 9c). Ultimately, it is noteworthy that some compounds were able to inhibit all three transporters very well (IC_50_ < 15 μM) and, therefore, have a multitarget mode of inhibition. These were the compounds S1647 (**1**), **17**, **19**, **20**, and **41** (Figure 9d). While **17**, **19**, and **20** are close derivatives of S1647 with modified halogen substitutions at the A-, B-, and C-rings, **41** was identified by compound screening and has significant modifications compared to S1647, namely, the nitro group at the C-ring. In contrast, there were also compounds that were inactive (IC_50_ > 100 μM) at all three carriers, namely, **8**, **9**, **21**, **22**, **23**, and **28** (Figure 9e).

#### Gini Selectivity Score

An additional approach for assessing the selectivity of a compound is the utilization of the Gini selectivity score.^29^ This score was used to evaluate the selectivity of a compound based on its IC_50_ values measured at three different carriers. The range of the score extends from 0 to 1. Values approaching 0 (small, red dots) indicate a low selectivity of the compound (Figure 10). These compounds are either inactive at all carriers, namely, **8** (Figure 2), **21**, and **23** (Figure 5), or are nonselective multitarget inhibitors, namely, (with increasing general activity) **66** < **47** < **45** < **41** (Table 1). In contrast, values in the direction of 1 (large, blue dots) indicate high selectivity. Upon evaluation of the S1647 (**1**) derivative selectivity scores, it becomes evident that most compounds exhibit unselective behavior. In general, an increased potency correlated with the decreased selectivity for most of the compounds. But there are some exceptions from this trend. As an example, **40** that is the compound with the highest Gini selectivity score of 0.655 showed potent SOAT inhibition with IC_50_ = 1.6 μM, much lower inhibitory potency against NTCP (IC_50_ = 14.3 μM), and almost no activity at ASBT. Other compounds with high selectivity scores (greater than 0.5) included **6**, **7**, **12**, **25**, **30**, **54**, **65**, **68**, and **69**. These compounds all fulfill the selectivity criteria defined above (Figure 9), except for **54** and **68** that are only active against ASBT and NTCP.

#### IC_50_ Ratios

The third approach to assess the selectivity and potency of the test compounds involved ratios between the IC_50_ for one of the carriers against the IC_50_ of the other two carriers. These results are presented in Figure 11. As with the approaches I and II, the most potent compounds against NTCP did not exhibit selectivity for this transporter, the ratio of the IC_50_ values is notably low. The same is true for most compounds against ASBT. It is shown again that **54** and **68** are inactive at SOAT but are inhibitors of ASBT and NTCP. In contrast, this analysis yielded a distinct profile for SOAT. The IC_50_ ratios were found to be significantly higher for the high-potency compounds. This was particularly evident for **6**, **12**, **25**, **26**, **40**, and **72**.

Taking the conclusions from I–III together, the most potent inhibitors were generally less selective. This indicates that it is difficult to develop potent and target-selective SOAT inhibitors with low or absent cross-reactivity with ASBT and NTCP. There are only few exceptions, namely, **12**, **40**, and **72** that all showed potent SOAT inhibition with IC_50_ < 10 μM and failed to inhibit at least one of the BS transporters. In contrast to SOAT, no potency/selectivity correlation could be calculated for ASBT or NTCP inhibition. Based on this, further effort is needed to increase in parallel the inhibitory potency and target selectivity for the class of phenylsulfonylamino-benzanilide compounds.

Moreover, follow-up studies should analyze whether S1647 (**1**) and its derivatives may cross-react with other membrane transporters apart from the SLC10 family, e.g., with members of the organic anion transporting polypeptide (OATP) or ATP-binding cassette (ABC) transporter families. This is particularly relevant as the scaffold structure of S1647 (**1**) shows certain similarity to the ABC transporter inhibitor HM30181.^30^ In addition, as S1647 (**1**) shows multitarget inhibition of SOAT, ASBT, and NTCP, it might also be a ligand of the orphan SLC10 members (i.e., SLC10A3-5 and SLC10A7).^1−3^ However, as no substrates and functional transport assays are established for these orphan transporters, it is difficult to analyze this experimentally.

A limitation of the present study is that we could not localize the ligand binding site of S1647 (**1**) and its derivatives. This would have required solid structural information for all three target proteins, namely, SOAT, ASBT, and NTCP. However, structural information is only available for human NTCP from different cryo-EM studies (PDB entries 7PQG, 7PQQ, 7WSI, 7VAG, 7VAD, 7FCI, 7ZYI, 8HRX, 8HRY, and 8RQF). Experimental structural information on SOAT, ASBT, in comparison with NTCP, would also help to optimize target selectivity coupled with optimization of potency by using structure-based drug design approaches.^31^

Among all compounds tested, the highest potencies could be achieved for SOAT inhibition, with three compounds revealing IC_50_ values below 1 μM, namely, **24** (IC_50_ = 0.6 μM), **17** (IC_50_ = 0.8 μM), and **19** (IC_50_ = 0.9 μM). The most potent ASBT inhibitor is **41** with an IC_50_ of 8.6 μM. The most potent NTCP inhibitors with IC_50_ below 5 μM were **20** (IC_50_ = 2.7 μM) and **17** (IC_50_ = 3.9 μM). This indicates that compared to the starting molecule S1647 (**1**) with an IC_50_ of 3.5 μM for SOAT, 13.4 μM for ASBT, and 10.4 μM for NTCP, the inhibitory potencies could be improved 6-fold for SOAT, 1.6-fold for ASBT, and 4-fold for NTCP.

## Chemistry

**Scheme 1:**
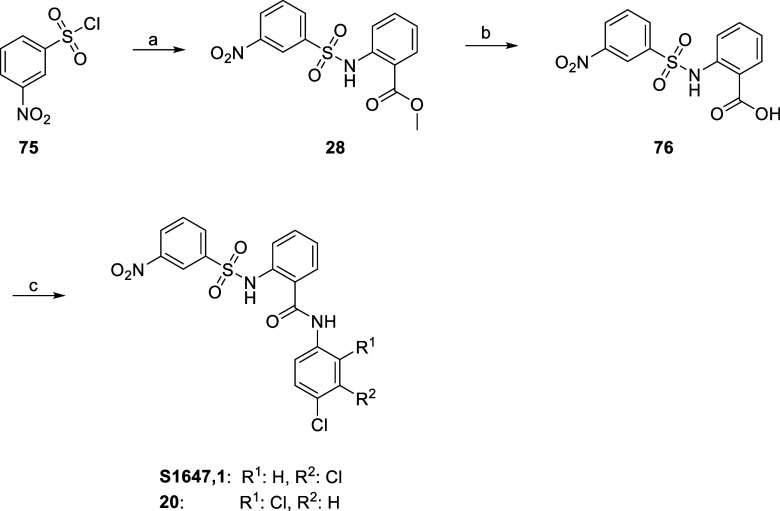
Synthesis of S1647 (**1**) and **20** Reagents and conditions: (a) methyl anthranilate, pyridine, THF, (i) rt, 18 h, (ii) 50 °C, 4 h, 50%; (b) NaOH, EtOH/H_2_O, 85 °C, 16 h, 92%; and (c) for **1**: HOBt, EDC-HCl, 3,4-dichloroaniline, DIPEA, DMF, rt, 72 h, 55%, and for **20**: HOBt, EDC-HCl, 2,4-dichloroaniline, DIPEA, DMF, rt, 72 h, 29%.

**Scheme 2:**
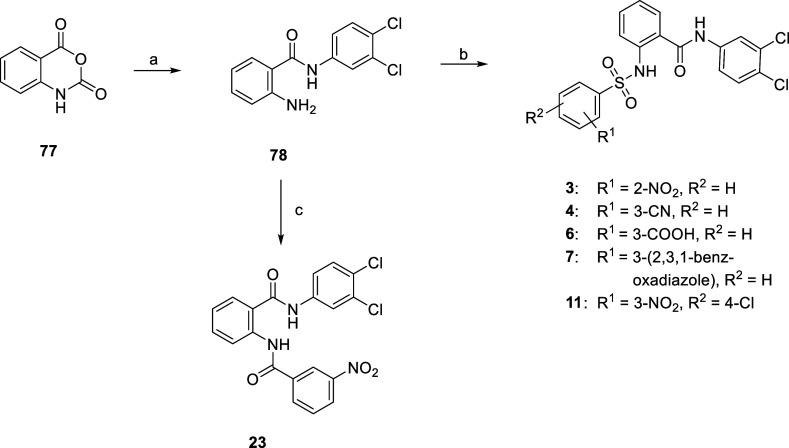
Synthesis of **3**, **4**, **6**, **7**, **11**, and **23** Reagents and conditions: (a) 3,4-dichloroaniline, AcOH, 105 °C, 18 h, 29%; (b) for **3**: 2-nitrobenzenesulfonyl chloride, DCM, pyridine, 0 °C—rt, 5 h, 82%; for **4**: 3-cyanobenzenesulfonyl chloride, DCM, pyridine, 0 °C—rt, 18 h, 87%; for **6**: 3-(chlorosulfonyl)benzoic acid, pyridine, 0 °C—rt, 3 h, 21%; for **7**: 2,3,1-benzoxadiazole-4-sulfonyl chloride, DCM, pyridine, 0 °C—rt, 18 h, 96%; and for **11**: 4-chloro-3-nitrobenzenesulfonyl chloride, pyridine, 0 °C—rt, 3 h, 45%; and (c) (i) 3-nitrobenzoic acid, SOCl_2_, 110 °C, 3.5 h and (ii) TEA, THF, 0 °C—rt, 18 h, 70%.

**Scheme 3:**
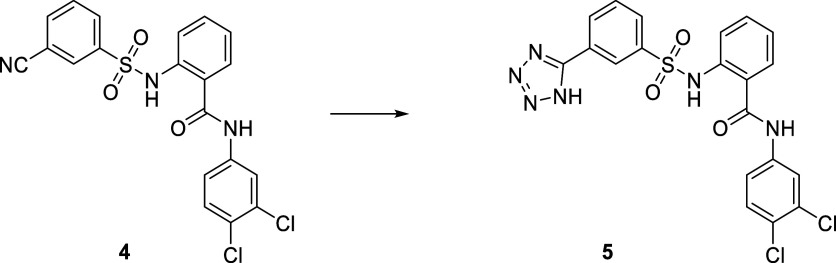
Synthesis of **5** Reagents and conditions: NaN_3_, NH_4_Cl, DMF, 125 °C, 48 h, 63%.

**Scheme 4:**
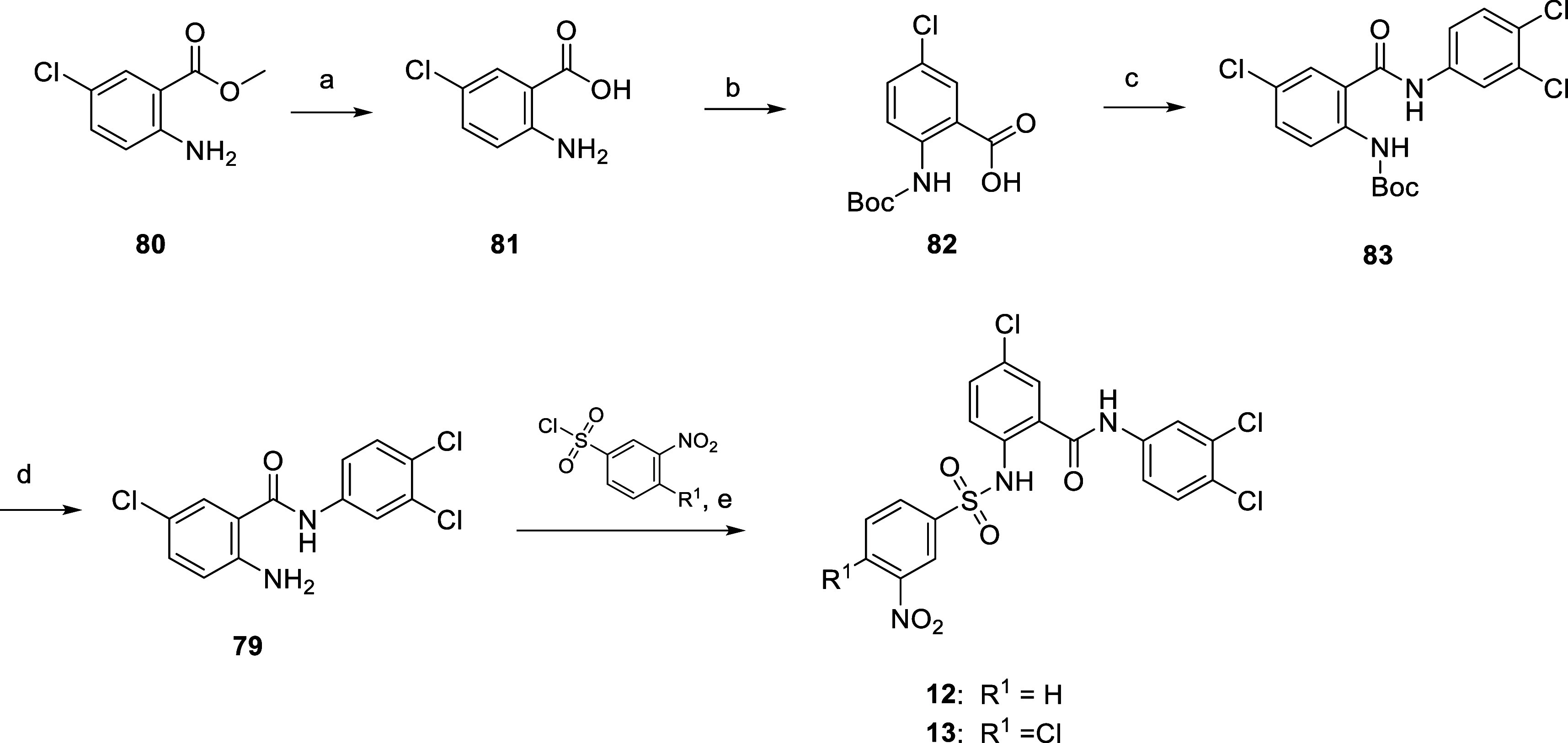
Synthesis of **12** and **13** Reagents and conditions: (a) 2 M NaOH (aq), EtOH, 90 °C, 4 h, 92%; (b) (Boc)_2_O, 2 M NaOH (aq), THF/H_2_O, rt, 40 h, 88%; (c) 3,4-dichloroaniline, HBTU, NMM, dry DMF, rt, 24 h, 66%; (d) TFA, DCM, rt, 3.5 h, 95%; and (e) for 12:3-nitrobenzenesulfonyl chloride, pyridine, 0 °C—rt, 5 h, 43% and for 13:4-chloro-3-nitrobenzenesulfonyl chloride, pyridine, 0 °C—rt, 4.5 h, 46%.

**Scheme 5:**
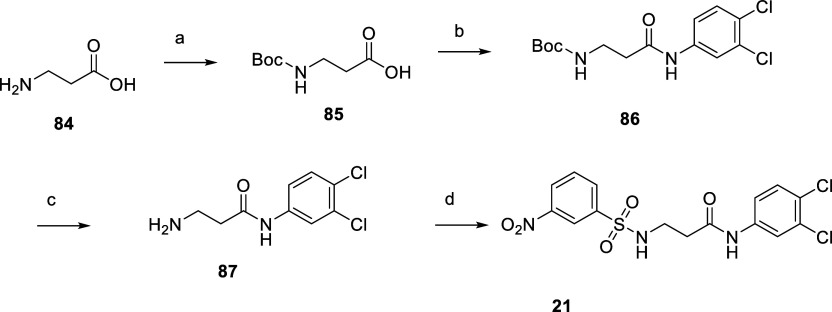
Synthesis of **21** Reagents and conditions: (a) (Boc)_2_O, NaOH, dioxane/H_2_O, rt, 14 h, 63%; (b) 3,4-dichloroaniline, HBTU, NMM, dry DMF, rt, 16 h, 99%; (c) TFA, DCM, rt, 2.5 h, 90%; and (d) 3-nitrobenzenesulfonyl chloride, pyridine, 0 °C—rt, 16 h, 51%.

**Scheme 6:**
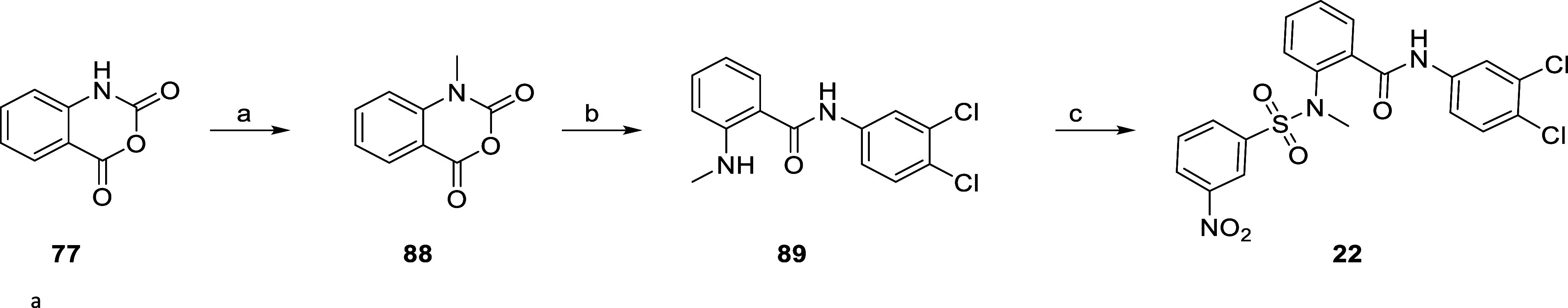
Synthesis of **22** Reagents and conditions: (a) MeI, DIPEA, DMAc, 45 °C, 18 h, 92%; (b) 3,4-dichloroaniline, AcOH, 120 °C, 4 h, 71%; and (c) 3-nitrobenzenesulfonyl chloride, pyridine, 0 °C—rt, 40 h, 47%.

**Scheme 7:**
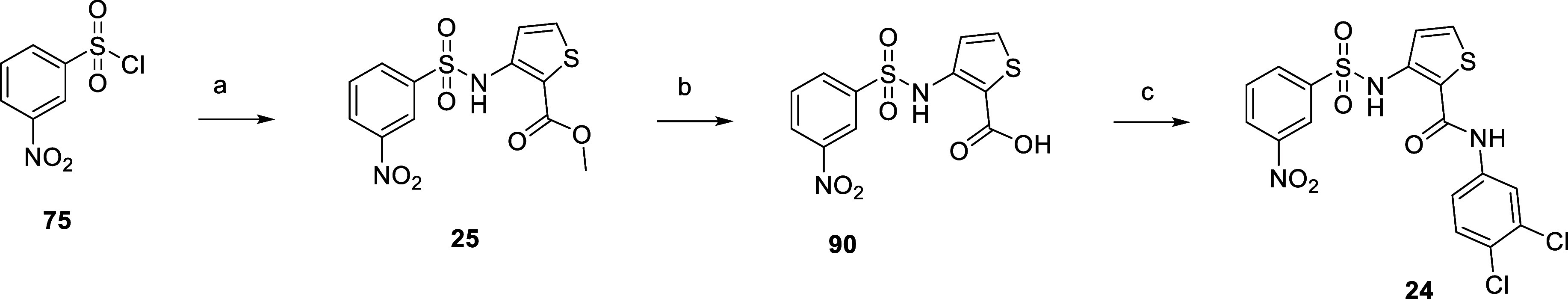
Synthesis of **24** Reagents and conditions: (a) methyl 3-aminothiophene-2-carboxylate, pyridine, THF, 75 °C (24 h)—rt (48 h), 41%; (b) NaOH, EtOH, 80 °C, 18 h, 89%; and (c) (i) HOBT, EDC-HCl, DMF and (ii) 3,4-dichloroaniline, DIPEA, rt (40 h)—50 °C (12 h), 33%.

S1647 (**1**) and its structural isomer (**20**) were synthesized according to a procedure published by Liu et al.,^27^ as outlined in Scheme 1. Briefly, 3-nitrobenzenesulfonyl chloride (**75**) first reacted with methyl anthranilate to yield **28**, and the ester function is subsequently cleaved to afford carboxylic acid **76**. By coupling **76** with 3,4-dichloroaniline, S1647 (**1**) is obtained in a moderate overall yield. It is noteworthy that in the case of **20**, however, the coupling reaction with 2,4-dichloroaniline led to significantly lower yields in our hands. Due to the moderate to low yields in the last step of the above synthesis route and in particular due to the fact that we first wanted to investigate the influence of the substitution pattern of the A-ring in terms of affinity and selectivity in more detail, the published sequence was considered to be insufficient because here the A-ring substituent is introduced in the first step of the synthesis. Therefore, we first reacted 3,4-dichloroaniline with isatoic anhydride **77** to obtain the key intermediate **78**, albeit with a yield of only 29%. Because the coupling of **78** with the corresponding substituted benzenesulfonyl chlorides allowed the synthesis of the inhibitors **3**, **4**, **6**, **7,** and **11** in only one additional step, we nevertheless did not further optimize the first step of this sequence. The coupling reactions mainly proceeded in good yields of 70%–96%. Only in the case of **11** and **6**, lower yields of 45% and 21%, respectively, were encountered (Scheme 2). **23** was prepared from intermediate **78** by reaction with 3-nitrobenzoic acid. **5** was successfully synthesized from **4** utilizing standard reaction conditions, as depicted in Scheme 3. The inhibitors **12** and **13** were synthesized starting from intermediate **79**, which in turn is accessible in four steps, as shown in Scheme 4. Cleavage of the methyl ester function of **80** under basic conditions leads to carboxylic acid **81**, followed by protection of its primary aromatic amino group to carbamate **82**, subsequent coupling with 3,4-dichloroaniline to give **83**, and final cleavage of the BOC protecting group provides central intermediate **79** in a good overall yield of 50%. **21**, in which the B-ring is replaced by an alkyl chain, was synthesized in 4 steps starting from β-alanine (**84**), as shown in Scheme 5. *N*-BOC protection gives **85**, coupling with 3,4-dichloroaniline leads to **86**, cleavage of the BOC protecting group to yield **87** and final reaction with 3-nitrobenzenesulfonyl chloride **75** affords **21** in a good overall yield. **22**, the *N*-methylated derivative of S1647 (**1**), was synthesized starting from isatoic anhydride (**77**), which was first *N*-methylated (**88**). The subsequent ring opening of **88** with 3,4-dichloroaniline proceeds here with significantly better yields than in the synthesis of the corresponding derivative **78** and leads to the methylated intermediate **89**, which is subsequently reacted with 3-nitrobenzenesulfonyl chloride **75** to furnish **22** (Scheme 6). Finally, the B-ring was replaced by a bioisosteric thiophene ring, leading to **24**. Starting from 3-nitrobenzenesulfonyl chloride (**75**), reaction with the commercially available 3-aminothiophene-2-carboxylate leads to **25**. Cleavage of the ester function and subsequent coupling of the resulting carboxylic acid (**90**) with 3,4-dichloroaniline furnished **24** in a three-step synthesis in a good yield, as outlined in Scheme 7.

## Conclusions

There is still a great need to develop oral NTCP, ASBT, and SOAT inhibitors. However, regarding the close phylogenetic relationship, structural homology, and partially overlapping substrate and inhibition patterns of these three transporters, target specificity of the already developed NTCP and ASBT inhibitors as well as the structural basis for the development of carrier selective or pan-SLC inhibitors has not sufficiently been investigated so far. In particular, it is of clinical relevance, if NTCP or ASBT inhibitors might cross-react with SOAT in a multitarget manner and, therefore, could interfere with the regulation of steroid-responsive organs.^18^ Moreover, NTCP/ASBT dual inhibitors might be superior to ASBT-selective or NTCP-selective inhibitors for the treatment of cholestatic diseases because apart from protecting hepatocytes from BS overload they also would prevent BS accumulation in the renal proximal tubule cells and cholemic nephropathy.^32−34^ So, in this case, multitarget inhibition might be favorable. In contrast, SOAT inhibitors should not impair the physiological BS transport via NTCP in the liver and via ASBT in the gut and, therefore, should be target-specific.

In a previous study, the substrate and inhibition patterns of NTCP, ASBT, and SOAT were systematically investigated and revealed some pan-SLC10 inhibitors (e.g., troglitazone, bromosulfophthalein, and erythrosine B), whereas some other compounds were more carrier specific (e.g., betulinic acid, irbesartan, and cyclosporine A for NTCP and SOAT; and ezetimibe for NTCP).^35^

In the present study, structure–activity relationships and SLC10 carrier target specificity were analyzed for a group of phenylsulfonylamino-benzanilide compounds based on S1647 (**1**). In a first strategy, some precise modifications at the S1647 (**1**) core structure were chemically made. In a second approach, a set of commercially available S1647 (**1**) derivatives were screened to randomly identify functionally relevant groups from a larger chemical space. We could define several relevant structure–activity relationships for all three carriers (NTCP, ASBT, and SOAT) that will help to further develop this chemical SLC10 inhibitor class for preclinical and clinical studies.

The key findings of the present study are (I) S1647 (**1**) and other S1647 (**1**) derivatives are also inhibitors of NTCP; (II) electron-withdrawing groups in the *meta* position of the A-ring are important; (III) nitro-substitution of the A-ring is not absolutely essential for potent SOAT inhibition and can also be moved to the C-ring; (IV) selectivity toward SOAT can be achieved by 3-tetrazole substitution of the A-ring and by 5-bromo substitution of the B-ring; (V) halogen substitutions at the C-ring are important for the inhibitory potency, but the exact substitution pattern at the C-ring is not relevant; (VI) a potential intramolecular hydrogen bond between the nitrogen of the sulfonamide group and the oxygen of the amide group is relevant for the function; (VII) more potent SOAT inhibition can be achieved with a B-thiophen ring, suggesting that a different selectivity profile could possibly be achieved by incorporating other heterocycles; and (VIII) the (A)-phenylsulfonylamino–(B)-thiophene two-ring structure is a promising truncated core scaffold structure for multiple derivatizations at least for potent SOAT inhibition. From the whole set S1647 (**1**) derivatives, at least one compound per carrier could be identified with an IC_50_ < 15 μM, in the case of SOAT even with <1 μM. In general, phenylsulfonylamino-benzanilide-based inhibitors seem to be the most potent against SOAT. Regarding carrier selectivity, clear single carrier preference of S1647-based inhibitors could only be identified for SOAT and ASBT but not for NTCP. Dual NTCP/ASBT inhibitors without cross-reactivity toward SOAT seem feasible. In conclusion: the present study provides the basis for further preclinical development of NTCP, ASBT, and SOAT inhibitors of the phenylsulfonylamino-benzanilide class. The next steps will focus on target selectivity and potency among the SLC10 carriers, potential cross-reactivity with other membrane transporters, and DMPK testing.

## Experimental Section

### Chemicals

All chemicals if not otherwise stated were from Sigma-Aldrich (St. Louis, United States). Troglitazone was purchased from Cayman Chemical (Michigan, United States). If indicated the phenylsulfonylamino-benzanilide derivatives were commercially obtained from MolPort (Riga, Latvia). All other phenylsulfonylamino-benzanilide derivatives were chemically synthesized.

### Phenylsulfonylamino-Benzanilide Derivatives

To systematically find commercially available S1647-based phenylsulfonylamino-benzanilide derivatives, the MolPort platform (http://www.molport.com) was screened in October 2022. A similarity search was performed with six different query structures. The query structures I–V represented S1647 (**1**, as a potent SOAT inhibitor),^26^ compound 5g2 (as a potent ASBT inhibitor),^27^ and the S1647 (**1**) derivatives **14**, **19**, and **17** (as potent NTCP inhibitors based on prescreening in the present study). The common scaffold structure of query structures I–V was used as query structure VI. All hits were filtered for a similarity score of the 2D structures of at least 0.7 to a maximum of 0.95 (fingerprint Tanimoto-based 2-dimensional similarity search). This resulted in a total list of 383 hits. To reduce the number of hits, we further filtered the molecules with the clustering tool of the DataWarrior software (openmolecules.org). This tool groups highly similar molecules (based on the descriptor OrgFunctions) into common clusters and differing molecules into separate clusters. A total of 46 clusters were defined and a representative molecule of each cluster was selected for functional analysis and was ordered at MolPort.

### Stably Transfected HEK293 Cell Lines

The full-length open reading frames of human NTCP, ASBT, and SOAT were cloned into the pcDNA5-FRT-TO vector (Invitrogen) based on the cDNA sequences with GenBank accession numbers NM_003049, NM_000452, and NM_197965, respectively. The NTCP and ASBT constructs were C-terminally tagged with the FLAG epitope and the SOAT construct was C-terminally tagged with green fluorescent protein. Sequence-verified clones were stably transfected into Flp-In T-Rex HEK293 cells (Invitrogen) as reported before.^19,35^ From the generated NTCP-HEK293, ASBT-HEK293, and SOAT-HEK293 cells, the transgene expression can be induced by tetracycline treatment. All cell lines were maintained at 37 °C, 5% CO_2_, and 95% humidity in DMEM/F-12 medium (Thermo Fisher Scientific, Waltham, MA, USA) supplemented with 10% fetal calf serum (Sigma-Aldrich, St. Louis, MO, USA), 4 mM l-glutamine (PAA, Cölbe, Germany), and penicillin/streptomycin (PAA). For induction of transgene expression, the medium was supplemented with 1 μg/mL tetracycline (Roth, Karlsruhe, Germany). All cell lines were authenticated by their functional transport properties in the radioactive assay and were free of mycoplasma infections.

### Inhibition Assays and IC_50_ Determination

NTCP-HEK293 and ASBT-HEK293 cells were used for transport experiments with 1 μM [^3^H]TC (20 Ci/mmol, American Radiolabeled Chemicals *via* Biotrend Chemikalien GmbH, Köln, Germany). Transport experiments in SOAT-HEK293 cells were performed with 1 μM [^3^H]DHEAS (88.3 Ci/mmol, PerkinElmer, Waltham, USA). Flp-In T-Rex HEK293 cells were used as a negative control. Cells were seeded onto polylysine-coated 96-well plates, induced with 1 μg tetracycline per mL, and grown to confluence over 72 h at 37 °C. Then, cells were washed with tempered (37 °C) sodium transport buffer (STB, containing 142.9 mM NaCl, 4.7 mM KCl, 1.2 mM MgSO_4_, 1.2 mM KH_2_PO_4_, 1.8 mM CaCl_2_, and 20 mM HEPES, adjusted to pH 7.4) and preincubated with 80 μL STB for 5 min at 37 °C. The medium was replaced by 80 μL STB containing the respective inhibitor or solvent alone (100% uptake control w/o inhibitor), and cells were further incubated for 5 min at 37 °C. After this preincubation, transport experiments were started by adding 20 μL STB containing 5 μM of [^3^H]TC or [^3^H]DHEAS (final substrate concentration: 1 μM). Experiments were stopped after 10 min by washing twice with ice-cold phosphate-buffered saline (PBS, containing 137 mM NaCl, 2.7 mM KCl, 1.5 mM KH_2_PO_4_, 7.3 mM Na_2_HPO_4_, adjusted to pH 7.4), and the plates were kept cool until adding the lysis buffer (1% sodium dodecyl sulfate and 1 N NaOH). Then, the cell-associated radioactivity of [^3^H]TC and [^3^H]DHEAS was quantified by liquid scintillation counting in a Packard Microplate Scintillation Counter TopCount NXT (Packard Instrument Company, Meriden, USA). The inhibitory concentrations were as follows: 0.1, 1, 3, 10, 30, and 100 μM (for prescreen experiments only 100 μM). Troglitazone was used as a pan-SLC control inhibitor at a 100 μM inhibitor concentration. The mean uptake values measured in the Flp-In T-Rex HEK293 control cells were defined as 0% uptake control (control w/o carrier) and were subtracted from all other values. Values from cells without an inhibitor and solvent alone were set to 100% (control w/o inhibitor). Finally, all net transport data were expressed as % of control. All transport or inhibition graphs were generated with GraphPad Prism 6 (GraphPad). Determination of IC_50_ values was done by nonlinear regression analysis using the equation log (inhibitor) vs. response settings. All data points of the IC_50_ curves represent means ± SD of quadruplicate determinations of two independent experiments (*n* = 8). For all active compounds of the screening approach (residual activity of <30%; Figure 7), two independent measurements were performed: prescreening with a 100 μM inhibitory concentration and full IC_50_ measurement with quadruplicate determinations. All the measured IC_50_ curves and 95% confidence intervals are provided in the Supporting Information section. For the inactive compounds (≥30% residual activity), only one screening experiment at 100 μM was carried out.

### Cytotoxicity Assay

A 3-[4,5-Dimethylthiazole-2-yl]-2,5-diphenyltetrazolium bromide (MTT, Sigma-Aldrich) assay was performed to measure the cytotoxicity of the used compounds. Briefly, NTCP-HEK293 cells were incubated with 100 μL from a 100 μM DMEM solution of the respective compound, over 15 min at 37 °C. Afterward, the medium was removed and 100 μL DMEM containing 0.5 mg/mL MTT were added, and cells were incubated for 1 h at 37 °C. Finally, the medium was replaced by 100 μL isopropyl alcohol (Carl ROTH GmbH & CO. KG), and samples were measured by an ELISA reader (GloMax-Multi DetectionSystem, Promega, Madison, WI, USA). See the Supporting Information.

### Purity Statement

The purity of all-synthesized compounds was determined by elemental analysis or qNMR according to the journal’s requirements.

### General Synthetic

Commercially available reagents, chemicals, and solvents were used without further purification, unless otherwise stated. For reactions with moisture- or oxygen-sensitive reagents, the glassware was preheated (450 °C), evacuated, and flushed with argon before use and the respective reactions were carried out under an argon atmosphere. The reaction control was performed by thin-layer chromatography using aluminum-coated TLC plates (MERCK TLC Silica gel 60F254) or by HPLC-MS [1260 Infinity (Agilent), column: Poroshell 120, EC-C18, 2.7 μM, 4.6 mm × 50 mm (Agilent), MS: Expression S CMS (Advion)]. If required for TLC evaluation, KMnO_4_, or Ehrlich’s reagent dipping solutions were used. Column chromatographic purification was performed by MPLC [Reveleris X2 from GRACE (now BÜCHI) and Pure C-850 Flash/Prep from BÜCHI] using prepacked cartridges [FlashPure (Büchi) and PuriFlash (Interchim)] in different sizes (particle size: 15, 30 or 40 μm). Compounds were detected using a UV [254, 265 and 280 nm] or an ELS detector. All the synthesized compounds were dried at 50 °C under high vacuum for several days if necessary. NMR spectra were recorded on a JEOL ECA500, JEOL ECZ400S, or BRUKER AV III HD 300 MHz spectrometer.

Data processing and analysis was performed using Delta software 5.3.1 (JEOL). The chemical shifts are given in parts per million (ppm) and were referenced to the respective residual solvent signal according to the literature.^36^ The following abbreviations were used to describe the signal multiplicities: s (singlet), br s (broad singlet), d (doublet), dd (doublet of doublets), ddd (doublet of doublets of doublets), td (triplet of doublets), t (triplet), dt (doublet of triplets), q (quartet), and m (multiplet). High-resolution mass spectra were recorded using an AccuTOF-GCv (JEOL) or an LTQ-FT system (Thermo Fisher Scientific). A CHN(S) analyzer vario MICRO CUBE (Elementar) was used to determine the elemental analysis. The melting points were determined using an M 5000 device (Krüss) and are uncorrected.

### General Procedure A

In a thoroughly dried N_2_ flask, the respective primary aromatic amine (1.00 equiv) and pyridine (4.00 equiv, previously dried over molecular sieve 4 Å) were dissolved in dry DCM (3.3–4.5 mL/mmol) under an argon atmosphere and, subsequently, the corresponding sulfonyl chloride (1.30 equiv), dissolved in dry DCM (1 mL), was added slowly at 0 °C with vigorous stirring. The reaction mixture was stirred for a further 15 min at 0 °C, then slowly brought to room temperature, and stirred. The reaction progress was monitored by TLC or LC–MS, and reaction times were chosen according to the progress of the reaction.

### General Procedure B

In a thoroughly dried N_2_ flask, the primary aromatic/aliphatic amine (1.00 equiv) was dissolved in pyridine (5 mL, previously dried over molecular sieve 4 Å) under an argon atmosphere and the respective sulfonyl chloride (1.30 equiv) was added in portions at 0 °C or predissolved in pyridine and then slowly added. The reaction mixture was kept at 0 °C for 15 min and then slowly brought to room temperature. The reaction progress was monitored by TLC or LC–MS, and reaction times were chosen according to the progress of the reaction.

### General Procedure C

In a thoroughly dried N_2_ flask, the corresponding benzoic acid derivative (1.00 equiv), HOBt-monohydrate (1.50 equiv) and EDC-HCl (1.50 equiv) were dissolved in dry DMF. The reaction mixture was stirred for 2 h at room temperature under an argon atmosphere, then the corresponding aniline derivative (2.00 equiv) and DIPEA (2.00 equiv) were added, and the reaction mixture was stirred. The reaction progress was monitored by TLC or LC–MS, and reaction times were chosen according to the progress of the reaction.

#### *N*-(3,4-Dichlorophenyl)-2-[(3-nitrophenyl)sulfonamido]benzamide (**1**)

**1** was prepared according to general procedure C reacting **76** (400 mg, 1.24 mmol, 1.00 equiv) with HOBt monohydrate (285 mg, 1.86 mmol, 1.50 equiv), EDC-HCl (289 mg, 1.86 mmol, 1.50 equiv), 3,4-dichloroaniline (402 mg, 2.48 mmol, 2.00 equiv), and DIPEA (320 mg, 2.48 mmol, 2.00 equiv, 0.42 mL) in dry DMF (10 mL). The reaction mixture was stirred for 72 h at room temperature. Then, 1 M HCl (aq 10 mL) was added, and the mixture was extracted with DCM (3 × 20 mL). The combined organic layers were successively washed with a 2 M K_2_CO_3_ solution (aq 20 mL) and a 5% LiCl solution (aq 5 × 10 mL), dried over MgSO_4_, and filtered, and the solvent was removed under reduced pressure. The crude product was purified by column chromatography (cyclohexane/EtOAc 100:0 → 50:50 over 25 min) to give **1** (318 mg, 0.68 mmol, 55%) as a white solid. mp 186.7 °C; ^1^H NMR (500 MHz, DMSO-*d*_6_, 300 K): δ (ppm) 10.41 (s. 1H), 10.35 (s. 1H), 8.39 (dd, ^4^*J* = 2.0 Hz, 1H), 8.33 (ddd, ^3^*J* = 8.3 Hz, ^4^*J* = 2.3 Hz, ^4^*J* = 0.9 Hz, 1H), 8.07 (ddd, ^3^*J* = 8.0 Hz, ^4^*J* = 1.7 Hz, ^4^*J* = 1.2 Hz, 1H), 7.95 (d, ^4^*J* = 2.3 Hz, 1H), 7.74 (dd, ^3^*J* = 8.0 Hz, 1H), 7.63 (dd, ^3^*J* = 8.2 Hz, ^4^*J* = 1.3 Hz, 1H), 7.58 (d, ^3^*J* = 8.9 Hz, 1H), 7.54–7.49 (m, 2H), 7.38–7.36 (m, 2H); ^13^C NMR (125 MHz, DMSO-*d*_6_, 300 K): δ (ppm) 165.0, 147.6, 140.9, 138.7, 134.5, 132.7, 131.9, 131.2, 130.8, 130.4, 129.0, 129.0, 127.4, 125.9, 125.3, 125.1, 121.5, 121.2, 120.0; HR-MS: calcd for C_19_H_13_Cl_2_N_3_O_5_SNa [M + Na]^+^, 487.9845; found, 487.9830; Elemental analysis calcd (%) for C_19_H_13_Cl_2_N_3_O_5_S: C, 48.94; H, 2.81; N, 9.01; S, 6.88. Found: C, 48.90; H, 2.76; N, 8.96; S, 6.24.

#### *N*-(3,4-Dichlorophenyl)-2-[(2-nitrophenyl)sulfonamido]benzamide (**3**)

*N*-(3,4-Dichlorophenyl)-2-[(2-nitrophenyl)sulfonamido]-benzamide was synthesized according to general procedure A, using **78** (250 mg, 0.89 mmol, 1.00 equiv) and 2-nitrobenzenesulfonyl chloride (257 mg, 1.16 mmol, 1.30 equiv). After 5 h reaction time, a red solid had formed, so that the reaction was terminated. After addition of DCM (50 mL), the organic layer was washed with 1 M HCl (aq 2 × 25 mL), dried over MgSO_4_, filtered, and concentrated under reduced pressure. The crude product was purified by column chromatography (cyclohexane/EtOAc 100:0 → 0:100 over 45 min) furnishing **3** (341 mg, 0.73 mmol, 82%) as a red solid. mp 213.5 °C; ^1^H NMR (500 MHz, DMSO-*d*_6_, 300 K): δ (ppm) 10.65 (s, 1H), 10.62 (s, 1H), 8.06 (dd, ^3^*J* = 7.7 Hz, ^4^*J* = 1.4 Hz, 1H), 8.02 (dd, ^4^*J* = 1.2 Hz, 1H), 7.96 (dd, ^3^*J* = 7.7 Hz, ^4^*J* = 0.9 Hz, 1H), 7.83 (td,, ^3^*J* = 7.7 Hz, ^4^*J* = 1.4 Hz, 1H), 7.79 (m, 2H), 7.65–7.60 (m, 2H), 7.55 (dd, ^3^*J* = 7.2 Hz, 1H), 7.50 (dd, ^3^*J* = 7.7 Hz, 1H), 7.30 (br s, 1H); ^13^C NMR (125 MHz, DMSO-*d*_6_, 300 K): δ (ppm) 166.4, 147.4, 138.5, 135.5, 135.0, 132.8, 132.5, 131.1, 130.8, 130.5, 130.5, 129.2, 125.7, 125.3, 125.0, 124.8, 121.8, 121.6, 120.5; HR-MS: calcd for C_19_H_13_Cl_2_N_3_O_5_SH [M + H]^+^, 466.0026; found, 466.0017; Elemental analysis calcd (%) for C_19_H_13_Cl_2_N_3_O_5_S: C, 48.94; H, 2.81; N, 9.01; S, 6.88. Found: C, 49.00; H, 2.92; N, 9.04; S, 6.83.

#### 2-((3-Cyanophenyl)sulfonamido)-*N*-(3,4-dichlorophenyl)benzamide (**4**)

**4** was synthesized according to general procedure A, using **78** (350 mg, 1.24 mmol, 1.00 equiv) and 3-cyanobenzenesulfonyl chloride (327 mg, 1.62 mmol, 1.30 equiv). After 18 h, the reaction mixture was taken up in DCM (50 mL), successively washed with 1 M HCl (aq 25 mL) and a saturated NaCl solution (aq 25 mL), and the organic layer was dried over MgSO_4_, filtered, and concentrated under reduced pressure. After purification by column chromatography (cyclohexane/EtOAc 100:0 → 15:85 over 35 min), **4** (481 mg, 1.08 mmol, 87%) was obtained as a beige solid. mp 196.4 °C; ^1^H NMR (500 MHz, DMSO-*d*_6_, 300 K): δ (ppm) 10.48 (s, 1H), 10.36 (s, 1H), 8.14 (dd, ^4^*J* = 1.7 Hz, 1H), 8.02–7.98 (m, 3H), 7.69–7.64 (m, 2H), 7.61 (d, ^3^*J* = 8.6 Hz, 1H), 7.57 (dd, ^3^*J* = 8.9 Hz, ^4^*J* = 2.3 Hz, 1H), 7.51 (dd, ^3^*J* = 8.4 Hz, ^4^*J* = 1.4 Hz, 1H), 7.33–7.30 (m, 2H); ^13^C NMR (125 MHz, DMSO-*d*_6_, 300 K): δ (ppm) 166.1, 140.5, 138.6, 136.5, 132.1, 131.1, 130.8, 130.6, 130.5, 130.3, 129.0, 127.7, 125.5, 125.4, 123.9, 121.5, 121.5, 120.3, 117.1, 112.4; HR-MS: calcd for C_20_H_13_Cl_2_N_3_O_3_SH [M + H]^+^, 446.0127; found, 446.0127; Elemental analysis calcd (%) for C_20_H_13_Cl_2_N_3_O_3_S: C, 53.82; H, 2.94; N, 9.42; S, 7.18. Found: C, 53.93; H, 2.97; N, 9.35; S, 7.36.

#### 2-{[3-(1*H*-Tetrazol-5-yl)phenyl]sulfonamido}-*N*-(3,4-dichlorophenyl)benzamide (**5**)

**4** (240 mg, 0.54 mmol, 1.00 equiv), NaN_3_ (88 mg, 1.35 mmol, 2.50 equiv) and NH_4_Cl (72 mg, 1.35 mmol, 2.50 equiv) were dissolved in DMF (2.5 mL) and the mixture heated to 125 °C with stirring. After 24 h, NaN_3_ (88 mg, 1.35 mmol, 2.50 equiv) and NH_4_Cl (72 mg, 1.35 mmol, 2.50 equiv) were added, and the reaction mixture was stirred for additional 24 h at 125 °C. After cooling to room temperature, H_2_O (20 mL) was added, and the mixture was extracted with EtOAc (3 × 50 mL). The combined organic layers were successively washed with a 5% LiCl solution (aq 5 × 20 mL) and a saturated NaCl solution (aq 25 mL), dried over MgSO_4_, and filtered. After removal of the solvent under reduced pressure, the crude product was purified by column chromatography [cyclohexane/EtOAc 80:20 → 20:80 over 25 min, addition of formic acid (approximately 0.5%) to both solvents], and **5** (167 mg, 0.34 mmol, 63%) was obtained as a white solid. mp 243.3 °C; ^1^H NMR (500 MHz, DMSO-*d*_6_, 300 K): δ (ppm) 10.43 (s, 1H), 10.36 (br s, 1H), 8.43 (dd, ^4^*J* = 1.7 Hz, 1H), 8.19 (ddd, ^3^*J* = 7.7 Hz, ^4^*J* = 1.7 Hz, ^4^*J* = 1.2 Hz, 1H), 7.93 (dd, ^4^*J* = 1.4 Hz, ^5^*J* = 1.2 Hz, 1H), 7.82 (ddd, ^3^*J* = 8.0 Hz, ^4^*J* = 1.7 Hz, ^4^*J* = 1.2 Hz, 1H), 7.68 (dd, ^3^*J* = 7.7 Hz, 1H), 7.65 (dd, ^3^*J* = 7.7 Hz, ^4^*J* = 1.4 Hz, 1H), 7.53–7.47 (m, 3H), 7.40 (dd, ^3^*J* = 8.3 Hz, ^4^*J* = 1.2 Hz, 1H), 7.30 (ddd, ^3^*J* = 7.5 Hz, ^4^*J* = 1.2 Hz, 1H); ^13^C NMR (125 MHz, DMSO-*d*_6_, 300 K): δ (ppm) 166.4, 155.1, 140.2, 138.4, 135.8, 132.3, 131.3, 130.8), 130.5, 130.3, 129.0, 127.0, 125.7, 125.6, 125.3, 124.9, 123.9, 121.6, 120.3; HR-MS: calcd for C_20_H_14_Cl_2_N_6_O_3_SNa [M + Na]^+^, 511.0117; found, 511.0074; Elemental analysis calcd (%) for C_20_H_14_Cl_2_N_6_O_3_S: C, 49.09; H, 2.88; N, 17.17; S, 6.55. Found: C, 48.82; H, 3.00; N, 16.92; S, 7.24.

#### 3-(*N*-{2-[(3,4-Dichlorophenyl)carbamoyl]phenyl}sulfamoyl)benzoic Acid (**6**)

**6** was synthesized according to general procedure B using **78** (200 mg, 0.71 mmol, 1.00 equiv) and 3-(chlorosulfonyl)benzoic acid (204 mg, 0.92 mmol, 1.30 equiv). After 3 h, the reaction was stopped by addition of 1 M HCl (aq 60 mL) and the mixture was subsequently extracted with EtOAc (3 × 50 mL). The combined organic layers were successively washed with a saturated NaHCO_3_ solution (aq 30 mL) and a saturated NaCl solution (aq 30 mL). After drying over MgSO_4_, filtration and removal of the solvent under reduced pressure, the crude product was purified by column chromatography [cyclohexane/EtOAc 90:10 → 15:85 over 20 min, addition of formic acid (approximately 0.5%) to both solvents], yielding **6** (68 mg, 0.15 mmol, 21%) as a yellowish-white solid. mp 245.0 °C; ^1^H NMR (500 MHz, DMSO-*d*_6_, 300 K): δ (ppm) 13.29 (br s, 1H), 10.55 (br s, 2H), 8.23 (s, 1H), 8.06 (d, ^3^*J* = 6.9 Hz, 1H), 7.99 (s, 1H), 7.91 (d, ^3^*J* = 6.9 Hz, 1H), 7.71 (d, ^3^*J* = 6.6 Hz, 1H), 7.62–7.54 (m, 3H), 7.46 (dd, ^3^*J* = 7.4 Hz, 1H), 7.31 (d, ^3^*J* = 7.4 Hz, 1H), 7.23 (m, 1H); ^13^C NMR (125 MHz, DMSO-*d*_6_, 300 K): δ (ppm) 166.3, 165.6, 139.5, 138.5, 133.5, 132.1, 131.7, 131.0, 130.7, 130.6, 130.4, 129.8, 129.0, 127.3, 126.9, 125.5, 125.1, 123.4, 121.6, 120.4. Because the qNMR sample was used to record the ^13^C NMR spectrum, the signals for maleic acid also appear in the spectrum (166.7, 135.7); HR-MS: calcd for C_20_H_14_Cl_2_N_2_O_5_SH [M + H]^+^, 511.0117; found, 511.0074; purity was determined by qNMR using maleic acid as an internal standard: 96.04%.

#### 2-(Benzo[*c*][1,2,5]oxadiazole-4-sulfonamido)-*N*-(3,4-dichlorophenyl)benzamide (**7**)

**7** was synthesized according to general procedure A using **78** (250 mg, 0.89 mmol, 1.00 equiv) and 2,3,1-benzoxadiazole-4-sulfonyl chloride (253 mg, 1.16 mmol, 1.30 equiv). After 18 h, the reaction mixture was taken up in DCM (50 mL) and the organic layer was successively washed with H_2_O (20 mL), 1 M HCl (aq 20 mL), and a saturated NaCl solution (aq 25 mL). The combined aqueous layers were once again extracted with DCM (10 mL) and the combined organic layers were dried over MgSO_4_, filtered, and the solvent was removed under reduced pressure. After column chromatographic purification (cyclohexane/EtOAc 100:0 → 0:100 over 35 min) of the crude product, **7** (394 mg, 0.85 mmol, 96%) was obtained as a yellow solid. mp 235.3 °C.; ^1^H NMR (500 MHz, DMSO-*d*_6_, 300 K): δ (ppm) 10.64 (s, 1H), 10.39 (s, 1H), 8.26 (dd, ^3^*J* = 9.2 Hz, ^4^*J* = 0.6 Hz, 1H), 8.02 (dd, ^3^*J* = 6.9 Hz, ^4^*J* = 0.6 Hz, 1H), 7.84 (d, ^4^*J* = 2.3 Hz, 1H), 7.63 (dd, ^3^*J* = 9.2 Hz, ^3^*J* = 6.9 Hz, 1H), 7.60–7.58 (m, 2H), 7.54–7.49 (m, 2H), 7.43 (dd, ^3^*J* = 8.9 Hz, ^4^*J* = 2.3 Hz, 1H), 7.29 (dd, ^3^*J* = 7.4 Hz, ^3^*J* = 6.9 Hz, 1H); ^13^C NMR (125 MHz, DMSO-*d*_6_, 300 K): δ (ppm) 166.0, 149.2, 143.9, 138.4, 135.1, 134.9, 132.1, 131.4, 130.8, 130.4, 128.8, 127.2, 127.0, 125.5, 125.4, 123.9, 121.8, 121.3, 120.2; HR-MS: calcd for C_19_H_12_Cl_2_N_4_O_4_SH [M + H]^+^, 463.0029; found, 463.0007; Elemental analysis calcd (%) for C_19_H_12_Cl_2_N_4_O_4_S: C, 49.26; H, 2.61; N, 12.09; S, 6.92. Found: C, 49.31; H, 2.75; N, 12.04; S, 6.87.

#### 2-[(4-Chloro-3-nitrophenyl)sulfonamido]-*N*-(3,4-dichlorophenyl)benzamide (**11**)

**11** was synthesized according to general procedure B using **78** (200 mg, 0.71 mmol, 1.00 equiv) and 4-chloro-3-nitrobenzenesulfonyl chloride (263 mg, 1.03 mmol, 1.30 equiv). After stirring the reaction mixture at room temperature for 3 h, 1 M HCl (aq 50 mL) was added, and the mixture was subsequently extracted with DCM (50 mL). The aqueous layer was extracted with DCM (2 × 20 mL) and the combined organic layers were successively washed with a saturated NaHCO_3_ solution (aq 30 mL), a saturated NaCl solution (aq 30 mL), dried over MgSO_4_, filtered, and concentrated under reduced pressure. **11** (160 mg, 0.32 mmol, 45%) was obtained as a beige solid after column chromatographic purification (cyclohexane/EtOAc 100:0 → 60:40 over 35 min). mp 203.9 °C; ^1^H NMR (500 MHz, DMSO-*d*_6_, 300 K): δ (ppm) 10.44 (s, 1H), 10.39 (s, 1H) 8.34 (d, ^4^*J* = 2.3 Hz, 1H), 7.96 (d, ^4^*J* = 2.3 Hz, 1H), 7.91 (dd, ^3^*J* = 8.3 Hz, ^4^*J* = 2.3 Hz, 1H), 7.82 (d, ^3^*J* = 8.3 Hz, 1H), 7.65 (d, ^3^*J* = 7.7 Hz, 1H), 7.59 (d, ^3^*J* = 8.6 Hz, 1H), 7.55–7.52 (m, 2H), 7.37–7.34 (m, 2H); ^13^C NMR (125 MHz, DMSO-*d*_6_, 300 K): δ (ppm) 165.9, 146.9, 139.6, 138.7, 134.2, 133.1, 131.9, 131.5, 130.9, 130.4, 130.0, 129.4, 129.0, 126.1, 125.5, 125.3, 124.3, 121.1, 119.9; HR-MS: calculated for C_19_H_12_Cl_3_N_3_O_5_SNa [M + Na]^+^, 523.9428; found, 523.9418; Elemental analysis calcd (%) for C_19_H_12_Cl_3_N_3_O_5_S: C, 45.57; H, 2.42; N, 8.39; S, 6.40. Found: C, 45.96; H, 2.81; N, 8.17; S, 6.33.

#### 5-Chloro-*N*-(3,4-dichlorophenyl)-2-[(3-nitrophenyl)sulfonamido]benzamide (**12**)

**12** was synthesized according to general procedure B utilizing **79** (250 mg, 0.79 mmol, 1.00 equiv) and 3-nitrobenzenesulfonyl chloride (228 mg, 1.03 mmol, 1.30 equiv). After 1.5 and 3 h reaction time, another two portions of 0.3 equiv 3-nitrobenzenesulfonyl chloride (53 mg, 0.24 mmol) each were added, and the reaction mixture was stirred for 2 additional hours. Upon addition of 1 M HCl (aq 50 mL), the reaction mixture was extracted with DCM (1 × 50, 2 × 20 mL). The combined organic layers were successively washed with a saturated NaHCO_3_ solution (aq 30 mL) and a saturated NaCl solution (aq 30 mL), dried over MgSO_4_, filtered, and the solvent was removed under reduced pressure. **12** (172 mg, 0.34 mmol, 43%) was isolated as a beige solid after purification by column chromatography (cyclohexane/EtOAc 100:0 → 50:50 over 30 min). mp 221.3 °C; ^1^H NMR (500 MHz, DMSO-*d*_6_, 300 K): δ (ppm) 10.48 (s, 1H), 10.37 (s. 1H), 8.41 (dd, ^4^*J* = 2.0 Hz, 1H), 8.33 (ddd, ^3^*J* = 8.3 Hz, ^4^*J* = 2.3 Hz, ^4^*J* = 1.2 Hz, 1H), 8.08 (ddd, ^3^*J* = 7.8 Hz, ^4^*J* = 1.7 Hz, ^4^*J* = 1.2 Hz, 1H), 7.93 (d, ^4^*J* = 2.6 Hz, 1H), 7.75 (dd, ^3^*J* = 8.0 Hz, 1H), 7.70 (d, ^4^*J* = 2.6 Hz, 1H), 7.58 (d, ^3^*J* = 8.9 Hz, 1H), 7.58 (dd, ^3^*J* = 8.7 Hz, ^4^*J* = 3.2 Hz, 1H), 7.47 (dd, ^3^*J* = 8.9 Hz, ^4^*J* = 2.6 Hz, 1H), 7.33 (d, ^3^*J* = 8.9 Hz, 1H); ^13^C NMR (125 MHz, DMSO-*d*_6_, 300 K): δ (ppm) 164.3, 147.6, 140.8, 138.5, 133.3, 132.7, 131.5, 131.3, 131.2, 130.8, 130.4, 130.2, 128.7, 127.5, 127.3, 125.5, 121.6, 121.2, 120.0; HR-MS: calcd for C_19_H_12_Cl_3_N_3_O_5_SH [M + H]^+^, 499.9636; found, 499.9625; Elemental analysis calcd (%) for C_19_H_12_Cl_3_N_3_O_5_S: C, 45.57; H, 2.42; N, 8.39; S, 6.40. Found: C, 45.77; H, 2.55; N, 8.28; S, 6.06.

#### 5-Chloro-2-[(4-chloro-3-nitrophenyl)sulfonamido]-*N*-(3,4-dichlorphenyl)benzamide (**13**)

**13** was synthesized according to general procedure B using **79** (250 mg, 0.79 mmol, 1.00 equiv) and 4-chloro-3-nitro-benzenesulfonyl chloride (263 mg, 1.03 mmol, 1.30 equiv). After 1.5 h, another 0.3 equiv 4-chloro-3-nitrobenzenesulfonyl chloride (61 mg, 0.24 mmol) was added, and the reaction mixture was stirred for an additional 3 h. Subsequently, 1 M HCl (aq 50 mL) was added, and the mixture was extracted with DCM (1 × 50, 2 × 20 mL). The combined organic layers were successively washed with a saturated NaHCO_3_ solution (aq 30 mL) and a saturated NaCl solution (aq 30 mL), dried over MgSO_4_, and then filtered. The solvent was removed under reduced pressure and the crude product was purified by column chromatography (cyclohexane/EtOAc 100:0 → 60:40 over 35 min), yielding **13** (194 mg, 0.36 mmol, 46%) as a light brown solid. mp 182.8 °C; ^1^H NMR (500 MHz, DMSO-*d*_6_, 300 K): δ (ppm) 10.47 (s, 1H), 10.37 (s, 1H), 8.29 (d, ^4^*J* = 2.3 Hz, 1H), 7.89 (d, ^4^*J* = 2.3 Hz, 1H), 7.87 (dd, ^3^*J* = 8.6 Hz, ^4^*J* = 2.3 Hz, 1H), 7.79 (d, ^3^*J* = 8.6 Hz, 1H), 7.68 (d, ^4^*J* = 2.6 Hz, 1H), 7.57–7.54 (m, 2H), 7.46 (dd, ^3^*J* = 8.9 Hz, ^4^*J* = 2.3 Hz, 1H), 7.32 (d, ^3^*J* = 8.6 Hz, 1H); ^13^C NMR (125 MHz, DMSO-*d*_6_, 300 K): δ (ppm) 164.3, 147.0, 139.6, 138.5, 133.2, 133.2, 131.6, 131.5, 131.0, 130.5, 130.2, 128.7, 128.7, 127.9, 125.6, 124.3, 121.2, 121.2, 119.9; HR-MS: calcd for C_19_H_11_Cl_4_N_3_O_5_SH [M + H]^+^, 535.9217; found, 535.9200; purity was determined by qNMR using maleic acid as an internal standard: 95.4%.

#### *N*-(2,4-Dichlorophenyl)-2-[(3-nitrophenyl)sulfonamido]benzamide (**20**)

**20** was prepared according to general procedure C reacting **76** (200 mg, 0.62 mmol, 1.00 equiv) with HOBt monohydrate (142 mg, 0.93 mmol, 1.50 equiv), EDC-HCl (144 mg, 0.93 mmol, 1.50 equiv), 2,4-dichloroaniline (201 mg, 1.24 mmol, 2.00 equiv), and DIPEA (160 mg, 1.24 mmol, 2.00 equiv, 0.21 mL) in dry DMF (5 mL). The mixture was stirred for 72 h, 1 M HCl (aq 10 mL) was added, and the mixture was extracted with DCM (3 × 20 mL). The combined organic layers were successively washed with a 2 M K_2_CO_3_ solution (aq 20 mL) and a 5% LiCl solution (aq 5 × 10 mL), dried over MgSO_4_, filtered, and the solvent removed under reduced pressure. The crude product was purified by column chromatography (cyclohexane/EtOAc 100:0 → 50:50 over 25 min) resulting in **20** (84.2 mg, 0.18 mmol, 29%) as a white solid. mp 200.1 °C; ^1^H NMR (500 MHz, DMSO-*d*_6_, 300 K): δ (ppm) 10.89 (s, 1H), 10.24 (s, 1H), 8.46 (dd, ^3^*J* = 8.2 Hz, ^4^*J* = 1.3 Hz, 1H), 8.40 (dd, ^4^*J* = 2.0 Hz, 1H), 8.14 (ddd, ^3^*J* = 8.0 Hz, ^4^*J* = 1.7 Hz, ^4^*J* = 1.2 Hz, 1H), 7.85 (d, ^3^*J* = 8.0 Hz, 1H), 7.84 (dd, ^3^*J* = 8.0 Hz, 1H), 7.72 (d, ^4^*J* = 2.6 Hz, 1H), 7.66 (d, ^3^*J* = 7.7 Hz, 1H), 7.55 (dd, ^3^*J* = 7.5 Hz, 1H), 7.49 (dd, ^3^*J* = 8.7 Hz, ^4^*J* = 2.4 Hz, 1H), 7.36 (dd, ^3^*J* = 8.0 Hz, ^4^*J* = 0.9 Hz, 1H), 7.32 (br s, 1H); ^13^C NMR (125 MHz, DMSO-*d*_6_, 300 K): δ (ppm) 166.3, 147.1, 140.4, 136.0, 133.5, 132.7, 132.7, 131.4, 130.8, 129.6, 129.5, 129.0, 128.6, 127.8, 127.6, 125.3, 125.1, 123.0, 121.5; HR-MS: calcd for C_19_H_13_Cl_2_N_3_O_5_SNa [M + Na]^+^, 487.9845; found, 487.9834; Elemental analysis calcd (%) for C_19_H_13_Cl_2_N_3_O_5_S: C, 48.94; H, 2.81; N, 9.01; S, 6.88. Found: C, 48.86; H, 2.83; N, 8.91; S, 6.46.

#### *N*-(3,4-Dichlorophenyl)-3-[(3-nitrophenyl)sulfonamido]propanamide (**21**)

According to general procedure B, **87** (250 mg, 1.07 mmol, 1.00 equiv) and 3-nitrobenzenesulfonyl chloride (308 mg, 1.39 mmol, 1.30 equiv) were reacted for 16 h at room temperature. Subsequently, 1 M HCl (aq 60 mL) was added, and the mixture was extracted with EtOAc (2 × 50 mL). The organic layer was washed with a saturated NaHCO_3_ solution (aq 30 mL) and a saturated NaCl solution (aq 30 mL), dried over MgSO_4_, filtered, and the solvent removed under reduced pressure. Column chromatography (cyclohexane/EtOAc 90:10 → 15:85 over 30 min) rendered **21** (225 mg, 0.54 mmol, 51%) as a white solid. mp 191.3 °C; ^1^H NMR (500 MHz, DMSO-*d*_6_, 300 K): δ (ppm) 10.17 (s, 1H), 8.53 (dd, ^4^*J* = 2.0 Hz, 1H), 8.43 (ddd, ^3^*J* = 8.3 Hz, ^4^*J* = 2.3 Hz, ^4^*J* = 0.9 Hz, 1H), 8.22 (ddd, ^3^*J* = 8.0 Hz, ^4^*J* = 1.7 Hz, ^4^*J* = 1.2 Hz, 1H), 8.14 (br s, 1H), 7.91 (d, ^4^*J* = 2.6 Hz, 1H), 7.88 (dd, ^3^*J* = 8.0 Hz, 1H), 7.52 (d, ^3^*J* = 8.9 Hz, 1H), 7.40 (dd, ^3^*J* = 8.9 Hz, ^4^*J* = 2.6 Hz, 1H), 3.14 (t, ^3^*J* = 6.9 Hz, 2H), 2.57–2.45 (m, 2H, overlaid with DMSO-*d*_6_); ^13^C NMR (125 MHz, DMSO-*d*_6_, 300 K): δ (ppm) 169.0, 147.8, 142.2, 139.0, 132.5, 131.2, 130.8, 130.5, 126.9, 124.5, 121.3, 120.2, 119.0, 38.5, 36.5; HR-MS: calcd for C_15_H_13_Cl_2_N_3_O_5_SNa [M + Na]^+^, 439.9846; found, 439.9843; Elemental analysis calcd (%) for C_15_H_13_Cl_2_N_3_O_5_S: C, 43.07; H, 3.13; N, 10.05; S, 7.67. Found: C, 43.11; H, 3.42; N, 9.74; S, 7.28.

#### *N*-(3,4-Dichlorophenyl)-2-[(*N*-methyl-3-nitrophenyl)sulfonamido]benzamide (**22**)

**22** was synthesized according to general procedure B using **89** (250 mg, 0.85 mmol, 1.00 equiv) and 3-nitrobenzenesulfonyl chloride (244 mg, 1.10 mmol, 1.30 equiv). Additionally, a catalytic amount of DMAP (5 mg, 0.04 mmol, 0.05 equiv) was added. The reaction mixture was stirred for 18 h at room temperature, then two additional portions of 0.5 equiv 3-nitro-benzenesulfonyl chloride each (94 mg, 0.43 mmol) were added within 4 h. The reaction mixture was stirred for an additional 18 h and subsequently the pyridine was removed under reduced pressure. The reddish residue was taken up in DCM (50 mL) and successively washed with 1 M HCl (aq 2 × 15 mL) and a saturated NaCl solution (aq 20 mL). The organic layer was dried over MgSO_4_ and the solvent removed under reduced pressure. The crude product was purified by column chromatography (cyclohexane/EtOAc 100:0 → 25:75 over 30 min), yielding **22** (190 mg, 0.40 mmol, 47%) as a fine faint purple powder. mp 164.2 °C; ^1^H NMR (500 MHz, DMSO-*d*_6_, 300 K): δ (ppm) 10.54 (s, 1H), 8.38 (ddd, ^3^*J* = 8.3 Hz, ^4^*J* = 2.3 Hz, ^4^*J* = 1.2 Hz, 1H), 8.22 (dd, ^4^*J* = 2.0 Hz, 1H), 8.09 (ddd, ^3^*J* = 7.7 Hz, ^4^*J* = 1.7 Hz, ^4^*J* = 0.9 Hz, 1H), 7.97 (d, ^4^*J* = 2.3 Hz, 1H), 7.82 (dd, ^3^*J* = 7.9 Hz, 1H), 7.64–7.62 (m, 1H), 7.56 (d, ^3^*J* = 8.6 Hz, 1H), 7.54–7.50 (m, 3H), 7.14–7.12 (m, 1H), 3.33 (s, 3H); ^13^C NMR (125 MHz, DMSO-*d*_6_, 300 K): δ (ppm) 165.6, 147.6, 139.6, 139.1, 137.7, 137.0, 133.1, 131.3, 131.2, 130.8, 130.5, 129.1, 128.9, 128.7, 127.4, 125.0, 121.7, 120.5, 119.4, 40.7; HR-MS: calcd for C_20_H_15_Cl_2_N_3_O_5_SH [M + H]^+^, 480.0182; found, 480.0167; Elemental analysis calcd (%) for C_20_H_15_Cl_2_N_3_O_5_S: C, 50.01; H, 3.15; N, 8.75; S, 6.68. Found: C, 49.91; H, 3.23; N, 8.64; S, 6.29.

#### *N*-(3,4-Dichlorophenyl)-2-(3-nitrobenzamido)benzamide (**23**)

In a thoroughly dried N_2_ flask, 3-nitrobenzoic acid (214 mg, 1.28 mmol, 1.20 equiv) was stirred in thionyl chloride (3 mL) at 110 °C under an argon atmosphere. After 3.5 h, the excess thionyl chloride was removed in vacuo, and the yellowish oily residue was taken up in dry THF (3 mL), while keeping under an argon atmosphere. This solution was added dropwise over 5 min to a stirred mixture of **78** (300 mg, 1.07 mmol, 1.00 equiv) and TEA (0.45 mL, 3.21 mmol, 3.00 equiv) in dry THF (2 mL) at 0 °C. The reaction mixture was allowed to come to room temperature and was stirred for 18 h. The solvent was removed under reduced pressure and the residue taken up in H_2_O (25 mL) and extracted with DCM (3 × 25 mL). The combined organic layers were successively washed with 1 M HCl (aq 10 mL), a saturated NaHCO_3_ solution (aq 15 mL), and a saturated NaCl solution (20 mL). After drying over MgSO_4_ and removal of the solvent under reduced pressure, the crude product was purified by column chromatography (cyclohexane/EtOAc 100:0 → 30:70 over 30 min), yielding **23** (323 mg, 0.75 mmol, 70%) as a gray solid. mp 218.6 °C; ^1^H NMR (500 MHz, DMSO-*d*_6_, 300 K): δ (ppm) 11.32 (s, 1H), 10.71 (s, 1H), 8.71 (dd, ^4^*J* = 2.0 Hz, 1H), 8.44 (ddd, ^3^*J* = 8.3 Hz, ^4^*J* = 2.3 Hz, ^4^*J* = 1.2 Hz, 1H), 8.32 (ddd, ^3^*J* = 7.7 Hz, ^4^*J* = 1.7 Hz, ^4^*J* = 0.9 Hz, 1H), 8.11 (d, ^3^*J* = 8.0 Hz, 1H), 8.07 (d, ^4^*J* = 2.3 Hz, 1H), 7.86 (dd, ^3^*J* = 8.0 Hz, 1H), 7.84 (dd, ^3^*J* = 7.9 Hz, ^4^*J* = 1.4 Hz, 1H), 7.68 (dd, ^3^*J* = 8.9 Hz, ^4^*J* = 2.3 Hz, 1H), 7.63 (ddd, ^3^*J* = 8.3 Hz, ^3^*J* = 7.8 Hz, ^4^*J* = 1.4 Hz, 1H), 7.59 (d, ^3^*J* = 8.9 Hz, 1H), 7.36 (ddd, ^3^*J* = 7.7 Hz, ^3^*J* = 7.6 Hz, ^4^*J* = 1.2 Hz, 1H); ^13^C NMR (125 MHz, DMSO-*d*_6_, 300 K): δ (ppm) 167.0, 163.0, 147.9, 139.0, 137.0, 135.9, 133.4, 131.9, 130.8, 130.5, 130.5, 129.0, 126.3, 126.0, 125.3, 124.4, 123.1, 122.1, 121.7, 120.4; HR-MS: calcd for C_20_H_13_Cl_2_N_3_O_4_Na [M + Na]^+^, 452.0175; found, 452.0166; Elemental analysis calcd (%) for C_20_H_13_Cl_2_N_3_O_4_: C: 55.83; H, 3.05; N, 9.77. Found: C, 55.76; H, 3.30; N, 9.61.

#### *N*-(3,4-Dichlorophenyl)-3-[(3-nitrophenyl)sulfonamido]thiophene-2-carboxamide (**24**)

**24** was prepared according to general procedure C using **90** (300 mg, 0.91 mmol, 1.00 equiv), HOBt monohydrate (210 mg, 1.37 mmol, 1.50 equiv), EDC-HCl (213 mg, 1.37 mmol, 1.50 equiv), and dry DMF (5 mL). The reaction mixture was stirred for 2 h at room temperature under an argon atmosphere, then 3,4-dichloroaniline (297 mg, 1.83 mmol, 2.00 equiv) and DIPEA (237 mg, 1.83 mmol, 2.00 equiv, 0.31 mL) were added. The reaction mixture was stirred for 40 h at room temperature and then for an additional 12 h at 50 °C. After completion of the reaction, the solvent was removed under reduced pressure, and the remaining residue was taken up in H_2_O (10 mL) and extracted with EtOAc (3 × 20 mL). The combined organic layers were successively washed with 1 M HCl (aq 20 mL), a 2 M K_2_CO_3_ solution (20 mL), a 5% LiCl solution (aq 5 × 10 mL), a saturated NaCl solution (20 mL), and the organic layer was finally dried over MgSO_4_. The solvent was removed under reduced pressure and the crude product was purified by column chromatography (cyclohexane/EtOAc 100:0 → 50:50 over 30 min), furnishing **24** (140 mg, 0.30 equiv, 33%) as a beige solid. mp 211.9 °C; ^1^H NMR (500 MHz, DMSO-*d*_6_, 300 K): δ (ppm) 10.66 (br s, 1H), 10.31 (s, 1H), 8.52 (dd, ^4^*J* = 2.0 Hz, ^4^*J* = 1.7 Hz 1H), 8.42 (dd, ^3^*J* = 8.2 Hz, ^4^*J* = 1.3 Hz, 1H), 8.20 (d, ^3^*J* = 8.0 Hz, 1H), 7.93 (d, ^4^*J* = 2.3 Hz, 1H), 7.83 (dd, ^3^*J* = 8.0 Hz, 1H), 7.80 (d, ^3^*J* = 5.2 Hz, 1H), 7.59 (d, ^3^*J* = 8.9 Hz, 1H), 7.54 (dd, ^3^*J* = 8.9 Hz, ^4^*J* = 2.3 Hz, 1H), 7.11 (d, ^3^*J* = 5.2 Hz, 1H); ^13^C NMR (125 MHz, DMSO-*d*_6_, 300 K): δ (ppm) 161.0, 147.8, 140.9, 138.7, 138.2, 132.6, 131.4, 130.8, 130.5, 130.4, 127.7, 125.6, 123.1, 121.8, 121.5, 120.6, 120.1; HR-MS: calcd for C_17_H_11_Cl_2_N_3_O_5_S_2_ [M + H]^+^, 471.9590; found, 471.9589; Elemental analysis calcd (%) for C_17_H_11_Cl_2_N_3_O_5_S_2_: C, 43.23; H, 2.35; N, 8.90; S, 13.58. Found: C, 43.45; H, 2.42; N, 8.80; S, 13.40.

#### Methyl-3-[(3-nitrophenyl)sulfonamido]thiophene-2-carboxylate (**25**)

To a solution of 3-nitrobenzenesulfonyl chloride (508 mg, 2.29 mmol, 1.20 equiv) in THF (10 mL) was added pyridine (151 mg, 1.91 mmol, 1.00 equiv, 0.15 mL). Subsequently, methyl 3-aminothiophene-2-carboxylate (300 mg, 1.91 mmol, 1.00 equiv) was added in portions, and the reaction mixture was stirred for 24 h at 75 °C and then for a further 48 h at room temperature. After addition of 1 M HCl (aq 20 mL), the mixture was extracted with EtOAc (3 × 20 mL), the combined organic layers were successively washed with H_2_O (20 mL) and a saturated NaCl solution (aq 20 mL), dried over MgSO_4_, filtered, and the solvent removed under reduced pressure. Column chromatography (cyclohexane/EtOAc 100:0 → 50:50 over 20 min) afforded **25** (270 mg, 0.79 mmol, 41%) as a beige solid. mp 243.3 °C; ^1^H NMR (500 MHz, DMSO-*d*_6_, 300 K): δ (ppm) 10.26 (s, 1H), 8.61 (dd, ^4^*J* = 2.0 Hz, 1H), 8.49 (ddd, ^3^*J* = 8.3 Hz, ^4^*J* = 2.3 Hz, ^4^*J* = 1.2 Hz, 1H), 8.28 (ddd, ^3^*J* = 8.0 Hz, ^4^*J* = 2.0 Hz, ^4^*J* = 1.2 Hz, 1H), 7.89 (dd, ^3^*J* = 8.3 Hz, 1H), 7.87 (d, ^3^*J* = 5.4 Hz, 1H), 7.18 (d, ^3^*J* = 5.4 Hz, 1H), 3.71 (s, 3H); ^13^C NMR (125 MHz, DMSO-*d*_6_, 300 K): δ (ppm) 161.9, 147.9, 140.8, 139.9, 133.1, 132.7, 131.4, 127.9, 122.8, 121.7, 115.1, 52.0; HR-MS: calcd for C_12_H_10_N_2_O_6_S_2_Na [M + Na]^+^, 364.9872; found, 364.9871; Elemental analysis calcd (%) for C_12_H_10_N_2_O_6_S_2_: C, 42.10; H, 2.94; N, 8.18; S, 18.73. Found: C, 42.12; H, 3.05; N, 8.12; S, 18.83.

#### Methyl-2-[(3-nitrophenyl)sulfonamido]benzoate (**28**)

To a solution of 3-nitrobenzenesulfonyl chloride (500 mg, 2.26 mmol, 1.00 equiv) in THF (6 mL), pyridine (179 mg, 2.26 mmol, 1.00 equiv, 0.18 mL), and methyl anthranilate (342 mg, 2.26 mmol, 1.00 equiv, 0.29 mL) were added slowly. The resulting solution was stirred for 18 h at rt and subsequently heated to 50 °C for an additional 4 h. The solvent was removed under reduced pressure, the oily residue taken up in H_2_O and the aqueous layer was adjusted to pH 3–4 with 1 M HCl (aq), whereupon a reddish solid precipitated. The suspension was stirred intensively for 30 min and subsequently the solid was filtered off, washed with H_2_O (3 × 5 mL), and dried in vacuo, affording **28** (376 mg, 1.12 mmol, 50%). mp 100.4 °C; ^1^H NMR (500 MHz, DMSO-*d*_6_, 300 K): δ (ppm) 10.49 (s, 1H), 8.47 (ddd, ^3^*J* = 8.0 Hz, ^4^*J* = 2.3 Hz, ^4^*J* = 1.2 Hz, 1H), 8.45 (dd, ^4^*J* = 2.0 Hz, 1H), 8.17 (ddd, ^3^*J* = 7.8 Hz, ^4^*J* = 1.7 Hz, ^4^*J* = 1.2 Hz, 1H), 7.86 (dd, ^3^*J* = 8.0 Hz, 1H), 7.80 (dd, ^3^*J* = 7.7 Hz, ^4^*J* = 1.4 Hz, 1H), 7.58 (ddd, ^3^*J* = 8.3 Hz, ^3^*J* = 7.5 Hz, ^4^*J* = 1.7 Hz, 1H), 7.41 (dd, ^3^*J* = 8.3 Hz, ^4^*J* = 0.9 Hz, 1H), 7.26 (td, ^3^*J* = 7.7 Hz, ^4^*J* = 1.2 Hz, 1H), 3.76 (s, 3H); ^13^C NMR (125 MHz, DMSO-*d*_6_, 300 K): δ (ppm) 166.9, 147.8, 140.6, 136.7, 134.0, 132.7, 131.4, 131.0, 127.8), 125.8, 122.4, 121.6, 121.0, 52.4; HR-MS: calcd for C_14_H_12_N_2_O_6_SNa [M + Na]^+^, 359.0308; found, 359.0306; Elemental analysis calcd (%) for C_14_H_12_N_2_O_6_S: C, 50.00; H, 3.60; N, 8.33; S, 9.53. Found: C, 49.91; H, 3.73; N, 8.31; S, 9.28.

#### 2-[(3-Nitrophenyl)sulfonamido]benzoic Acid (**76**)

**28** (350 mg, 1.04 mmol, 1.00 equiv) was suspended in EtOH (2 mL) and NaOH (83 mg, 2.08 mmol, 2.00 equiv), dissolved in H_2_O (2 mL), was added. The reaction mixture was stirred for 16 h at 85 °C. The solvent was removed under reduced pressure and the residue was taken up in H_2_O (5 mL). A pH 2 was adjusted by addition of 37% HCl (aq), whereupon a white solid precipitated. The solid was filtered off, washed with 1 M HCl (aq 3 × 3 mL), and subsequently dried in vacuo, which furnished **76** (308 mg, 0.96 mmol, 92%) as a white solid. mp 217.6 °C; ^1^H NMR (500 MHz, DMSO-*d*_6_, 300 K): δ (ppm) 13.75 (br s, 1H), 11.13 (s, 1H), 8.49 (dd, ^4^*J* = 2.0 Hz, 1H), 8.46 (ddd, ^3^*J* = 8.3 Hz, ^4^*J* = 2.3 Hz, ^4^*J* = 1.2 Hz, 1H), 8.20 (ddd, ^3^*J* = 8.0 Hz, ^4^*J* = 1.7 Hz, ^4^*J* = 0.9 Hz, 1H), 7.89 (dd, ^3^*J* = 8.0 Hz, ^4^*J* = 1.4 Hz, 1H), 7.85 (dd, ^3^*J* = 8.3 Hz, 1H), 7.57 (ddd, ^3^*J* = 8.0 Hz, ^3^*J* = 7.5 Hz, ^4^*J* = 1.7 Hz, 1H), 7.49 (dd, ^3^*J* = 8.3 Hz, ^4^*J* = 0.9 Hz, 1H), 7.19 (td, ^3^*J* = 7.7 Hz, ^4^*J* = 1.2 Hz, 1H); ^13^C NMR (125 MHz, DMSO-*d*_6_, 300 K): δ (ppm) 169.2, 147.9, 140.3, 138.5, 134.4, 132.7, 131.5, 131.5, 128.0, 124.3, 121.6, 120.0, 118.6; HR-MS: calcd for C_13_H_10_N_2_O_6_SNa [M + Na]^+^, 345.0152; found, 345.0151.

#### 2-Amino-*N*-(3,4-dichlorophenyl)benzamide (**78**)

Isatoic anhydride (5.00 g, 30.65 mmol, 1.00 equiv) was dissolved in glacial acetic acid (70 mL) and 3,4-dichloroaniline (9.93 g, 61.3 mmol, 2.00 equiv), dissolved in glacial acetic acid (65 mL), was added over a period of 5 min. The reaction mixture was stirred at 105 °C for 18 h under reflux and the reaction was monitored by TLC. After cooling to room temperature, the mixture was poured into H_2_O (500 mL), upon which a precipitate formed. The resulting suspension was neutralized with a saturated NaHCO_3_ solution (aq) and then extracted with DCM (250 mL). The organic layer was dried over MgSO_4_, filtered, and the solvent removed under reduced pressure. The mixture was purified by column chromatography (cyclohexane/EtOAc 85:15 over 45 min). Due to incomplete separation, the slightly contaminated product was recrystallized from a mixture of H_2_O/ethanol (1:1) to afford **78** (2.50 g, 8.89 mmol, 29%) as milky white needle-like crystals. mp 142.1 °C; ^1^H NMR (500 MHz, DMSO-*d*_6_, 300 K): δ (ppm) 10.20 (s, 1H), 8.11 (d, ^4^*J* = 2.3 Hz, 1H), 7.70 (dd, ^3^*J* = 8.9 Hz, ^4^*J* = 2.3 Hz, 1H), 7.62 (dd, ^3^*J* = 7.9 Hz, ^4^*J* = 1.4 Hz, 1H), 7.58 (d, ^3^*J* = 8.9 Hz, 1H), 7.22 (ddd, ^3^*J* = 8.3 Hz, ^3^*J* = 7.2 Hz, ^4^*J* = 1.4 Hz, 1H), 6.76 (dd, ^3^*J* = 8.3 Hz, ^4^*J* = 1.2 Hz, 1H), 7.22 (ddd, ^3^*J* = 8.0 Hz, ^3^*J* = 7.2 Hz, ^4^*J* = 1.2 Hz, 1H), 6.36 (s, 2H); ^13^C NMR (125 MHz, DMSO-*d*_6_, 300 K): δ (ppm) 168.0, 149.9, 139.4, 132.5, 130.7, 130.3, 128.6, 124.6, 121.4, 120.2, 116.5, 114.6, 114.3; HR-MS: calcd for C_13_H_10_Cl_2_N_2_OH [M + H]^+^, 281.0243; found, 281.0247; purity was determined by qNMR using maleic acid as an internal standard: 98.9%.

#### 2-Amino-5-chloro-*N*-(3,4-dichlorophenyl)benzamide (**79**)

**83** (2.40 g, 5.77 mmol, 1.00 equiv) was dissolved in DCM (50 mL), then TFA (6.58 g, 57.73 mmol, 10.00 equiv, 4.45 mL) was added, and the reaction mixture was stirred intensively for 3.5 h at room temperature. After completion of the reaction, the reaction mixture was washed with a saturated NaHCO_3_ solution (aq) until the aqueous layer remained slightly basic. The organic layer was dried over MgSO_4_, filtered, and the solvent was removed under reduced pressure. After purification by column chromatography (cyclohexane/EtOAc 100:0 → 50:50 over 20 min), **79** (1.73 g, 5.48 mmol, 95%) was obtained as a yellowish solid. mp 166.9 °C; ^1^H NMR (500 MHz, DMSO-*d*_6_, 300 K): δ (ppm) 10.28 (s, 1H), 8.08 (d, ^4^*J* = 2.3 Hz, 1H), 7.64–7.73 (m, 2H), 7.59 (d, ^3^*J* = 8.9 Hz, 1H), 7.24 (dd, ^3^*J* = 8.9 Hz, ^4^*J* = 2.3 Hz, 1H), 6.79 (d, ^3^*J* = 8.9 Hz, 1H), 6.50 (s, 2H); ^13^C NMR (125 MHz, DMSO-*d*_6_, 300 K): δ (ppm) 166.7, 148.8, 139.1, 132.2, 130.7, 130.4, 127.8, 124.9, 121.6, 120.4, 118.2, 117.8, 115.1; HR-MS: calcd for C_13_H_9_Cl_3_N_2_OH [M + H]^+^, 314.9853; found, 314.9856; Elemental analysis calcd (%) for C_13_H_9_Cl_3_N_2_O: C, 49.48; H, 2.87; N, 8.88. Found: C, 49.56; H, 3.18; N, 8.89.

#### 2-Amino-5-chlorobenzoic Acid (**81**)

Methyl 2-amino-5-chloro-benzoate (2.50 g, 13.47 mmol, 1.00 equiv) was dissolved in a mixture of 2 M NaOH (aq 10 mL) and EtOH (10 mL), and the reaction mixture was stirred at 90 °C for 4 h. Subsequently, the solvent was removed under reduced pressure and H_2_O (20 mL) was added. The aqueous solution was adjusted to pH 2–3 with 1 M HCl (aq), whereupon a white precipitate formed, which was filtered off, washed with H_2_O (5 × 10 mL), and finally dried in vacuo. **81** was obtained as a beige solid (2.13 g, 12.41 mmol, 92%). mp 208.5 °C; ^1^H NMR (500 MHz, DMSO-*d*_6_, 300 K): δ (ppm) 8.71 (br s, 2H), 7.62 (d, ^4^*J* = 2.6 Hz, 1H, 7.24 (dd, ^3^*J* = 8.9 Hz, ^4^*J* = 2.6 Hz, 1H), 6.78 (d, ^3^*J* = 8.9 Hz, 1H); ^13^C NMR (125 MHz, DMSO-*d*_6_, 300 K): δ (ppm) 168.4, 150.2, 133.4, 129.8, 118.2, 117.5, 110.5; HR-MS: calcd for C_7_H_6_ClNO_2_H [M + H]^+^, 172.0160; found, 172.0153.

#### 2-[(*tert*-Butoxycarbonyl)amino]-5-chlorobenzoic Acid (**82**)

**81** (2.050 g, 11.95 mmol, 1.00 equiv) was dissolved in THF/H_2_O (1:1, 40 mL) and the pH of the solution was adjusted to 10 with 2 M NaOH (aq). Di-*tert*-butyl dicarbonate (2.868 g, 13.14 mmol, 1.10 equiv) was added and the reaction mixture was stirred for 18 h at room temperature. Subsequently, two additional portions of 0.5 equiv of di-*tert*-butyl dicarbonate (1.305 g, 5.98 mmol), each were added at intervals of 4 h and stirring was continued for 18 h. The solvent mixture was removed under reduced pressure and H_2_O (20 mL) was added to the residue. The pH of the resulting mixture was adjusted to 4 with 15% citric acid (aq), whereupon a white precipitate formed, which was filtered off, washed with H_2_O (30 mL), and dissolved in EtOAc (100 mL). The organic layer was dried over MgSO_4_ and filtered. Removal of the solvent under reduced pressure and further drying afforded **82** (2.853 g, 10.50 mmol, 88%) as a white solid. mp 178.0 °C; ^1^H NMR (500 MHz, DMSO-*d*_6_, 300 K): δ (ppm) 13.96 (br s, 1H), 10.43 (s, 1H), 8.29 (d, ^3^*J* = 9.2 Hz, 1H), 7.89 (d, ^4^*J* = 2.6 Hz, 1H), 7.62 (dd, ^3^*J* = 9.0 Hz, ^4^*J* = 2.6 Hz, 1H), 1.48 (s, 9H); ^13^C NMR (125 MHz, DMSO-*d*_6_, 300 K): δ (ppm) 168.3, 151.8, 140.3, 133.7, 130.2, 125.0, 120.0, 117.0, 80.5, 27.8; HR-MS: calcd for C_12_H_14_ClNO_4_Na [M + Na]^+^, 294.0504; found, 294.0490.

#### *tert*-Butyl-(4-chloro-2-[(3,4-dichlorophenyl)carbamoyl)phenyl]carbamate (**83**)

**82** (2.60 g, 9.57 mmol, 1.00 equiv), HBTU (4.35 mg, 11.48 mmol, 1.20 equiv), and NMM (4.84 g, 47.85 mmol, 5.00 equiv, 5.32 mL) were dissolved in dry DMF (35 mL) in a thoroughly dried N_2_ flask and stirred for 0.5 h at room temperature under an argon atmosphere. After slow addition of a solution of 3,4-dichloroaniline (1.86 g, 11.48 mmol, 1.20 equiv) in dry DMF (5 mL) to the yellowish reaction mixture, stirring was continued for 24 h at room temperature. The DMF was subsequently removed under reduced pressure, the remaining residue was taken up in DCM (100 mL) and successively washed with a 10% citric acid (aq 30 mL) solution, a saturated NaHCO_3_ solution (aq 30 mL), and a saturated NaCl solution (aq 40 mL), and finally dried over MgSO_4_. After removal of the solvent under reduced pressure, the crude product was purified by column chromatography (cyclohexane/EtOAc 100:0 → 30:70 over 30 min) and **83** (2.62 g, 6.30 mmol, 66%) was obtained as an off-white solid. mp 207.0 °C; ^1^H NMR (500 MHz, DMSO-*d*_6_, 300 K): δ (ppm) 10.67 (s, 1H), 9.72 (s, 1H), 8.06 (d, ^4^*J* = 2.6 Hz, 1H), 7.99 (d, ^3^*J* = 8.9 Hz, 1H), 7.82 (d, ^4^*J* = 2.3 Hz, 1H), 7.69–7.61 (m, 2H), 7.58 (dd, ^3^*J* = 8.9 Hz, ^4^*J* = 2.6 Hz, 1H), 1,43 (s, 9H); ^13^C NMR (125 MHz, DMSO-*d*_6_, 300 K): δ (ppm) 165.8, 152.1, 138.7, 137.4, 131.8, 130.8, 130.5, 128.3, 125.9, 125.6, 124.1, 121.9, 120.6, 80.1, 27.8; HR-MS: calcd for C_18_H_17_Cl_3_N_2_O_3_Na [M + Na]^+^, 437.0197; found, 437.0181; Elemental analysis calcd (%) for C_18_H_17_Cl_3_N_2_O_3_: C, 52.01; H, 4.12; N, 6.74. Found: C, 52.05; H, 4.00; N, 6.73.

#### 3-[(*tert*-Butoxycarbonyl)amino]propanoic Acid (**85**)

β-Alanine (1.00 g, 11.23 mmol, 1.00 equiv) and sodium hydroxide (494 mg, 12.35 mmol, 1.10 equiv) were dissolved in a dioxane/H_2_O mixture (1:1, 20 mL) and the pH of the solution was adjusted to 10 with 32% HCl (aq). Di-*tert*-butyl dicarbonate (2.695 g, 12.35 mmol, 1.10 equiv) was then added in portions and the reaction mixture was stirred for 14 h at room temperature. The solvent was removed under reduced pressure, then H_2_O (100 mL) was added, and the pH adjusted to 2–3 by adding a 10% citric acid solution (aq). The aqueous layer was washed with cyclohexane (40 mL) and then extracted with DCM (3 × 50 mL). The combined DCM layers were washed with H_2_O (30 mL), dried over MgSO_4_, filtered, and the solvent removed under reduced pressure to give **85** (1.343 g, 7.10 mmol, 63%) as a colorless solid after drying in vacuo. mp 77.1 °C; ^1^H NMR (500 MHz, DMSO-*d*_6_, 300 K): δ (ppm) 12.12 (s, 1H), 6.76 (br s, 1H), 3.12 (dt, ^3^*J* = 7.2 Hz, ^3^*J* = 6.0 Hz, 2H), 2.34 (t, ^3^*J* = 7.2 Hz, 2H), 1.37 (s, 9H); ^13^C NMR (125 MHz, DMSO-*d*_6_, 300 K): δ (ppm) 172.7, 155.4, 77.6, 36.1, 34.2, 28.2; HR-MS: calcd for C_8_H_15_NO_4_Na [M + Na]^+^, 212.0893; 212.0885.

#### *tert*-Butyl-{3-[(3,4-dichlorophenyl)amino]-3-oxopropyl}carbamate (**86**)

In a thoroughly dried N_2_ flask, **85** (1.15 g, 6.08 mmol, 1.00 equiv), HBTU (2.76 g, 7.29 mmol, 1.20 equiv), and NMM (3.08 g, 30.40 mmol, 5.00 equiv, 3.38 mL) were dissolved in dry DMF (12 mL) and stirred for 0.5 h at room temperature under an argon atmosphere. 3,4-Dichloroaniline (1.18 g, 7.29 mmol, 1.20 equiv) was added and the reaction mixture was stirred for an additional 16 h, while the reaction was monitored by TLC. The DMF was removed under reduced pressure and the residue was taken up in DCM (50 mL). The organic layer was successively washed with H_2_O (40 mL), 1 M HCl (aq 30 mL), and a saturated NaHCO_3_ solution (aq 30 mL), dried over MgSO_4_, filtered, and the solvent was removed under reduced pressure. The crude product was purified by column chromatography (cyclohexane/EtOAc 90:10 → 50:50 over 30 min) to afford **86** (2.00 g, 6.00 mmol, 99%) as a white solid. mp 150.2 °C; ^1^H NMR (500 MHz, DMSO-*d*_6_, 300 K): δ (ppm) 10.19 (s, 1H), 7.99 (d, ^4^*J* = 2.3 Hz, 1H), 7.54 (d, ^3^*J* = 8.9 Hz, 1H), 7.46 (dd, ^3^*J* = 8.9 Hz, ^4^*J* = 2.3 Hz, 1H), 6.85 (br s, 1H), 3.21 (dt, ^3^*J* = 7.2 Hz, ^3^*J* = 6.0 Hz, 1H), 2.47 (t, ^3^*J* = 7.2 Hz, 1H), 1.37 (s, 9H); ^13^C NMR (125 MHz, DMSO-*d*_6_, 300 K): δ (ppm) 169.9, 155.5, 139.2, 130.8, 130.5, 124.4, 120.3, 119.1, 77.6, 36.8, 36.3, 28.2; HR-MS: calcd for C_14_H_18_Cl_2_N_2_O_3_Na [M + Na]^+^, 355.0598; found, 355.0592.

#### 3-Amino-*N*-(3,4-dichlorophenyl)propanamide (**87**)

**86** (1.00 g, 3.00 mmol, 1.00 equiv) was suspended in DCM (20 mL) and TFA (3.42 g, 30.00 mmol, 10 equiv, 2.31 mL) was added, and the reaction mixture was stirred intensively at room temperature until TLC indicated complete conversion (2.5 h). The solvent was removed under reduced pressure and the residue was taken up in EtOAc (150 mL). The organic layer was washed with a saturated NaHCO_3_ solution (aq) until the aqueous layer remained slightly basic. Subsequently, the organic layer was washed with a saturated NaCl solution (aq 25 mL), dried over MgSO_4_, and filtered. After removal of the solvent under reduced pressure, **87** (629 mg, 2.70 mmol, 90%) was isolated as a yellowish oil that crystallized upon standing. mp 141.5 °C; ^1^H NMR (500 MHz, DMSO-*d*_6_, 300 K): δ (ppm) 10.25 (br s, 1H), 8.01 (d, ^4^*J* = 2.3 Hz, 1H), 7.56 (d, ^3^*J* = 8.9 Hz, 1H), 7.48 (dd, ^3^*J* = 8.7 Hz, ^4^*J* = 2.3 Hz, 1H), 6.66 (br s, 2H), 3.02 (t, ^3^*J* = 6.8 Hz, 1H), 2.63 (t, ^3^*J* = 6.8 Hz, 2H); ^13^C NMR (125 MHz, DMSO-*d*_6_, 300 K): δ (ppm) 169.4, 139.0, 130.9, 130.6, 124.6, 120.3, 119.1, 35.6, 35.2; HR-MS: calcd for C_9_H_10_Cl_2_N_2_OH [M + H]^+^, 233.0254; found, 233.0247.

#### 1-Methyl-2*H*-benzo[*d*][1,3]oxazine-2,4(1*H*)-dione (**88**)

In a thoroughly dried N_2_ flask, isatoic anhydride (1.00 g, 6.13 mmol, 1.00 equiv) was dissolved in DMAc (3 mL), then DIPEA (1.59 g, 12.26 mmol, 2.00 equiv, 2.09 mL) was added, and the mixture was stirred for 10 min at room temperature under an argon atmosphere. Subsequently MeI (1.74 g, 12.26 mmol, 2.00 equiv, 0.76 mL) was added over 5 min. The reaction mixture was stirred for 18 h at 45 °C, upon which a white solid precipitated (∼2 h). After cooling to room temperature, 6 mL H_2_O was added, and the suspension was stirred intensively for an additional 20 min. The white solid was filtered off, washed with H_2_O (2 × 5 mL) then with cyclohexane (2 × 5 mL), and subsequently dried in vacuo. **88** (995 mg, 5.62 mmol, 92%) was obtained as a white, finely powdered solid. mp 176.5 °C; ^1^H NMR (500 MHz, DMSO-*d*_6_, 300 K): δ (ppm) 8.01 (ddd, ^3^*J* = 7.7 Hz, ^4^*J* = 1.7 Hz, ^5^*J* = 0.6 Hz, 1H), 7.86 (ddd, ^3^*J* = 8.3 Hz, ^3^*J* = 7.5 Hz, ^4^*J* = 1.7 Hz, 1H), 7.44 (d, ^3^*J* = 8.3 Hz, 1H), 7.34 (ddd, ^3^*J* = 7.7 Hz, ^3^*J* = 7.5 Hz, ^4^*J* = 0.9 Hz, 1H), 3.47 (s, 3H); ^13^C NMR (125 MHz, DMSO-*d*_6_, 300 K): δ (ppm) 158.9, 147.7, 142.2, 137.1, 129.2, 123.5, 114.8, 111.5, 31.6; HR-MS: calcd for C_9_H_7_NO_3_H [M + H]^+^, 178.0499; found, 178.0494.

#### *N*-(3,4-Dichlorophenyl)-2-(methylamino)benzamide (**89**)

**88** (800 mg, 4.51 mmol, 1.00 equiv) was dissolved in glacial acetic acid (15 mL) and 3,4-dichloroaniline (2192 mg, 13.53 mmol, 3.00 equiv) dissolved in glacial acetic acid (15 mL) was added slowly. The reaction mixture was stirred for 4 h at 120 °C. After cooling to room temperature, the mixture was poured into H_2_O (60 mL) and the aqueous layer was extracted with EtOAc (3 × 40 mL). The combined organic layers were washed with a saturated NaHCO_3_ solution (aq 2 × 25 mL), dried over MgSO_4_, filtered, and the solvent was removed under reduced pressure. The remaining residue was recrystallized from a H_2_O/ethanol (1:1) mixture. The precipitated purple solid was filtered off, washed with H_2_O, and finally dried in vacuo, yielding **89** (938 mg, 3.18 mmol, 71%) as fine, needle-shaped, purple crystals. mp 151.7 °C; ^1^H NMR (500 MHz, DMSO-*d*_6_, 300 K): δ (ppm) 10.28 (s, 1H), 8.12 (d, ^4^*J* = 2.6 Hz, 1H), 7.73–7.65 (m, 2H), 7.58 (d, ^3^*J* = 8.9 Hz, 1H), 7.37 (ddd, ^3^*J* = 8.3 Hz, ^3^*J* = 7.2 Hz, ^4^*J* = 1.4 Hz, 1H), 7.29 (d, ^3^*J* = 5.4 Hz, 1H), 6.70 (dd, ^3^*J* = 8.5 Hz, ^4^*J* = 0.7 Hz, 1H), 6.64 (ddd, ^3^*J* = 7.7 Hz, ^3^*J* = 7.2 Hz, ^4^*J* = 1.2 Hz, 1H), 2.80 (d, ^3^*J* = 5.2 Hz, 3H); ^13^C NMR (125 MHz, DMSO-*d*_6_, 300 K): δ (ppm) 168.2, 150.1, 139.3, 133.1, 130.7, 130.3, 128.9, 124.7, 121.5, 120.3, 114.8, 114.0, 110.7, 29.3; HR-MS: calcd for C_14_H_12_Cl_2_N_2_ONa [M + Na]^+^, 317.0219; found, 317.0214; Elemental analysis calcd (%) for C_14_H_12_Cl_2_N_2_O: C, 56.97; H, 4.10; N, 9.49. Found: C, 56.81; H, 4.01; N, 9.44.

#### 3-[(3-Nitrophenyl)sulfonamido]thiophene-2-carboxylic Acid (**90**)

**25** (650 mg, 1.90 mmol, 1.00 equiv) was suspended in EtOH (10 mL) and NaOH (380 mg, 9.50 mmol, 5.00 equiv) was added. The reaction mixture was stirred for 18 h at 80 °C. The solvent was removed under reduced pressure, the oily residue taken up in 1 M HCl (aq 15 mL), and stirred vigorously for 20 min, forming a light-brown solid, which was filtered off and washed with H_2_O (5 × 5 mL). **90** (554 mg, 1.69 mmol, 89%) was obtained after drying in vacuo. mp 179.5 °C; ^1^H NMR (500 MHz, DMSO-*d*_6_, 300 K): δ (ppm) 10.23 (br s, 1H), 8.60 (dd, ^4^*J* = 2.0 Hz, 1H), 8.48 (ddd, ^3^*J* = 8.0 Hz, ^4^*J* = 2.0 Hz, ^4^*J* = 1.2 Hz, 1H), 8.27 (ddd, ^3^*J* = 8.0 Hz, ^4^*J* = 1.7 Hz, ^4^*J* = 1.2 Hz, 1H), 7.88 (dd, ^3^*J* = 8.0 Hz, 1H), 7.81 (d, ^3^*J* = 5.4 Hz, 1H), 7.19 (d, ^3^*J* = 5.4 Hz, 1H); ^13^C NMR (125 MHz, DMSO-*d*_6_, 300 K): δ (ppm) 163.7, 147.9, 140.7, 140.4, 132.6, 132.5, 131.4, 127.9, 121.9, 121.7, 115.4; HR-MS: calcd for C_11_H_7_N_2_O_6_S_2_ [M – H]^−^ 326.9751; found, 326.9752.

## Supporting Information Available

Molecular formula strings (CSV)
Additional data on the compounds, structural formula, SMILES ID, status of synthesis, purity, screening data, IC_50_ curve, IC_50_ value, IC_50_ 95% confidence interval, Gini selectivity score, ratio of IC_50_ values between SOAT, ASBT, and NTCP, as well as NMR spectra, and cytotoxicity mean graphs of all compounds used (PDF)

## Abbreviations

AcOH: acetic acid
ASBT: apical sodium-dependent bile acid transporter
BA: bile acid
BS: bile salt
DHEAS: dehydroepiandrosterone sulfate
DIPEA: *N*,*N*-diisopropylethylamine
DMAc: *N*,*N*-dimethylacetamide
EDC–HCl: 1-ethyl-3-(3-(dimethylaminopropyl)-carbodiimide-hydrochloride
HBTU: 2-(1*H*-benzotriazol-1-yl)-1,1,3,3-tetramethyluronium hexafluorophosphate
HBV: hepatitis B virus
HDV: hepatitis D virus
HOBt: 1-hydroxybenzotriazole
IC_50_: half-maximal inhibitory concentration
NMM: *N*-methyl morpholine
NTCP: Na^+^/taurocholate cotransporting polypeptide
SOAT: sodium-dependent organic anion transporter
TC: taurocholic acid
TEA: triethylamine
