# Supplementary material for: Structure–Activity Relationships and Target Selectivity of Phenylsulfonylamino-Benzanilide Inhibitors Based on S1647 at the SLC10 Carriers ASBT, NTCP, and SOAT
Source: J Med Chem. 2024 Oct 17;67(21):19342–64. doi: 10.1021/acs.jmedchem.4c01743 (PMC11571210; doi:10.1021/acs.jmedchem.4c01743)

Supporting Information

Structure-Activity Relationships and Target Selectivity of Phenylsulfonylamino-Benzanilide Inhibitors Based on S1647 at the SLC10 Carriers ASBT, NTCP, and SOAT

Marie Wannowius[†], Christopher Neelen[†], Philipp Lotz[‡], Michael Daude[‡], Anita Neubauer[†], Bärbel Fühler[†], Wibke E. Diederich[‡,*], Joachim Geyer[†,*]

[†]Institute of Pharmacology and Toxicology, Faculty of Veterinary Medicine, Biomedical Research Center Seltersberg (BFS), Justus Liebig University of Giessen, Schubertstr. 81, 35392 Giessen, Germany

[‡]Department of Medicinal Chemistry and Core Facility Medicinal Chemistry, Center for Tumor- and Immune Biology, Philipps University Marburg, Hans-Meerwein-Str. 3, 35043 Marburg, Germany

*Shared last authorship

Correspondence

Prof. Dr. Joachim Geyer, Institute of Pharmacology and Toxicology, Biomedical Research Center Seltersberg (BFS), Schubertstr. 81, 35392 Giessen, Germany

Phone: +49 641 99 38404

E-Mail: Joachim.M.Geyer@vetmed.uni-giessen.de

Content

Structures of all compounds 3

SMILES and status of synthesis 6

IC_{50} – 95% confidence intervals 9

IC_{50} – Curves 11

IC_{50} – Selectivity 14

Screening Data (no IC_{50}) 16

Compound - order information 17

NMR Data 20

N-(3,4-Dichlorophenyl)-2-[(3-nitrophenyl)sulfonamido]benzamide (1) 20

N-(3,4-dichlorophenyl)-2-[(2-nitrophenyl)sulfonamido]benzamide (3) 21

2-[(3-Cyanophenyl)sulfonamido)-*N*-(3,4-dichlorophenyl)benzamide (4) 22

2-{[3-(1*H*-Tetrazol-5-yl)phenyl]sulfonamido}-*N*-(3,4-dichlorophenyl)benzamide (5) 23

3-(*N*-{2-[(3,4-dichlorophenyl)carbamoyl]phenyl}sulfamoyl)benzoic acid (6) 24

2-(Benzo[*c*][1,2,5]oxadiazole-4-sulfonamido)-*N*-(3,4-dichlorophenyl)benzamide (7) 26

2-[(4-Chloro-3-nitrophenyl)sulfonamido]-*N*-(3,4-dichlorophenyl)benzamide (11) 27

5-Chloro-*N*-(3,4-dichlorophenyl)-2-[(3-nitrophenyl)sulfonamido]benzamide (12) 28

5-Chloro-2-[(4-chloro-3-nitrophenyl)sulfonamido]-*N*-(3,4-dichlorphenyl)benzamide (13) 29

N-(2,4-Dichlorophenyl)-2-[(3-nitrophenyl)sulfonamido]benzamide (20) 30

N-(3,4-Dichlorophenyl)-3-[(3-nitrophenyl)sulfonamido]propanamide (21) 31

N-(3,4-Dichlorophenyl)-2-[(*N*-methyl-3-nitrophenyl)sulfonamido]benzamide (22) 32

N-(3,4-Dichlorophenyl)-2-(3-nitrobenzamido)benzamide (23) 33

N-(3,4-Dichlorophenyl)-3-[(3-nitrophenyl)sulfonamido]thiophene-2-carboxamide (24) 34

Methyl-2-[(3-nitrophenyl)sulfonamido]benzoate (28) 35

2-[(3-Nitrophenyl)sulfonamido]benzoic acid (76) 36

tert-Butyl-(4-chloro-2-[(3,4-dichlorophenyl)carbamoyl)phenyl]carbamate (83) 37

N-(3,4-Dichlorophenyl)-2-(methylamino)benzamide (89) 38

Cytotoxicity testing 39

Structures of all compounds

Figure S1 | Structures of all compounds used.

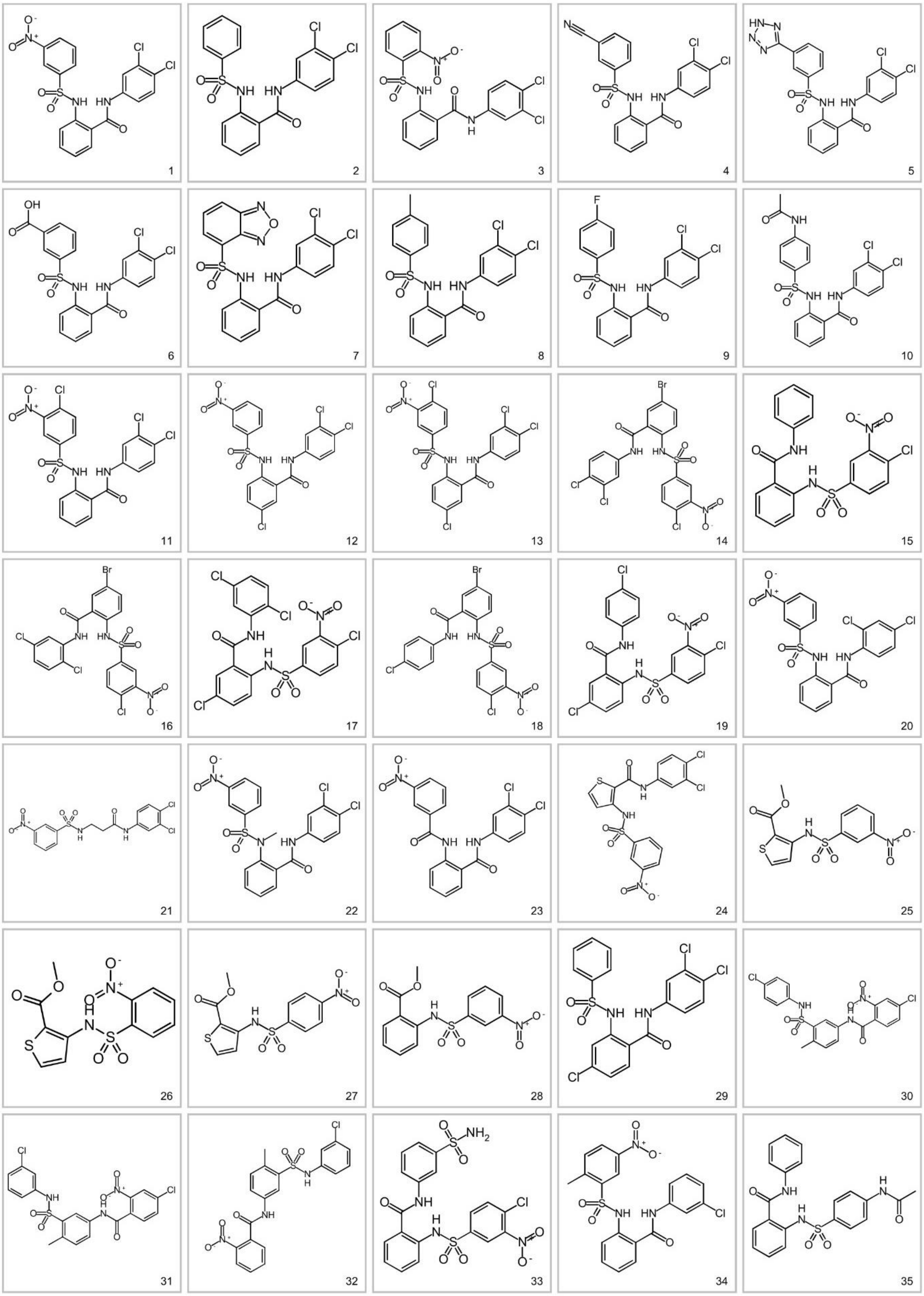

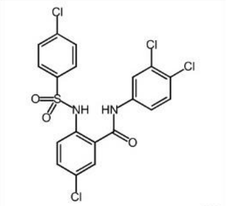
36

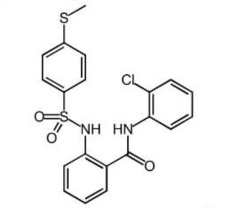
37

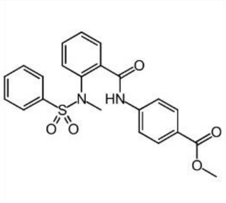
38

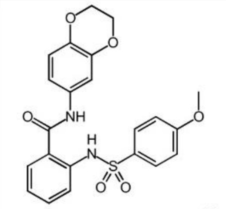
39

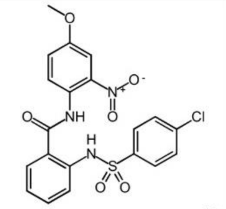
40

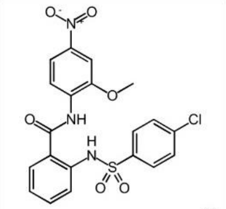
41

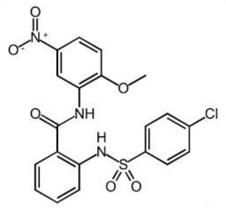
42

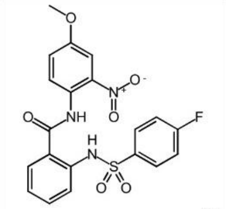
43

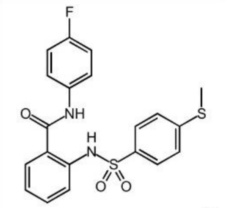
44

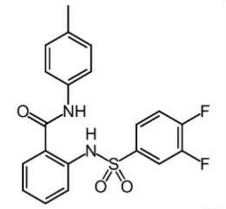
45

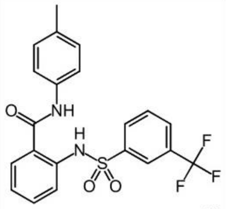
46

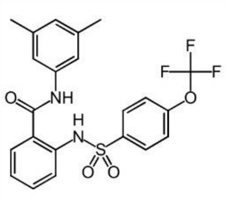
47

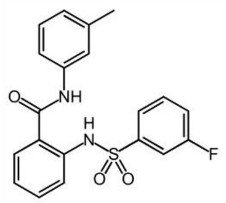
48

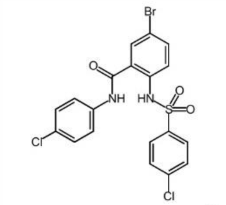
49

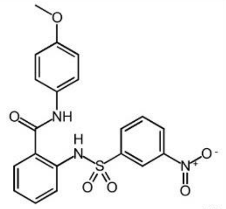
50

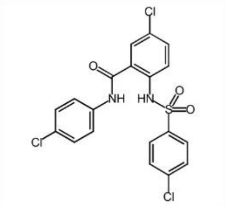
51

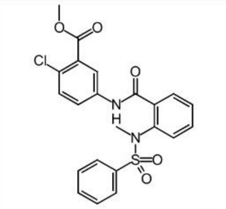
52

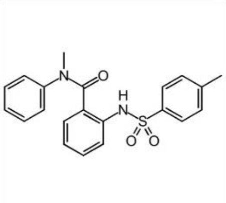
53

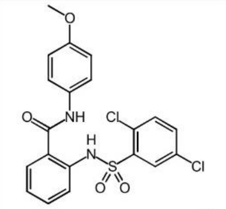
54

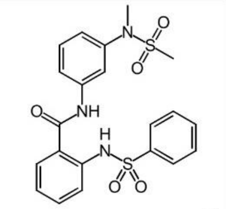
55

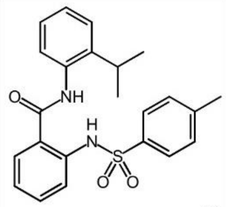
56

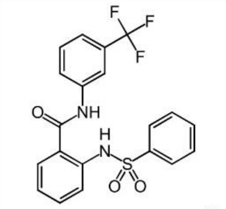
57

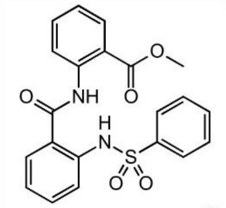
58

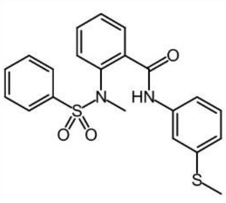
59

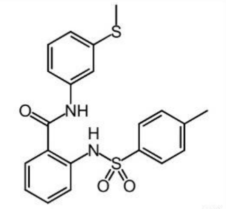
60

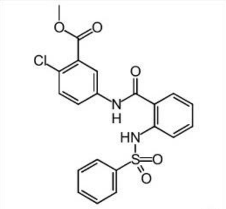
61

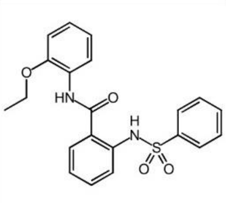
62

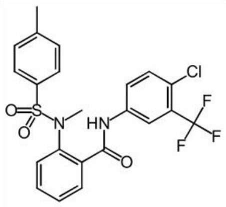
63

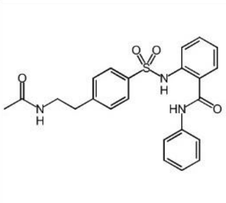
64

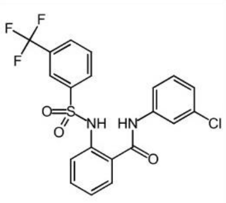
65

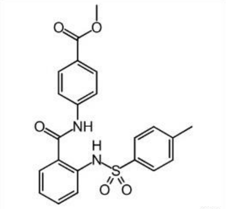
66

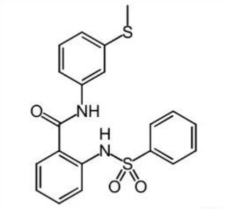
67

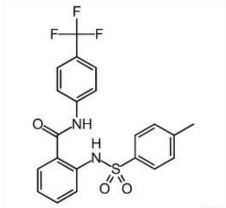
68

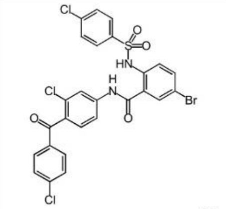
69

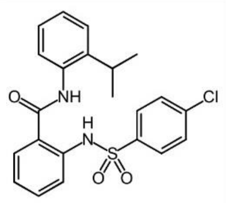
70

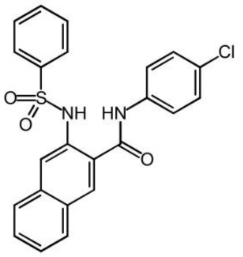
Cl
O
S
NH
HN
O

71

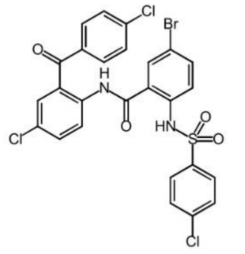
Cl
Br
O
H
N
Cl
O
HN
S
O
O
Cl

72

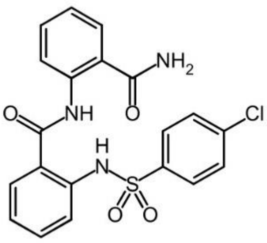
NH2
O
NH
O
Cl
H
N
S
O
O

73

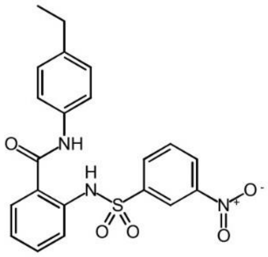
O
NH
H
N
S
O
O
N+
O-
O

74

SMILES and status of synthesis

Table S1 | Compounds and their SMILES ID and synthesis status.

compound	SMILES	status of synthesis
**1**	[O-][N+](c1cccc(S(Nc(cccc2)c2C(Nc(cc2)cc(Cl)c2Cl)=O)(=O)=O)c1)=O	self-synthesized
**2**	O=C(c(cccc1)c1NS(c1ccccc1)(=O)=O)Nc(cc1)cc(Cl)c1Cl	ordered at MolPort
**3**	[O-][N+](c(cccc1)c1S(Nc(cccc1)c1C(Nc(cc1)cc(Cl)c1Cl)=O)(=O)=O)=O	self-synthesized
**4**	N#Cc1cccc(S(Nc(cccc2)c2C(Nc(cc2)cc(Cl)c2Cl)=O)(=O)=O)c1	self-synthesized
**5**	O=C(c(cccc1)c1NS(c1cc(-c2n[nH]nn2)ccc1)(=O)=O)Nc(cc1)cc(Cl)c1Cl	self-synthesized
**6**	OC(c1cccc(S(Nc(cccc2)c2C(Nc(cc2)cc(Cl)c2Cl)=O)(=O)=O)c1)=O	self-synthesized
**7**	O=C(c(cccc1)c1NS(c1cccc2nonc12)(=O)=O)Nc(cc1)cc(Cl)c1Cl	self-synthesized
**8**	Cc(cc1)ccc1S(Nc(cccc1)c1C(Nc(cc1)cc(Cl)c1Cl)=O)(=O)=O	ordered at MolPort
**9**	O=C(c(cccc1)c1NS(c(cc1)ccc1F)(=O)=O)Nc(cc1)cc(Cl)c1Cl	ordered at MolPort
**10**	CC(Nc(cc1)ccc1S(Nc(cccc1)c1C(Nc(cc1)cc(Cl)c1Cl)=O)(=O)=O)=O	ordered at MolPort
**11**	[O-][N+](c(cc(cc1)S(Nc(cccc2)c2C(Nc(cc2)cc(Cl)c2Cl)=O)(=O)=O)c1Cl)=O	self-synthesized
**12**	[O-][N+](c1cccc(S(Nc(ccc(Cl)c2)c2C(Nc(cc2)cc(Cl)c2Cl)=O)(=O)=O)c1)=O	self-synthesized
**13**	[O-][N+](c(cc(cc1)S(Nc(ccc(Cl)c2)c2C(Nc(cc2)cc(Cl)c2Cl)=O)(=O)=O)c1Cl)=O	self-synthesized
**14**	[O-][N+](c(cc(cc1)S(Nc(ccc(Br)c2)c2C(Nc(cc2)cc(Cl)c2Cl)=O)(=O)=O)c1Cl)=O	ordered at MolPort
**15**	[O-][N+](c(cc(cc1)S(Nc(cccc2)c2C(Nc2ccccc2)=O)(=O)=O)c1Cl)=O	ordered at MolPort
**16**	[O-][N+](=O)c1cc(ccc1Cl)S(=O)(=O)Nc1ccc(Br)cc1C(=O)Nc1cc(Cl)ccc1Cl	ordered at MolPort
**17**	[O-][N+](c(cc(cc1)S(Nc(ccc(Cl)c2)c2C(Nc(cc(cc2)Cl)c2Cl)=O)(=O)=O)c1Cl)=O	ordered at MolPort
**18**	[O-][N+](=O)c1cc(ccc1Cl)S(=O)(=O)Nc1ccc(Br)cc1C(=O)Nc1ccc(Cl)cc1	ordered at MolPort
**19**	[O-][N+](c(cc(cc1)S(Nc(ccc(Cl)c2)c2C(Nc(cc2)ccc2Cl)=O)(=O)=O)c1Cl)=O	ordered at MolPort
**20**	[O-][N+](c1cccc(S(Nc(cccc2)c2C(Nc(ccc(Cl)c2)c2Cl)=O)(=O)=O)c1)=O	self-synthesized
**21**	[O-][N+](c1cccc(S(NCCC(Nc(cc2)cc(Cl)c2Cl)=O)(=O)=O)c1)=O	self-synthesized
**22**	CN(c(cccc1)c1C(Nc(cc1)cc(Cl)c1Cl)=O)S(c1cc([N+]([O-])=O)ccc1)(=O)=O	self-synthesized
**23**	[O-][N+](c1cccc(C(Nc(cccc2)c2C(Nc(cc2)cc(Cl)c2Cl)=O)=O)c1)=O	self-synthesized

**24**	[O-][N+](c1cccc(S(Nc2c(C(Nc(cc3)cc(Cl)c3Cl)=O)scc2)(=O)=O)c1)=O	self-synthesized
**25**	COC(c(scc1)c1NS(c1cc([N+]([O-])=O)ccc1)(=O)=O)=O	self-synthesized
**26**	COC(=O)c1sccc1NS(=O)(=O)c1ccccc1[N+]([O-])=O	ordered at MolPort
**27**	COC(=O)c1sccc1NS(=O)(=O)c1ccc(cc1)[N+]([O-])=O	ordered at MolPort
**28**	COC(c(cccc1)c1NS(c1cc([N+]([O-])=O)ccc1)(=O)=O)=O	self-synthesized
**29**	O=C(c(ccc(Cl)c1)c1NS(c1ccccc1)(=O)=O)Nc(cc1)cc(Cl)c1Cl	ordered at MolPort
**30**	Cc(ccc(NC(c(ccc(Cl)c1)c1[N+]([O-])=O)=O)c1)c1S(Nc(cc1)ccc1Cl)(=O)=O	ordered at MolPort
**31**	Cc(ccc(NC(c(ccc(Cl)c1)c1[N+]([O-])=O)=O)c1)c1S(Nc1cc(Cl)ccc1)(=O)=O	ordered at MolPort
**32**	Cc(ccc(NC(c(cccc1)c1[N+]([O-])=O)=O)c1)c1S(Nc1cccc(Cl)c1)(=O)=O	ordered at MolPort
**33**	NS(c1cccc(NC(c(cccc2)c2NS(c(cc2)cc([N+]([O-])=O)c2Cl)(=O)=O)=O)c1)(=O)=O	ordered at MolPort
**34**	Cc(ccc([N+]([O-])=O)c1)c1S(Nc(cccc1)c1C(Nc1cccc(Cl)c1)=O)(=O)=O	ordered at MolPort
**35**	CC(=O)Nc1ccc(cc1)S(=O)(=O)Nc1ccccc1C(=O)Nc1ccccc1	ordered at MolPort
**36**	Clc1ccc(cc1)S(=O)(=O)Nc1ccc(Cl)cc1C(=O)Nc1ccc(Cl)c(Cl)c1	ordered at MolPort
**37**	CSc1ccc(cc1)S(=O)(=O)Nc1ccccc1C(=O)Nc1ccccc1Cl	ordered at MolPort
**38**	COC(=O)c1ccc(NC(=O)c2ccccc2N(C)S(=O)(=O)c2ccccc2)cc1	ordered at MolPort
**39**	COc1ccc(cc1)S(=O)(=O)Nc1ccccc1C(=O)Nc1ccc2OCCOc2c1	ordered at MolPort
**40**	COc1ccc(NC(=O)c2ccccc2NS(=O)(=O)c2ccc(Cl)cc2)c(c1)[N+]([O-])=O	ordered at MolPort
**41**	COc1cc(ccc1NC(=O)c1ccccc1NS(=O)(=O)c1ccc(Cl)cc1)[N+]([O-])=O	ordered at MolPort
**42**	COc1ccc(cc1NC(=O)c1ccccc1NS(=O)(=O)c1ccc(Cl)cc1)[N+]([O-])=O	ordered at MolPort
**43**	COc1ccc(NC(=O)c2ccccc2NS(=O)(=O)c2ccc(F)cc2)c(c1)[N+]([O-])=O	ordered at MolPort
**44**	CSc1ccc(cc1)S(=O)(=O)Nc1ccccc1C(=O)Nc1ccc(F)cc1	ordered at MolPort
**45**	Cc1ccc(NC(=O)c2ccccc2NS(=O)(=O)c2ccc(F)c(F)c2)cc1	ordered at MolPort
**46**	Cc1ccc(NC(=O)c2ccccc2NS(=O)(=O)c2cccc(c2)C(F)(F)F)cc1	ordered at MolPort
**47**	Cc1cc(C)cc(NC(=O)c2ccccc2NS(=O)(=O)c2ccc(OC(F)(F)F)cc2)c1	ordered at MolPort
**48**	Cc1cccc(NC(=O)c2ccccc2NS(=O)(=O)c2cccc(F)c2)c1	ordered at MolPort
**49**	Clc1ccc(NC(=O)c2cc(Br)ccc2NS(=O)(=O)c2ccc(Cl)cc2)cc1	ordered at MolPort
**50**	COc1ccc(NC(=O)c2ccccc2NS(=O)(=O)c2cccc(c2)[N+]([O-])=O)cc1	ordered at MolPort

**51**	Clc1ccc(NC(=O)c2cc(Cl)ccc2NS(=O)(=O)c2ccc(Cl)cc2)cc1	ordered at MolPort
**52**	COC(=O)c1cc(NC(=O)c2ccccc2N(C)S(=O)(=O)c2ccccc2)ccc1Cl	ordered at MolPort
**53**	CN(C(=O)c1ccccc1NS(=O)(=O)c1ccc(C)cc1)c1ccccc1	ordered at MolPort
**54**	COc1ccc(NC(=O)c2ccccc2NS(=O)(=O)c2cc(Cl)ccc2Cl)cc1	ordered at MolPort
**55**	CN(c1cccc(NC(=O)c2ccccc2NS(=O)(=O)c2ccccc2)c1)S(C)(=O)=O	ordered at MolPort
**56**	CC(C)c1ccccc1NC(=O)c1ccccc1NS(=O)(=O)c1ccc(C)cc1	ordered at MolPort
**57**	FC(F)(F)c1cccc(NC(=O)c2ccccc2NS(=O)(=O)c2ccccc2)c1	ordered at MolPort
**58**	COC(=O)c1ccccc1NC(=O)c1ccccc1NS(=O)(=O)c1ccccc1	ordered at MolPort
**59**	CSc1cccc(NC(=O)c2ccccc2N(C)S(=O)(=O)c2ccccc2)c1	ordered at MolPort
**60**	CSc1cccc(NC(=O)c2ccccc2NS(=O)(=O)c2ccc(C)cc2)c1	ordered at MolPort
**61**	COC(=O)c1cc(NC(=O)c2ccccc2NS(=O)(=O)c2ccccc2)ccc1Cl	ordered at MolPort
**62**	CCOc1ccccc1NC(=O)c1ccccc1NS(=O)(=O)c1ccccc1	ordered at MolPort
**63**	CN(c1ccccc1C(=O)Nc1ccc(Cl)c(c1)C(F)(F)F)S(=O)(=O)c1ccc(C)cc1	ordered at MolPort
**64**	CC(=O)NCCc1ccc(cc1)S(=O)(=O)Nc1ccccc1C(=O)Nc1ccccc1	ordered at MolPort
**65**	FC(F)(F)c1cccc(c1)S(=O)(=O)Nc1ccccc1C(=O)Nc1cccc(Cl)c1	ordered at MolPort
**66**	COC(=O)c1ccc(NC(=O)c2ccccc2NS(=O)(=O)c2ccc(C)cc2)cc1	ordered at MolPort
**67**	CSc1cccc(NC(=O)c2ccccc2NS(=O)(=O)c2ccccc2)c1	ordered at MolPort
**68**	Cc1ccc(cc1)S(=O)(=O)Nc1ccccc1C(=O)Nc1ccc(cc1)C(F)(F)F	ordered at MolPort
**69**	Clc1ccc(cc1)C(=O)c1ccc(NC(=O)c2cc(Br)ccc2NS(=O)(=O)c2ccc(Cl)cc2)cc1Cl	ordered at MolPort
**70**	CC(C)c1ccccc1NC(=O)c1ccccc1NS(=O)(=O)c1ccc(Cl)cc1	ordered at MolPort
**71**	Clc1ccc(NC(=O)c2cc3ccccc3cc2NS(=O)(=O)c2ccccc2)cc1	ordered at MolPort
**72**	Clc1ccc(cc1)C(=O)c1cc(Cl)ccc1NC(=O)c1cc(Br)ccc1NS(=O)(=O)c1ccc(Cl)cc1	ordered at MolPort
**73**	NC(=O)c1ccccc1NC(=O)c1ccccc1NS(=O)(=O)c1ccc(Cl)cc1	ordered at MolPort
**74**	CCc1ccc(NC(=O)c2ccccc2NS(=O)(=O)c2cccc(c2)[N+]([O-])=O)cc1	ordered at MolPort

IC_{50} – 95% confidence intervals

Table S2 | Details of IC_{50} experiments. Including number of replicates, IC_{50} value, lower and upper 95% IC_{50} confidence intervals | n.i., no inhibition; n.m., not meaningful; Ps, prescreen at 100 µM compound concentration

compound	Details of experiment	IC_{50} [µM] - SOAT (DHEAS)	SOAT (DHEAS): IC_{50} 95% confidence interval from [µM]	SOAT (DHEAS): IC_{50} 95% confidence interval to [µM]	IC_{50} [µM] - ASBT (TC)	ASBT (TC): IC_{50} 95% confidence interval from [µM]	ASBT (TC): IC_{50} 95% confidence interval to [µM]	IC_{50} [µM] - NTCP (TC)	NTCP (TC): IC_{50} 95% confidence interval from [µM]	NTCP (TC): IC_{50} 95% confidence interval to [µM]
**1**	IC_{50} n=2	3.45	2.80	4.24	13.44	11.41	15.83	10.39	8.25	13.08
**2**	IC_{50} n=2	n.i.	n.i.	n.i.	n.i.	n.i.	n.i.	156.20	35.83	680.70
**3**	IC_{50} n=2	25.69	14.11	46.79	n.i.	n.i.	n.i.	n.i.	n.i.	n.i.
**4**	IC_{50} n=2	10.86	6.45	18.27	301.00	39.84	2274.00	99.33	33.36	295.70
**5**	IC_{50} n=2	4.40	3.56	5.45	206.70	110.30	387.50	144.00	98.15	211.10
**6**	IC_{50} n=2	11.15	7.73	16.09	n.i.	n.i.	n.i.	134.20	78.27	230.00
**7**	IC_{50} n=2	34.57	11.54	103.50	693.00	51.52	9322.00	123.70	46.45	329.70
**8**	IC_{50} n=2	n.i.	n.i.	n.i.	n.i.	n.i.	n.i.	504.80	126.70	2012.00
**9**	IC_{50} n=2	138.80	30.34	635.10	476.30	83.21	2726.00	n.i.	n.i.	n.i.
**10**	IC_{50} n=2	14.62	8.31	25.72	94.86	72.70	123.80	40.34	29.87	54.48
**11**	IC_{50} n=2	37.59	9.64	146.50	n.i.	n.i.	n.i.	n.i.	n.i.	n.i.
**12**	IC_{50} n=2	1.93	1.06	3.51	155.00	88.67	271.00	13480.00	n.i.	n.i.
**13**	IC_{50} n=2	3.88	2.71	5.56	23.08	15.01	35.49	14.93	9.59	23.26
**14**	IC_{50} n=2	1.43	1.07	1.91	19.60	15.61	24.60	11.12	8.05	15.35
**15**	IC_{50} n=2	48.53	23.86	98.71	39.94	21.01	75.92	20.80	14.08	30.73
**16**	IC_{50} n=2	1.31	0.97	1.77	22.40	19.13	26.21	8.31	7.42	9.29
**17**	IC_{50} n=2	0.75	0.56	1.02	9.39	6.31	13.97	3.90	3.39	4.48
**18**	IC_{50} n=2	8.26	5.23	13.05	25.32	21.59	29.69	7.93	6.98	9.01
**19**	IC_{50} n=2	0.87	0.66	1.15	10.58	n.m.	n.m.	7.15	5.45	9.39
**20**	IC_{50} n=2	6.36	5.42	7.46	12.47	10.97	14.16	2.65	2.21	3.18
**21**	IC_{50} n=2	n.i.	n.i.	n.i.	n.i.	n.i.	n.i.	n.i.	n.i.	n.i.
**22**	IC_{50} n=2	97.84	50.70	188.80	792.70	0.69	907700.00	n.i.	n.i.	n.i.
**23**	IC_{50} n=2	n.i.	n.i.	n.i.	n.i.	n.i.	n.i.	n.i.	n.i.	n.i.
**24**	IC_{50} n=2	0.56	0.38	0.84	16.28	12.35	21.45	8.16	6.92	9.63

**25**	IC_{50} n=2	10.75	8.31	13.91	n.i.	n.i.	n.i.	148.40	54.70	402.80
**26**	IC_{50} n=2	13.47	10.91	16.62	n.i.	n.i.	n.i.	n.i.	n.i.	n.i.
**27**	IC_{50} n=2	11.96	8.49	16.86	231.1	117.1	455.9	437.2	67.67	2825
**28**	IC_{50} n=2	111.40	73.85	168.20	n.i.	n.i.	n.i.	119.80	n.m.	n.m.
**30**	Ps, IC_{50} n=1	31.55	20.24	49.17	n.i.	n.i.	n.i.	113.50	27.30	472.30
**31**	Ps, IC_{50} n=1	10.56	7.26	15.37	194.50	79.97	472.80	131.70	43.12	402.00
**32**	Ps, IC_{50} n=1	11.46	7.58	17.32	130.60	54.02	315.70	155.30	70.80	340.80
**33**	Ps, IC_{50} n=1	2.40	1.80	3.21	65.27	38.28	111.30	17.73	14.66	21.45
**37**	Ps, IC_{50} n=1	17.89	14.44	22.17	55.85	38.21	81.64	11.09	9.70	12.68
**40**	Ps, IC_{50} n=1	1.59	0.96	2.65	n.i.	n.i.	n.i.	14.25	11.74	17.31
**41**	Ps, IC_{50} n=1	4.94	3.88	6.31	8.58	7.45	9.89	7.65	6.69	8.73
**45**	Ps, IC_{50} n=1	10.48	8.30	13.22	16.37	15.04	17.82	11.38	9.33	13.88
**46**	Ps, IC_{50} n=1	11.48	7.68	17.17	17.88	15.67	20.40	8.92	6.14	12.94
**47**	Ps, IC_{50} n=1	19.86	15.20	25.96	17.07	14.85	19.61	15.98	12.93	19.75
**48**	Ps, IC_{50} n=1	19.96	14.58	27.32	34.02	27.98	41.37	13.76	11.71	16.18
**49**	Ps, IC_{50} n=1	7.11	5.00	10.11	38.59	22.31	66.74	17.67	15.31	20.41
**54**	Ps, IC_{50} n=1	n.i.	n.i.	n.i.	15.88	14.31	17.62	45.60	28.14	73.89
**55**	Ps, IC_{50} n=1	175.70	11.51	2680.00	8.66	7.56	9.91	88.87	72.52	108.90
**56**	Ps, IC_{50} n=1	2.55	1.23	5.30	51.98	38.48	70.21	35.82	28.82	44.51
**57**	Ps, IC_{50} n=1	23.69	13.27	42.31	n.i.	n.i.	n.i.	332.60	99.36	1114.00
**65**	Ps, IC_{50} n=1	6.32	3.09	12.93	179.20	34.55	929.30	12.10	10.22	14.32
**66**	Ps, IC_{50} n=1	44.27	25.05	78.23	62.51	52.00	75.14	55.70	46.78	66.31
**67**	Ps, IC_{50} n=1	13.95	5.13	37.93	20.45	14.62	28.62	28.94	25.28	33.12
**68**	Ps, IC_{50} n=1	n.i.	n.i.	n.i.	39.03	33.40	45.60	13.12	9.13	18.84
**69**	Ps, IC_{50} n=1	35.94	25.39	50.87	664.90	232.80	1899.00	40.65	25.64	64.46
**70**	Ps, IC_{50} n=1	15.03	13.43	16.82	22.87	20.54	25.47	11.38	9.33	13.88
**72**	Ps, IC_{50} n=1	4.04	2.64	6.17	374.70	144.10	974.60	178.20	90.13	352.30
**73**	Ps, IC_{50} n=1	36.64	27.17	49.41	182.20	117.70	282.00	95.81	80.77	113.70
**74**	Ps, IC_{50} n=1	24.67	19.31	31.53	19.32	15.64	23.87	12.41	9.67	15.91

IC_{50} – Curves

Figure S2 | IC_{50} curves of the three SLC10 transporters. The transport activity of a carrier is shown as a function of the compound concentration. All curves include the number of independent IC_{50} measurements given in **Table S2**. Each measurement included quadruplicates. Details are indicated in the *methods* section. Orange: SOAT, blue: ASBT, purple: NTCP, gray shaded area: IC_{50} can only be estimated because the maximum inhibitor concentration used was 100 µM.

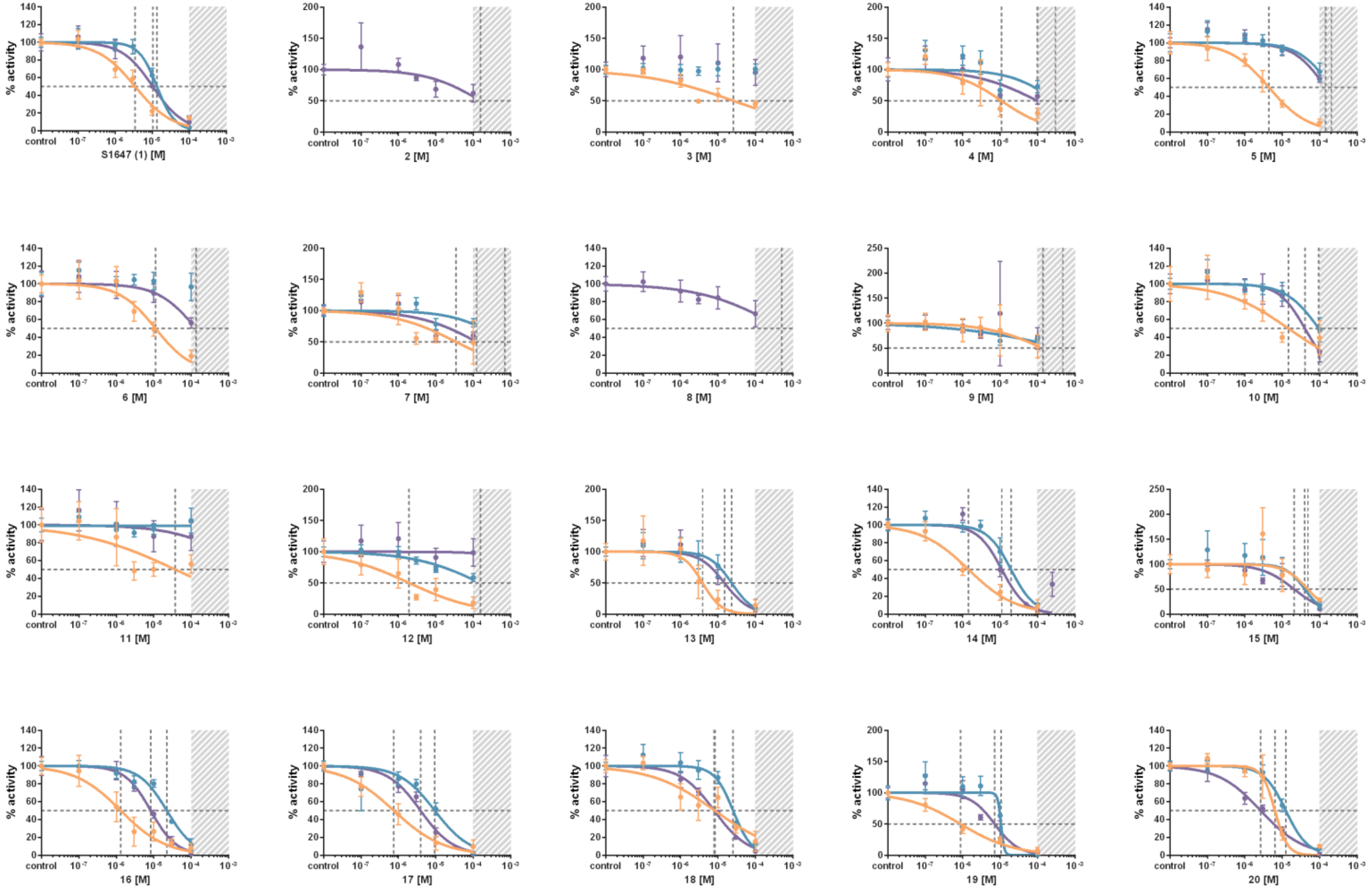

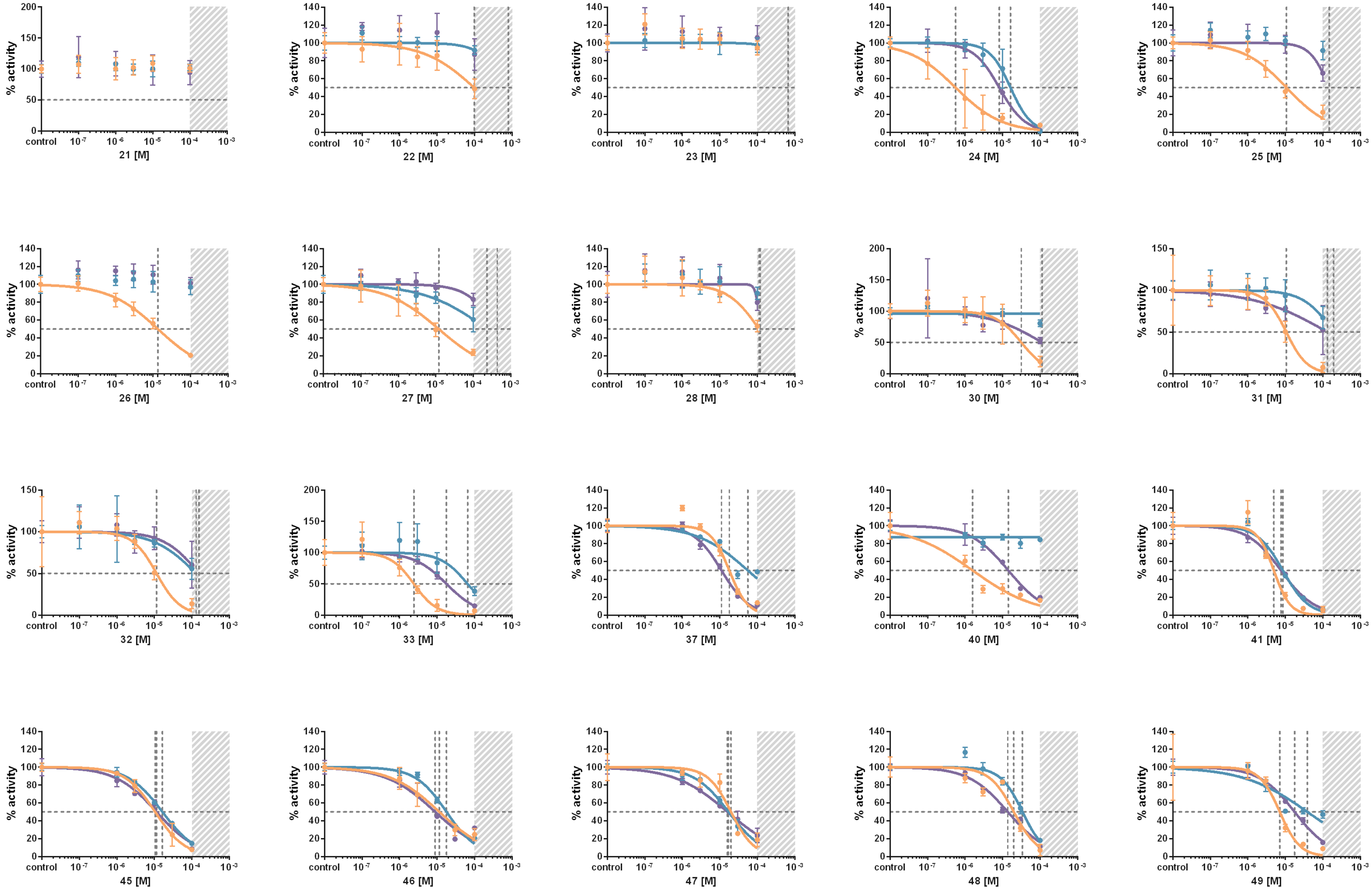
% activity
control
21 [M]
22 [M]
23 [M]
24 [M]
25 [M]
26 [M]
27 [M]
28 [M]
30 [M]
31 [M]
32 [M]
33 [M]
37 [M]
40 [M]
41 [M]
45 [M]
46 [M]
47 [M]
48 [M]
49 [M]

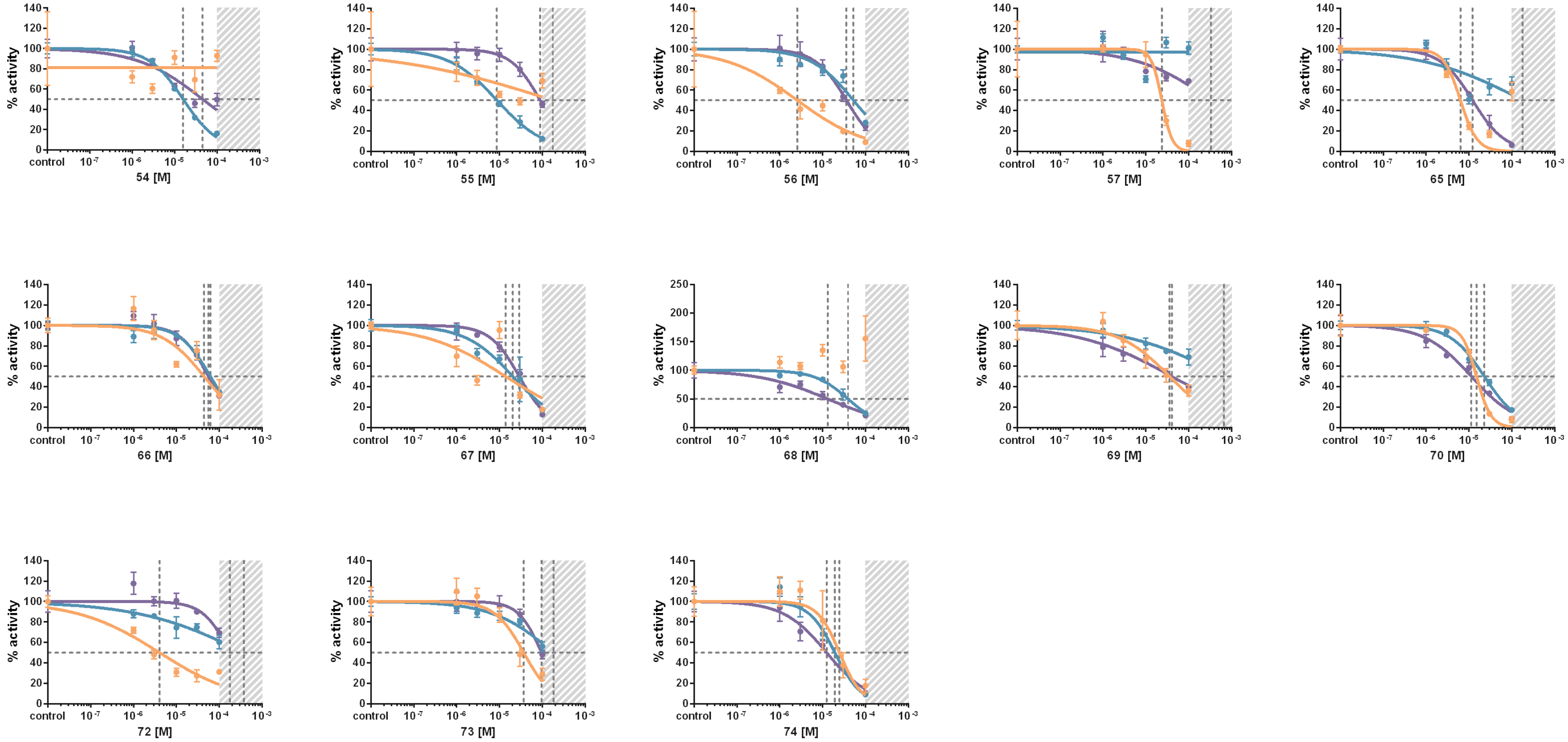
% activity
control
54 [M]
55 [M]
56 [M]
57 [M]
65 [M]
66 [M]
67 [M]
68 [M]
69 [M]
70 [M]
72 [M]
73 [M]
74 [M]

IC_{50} – Selectivity

Table S3 | IC_{50} ratios of the indicated carriers, Gini selectivity score and summary of selectivity status.

compound	ratio IC_{50} SOAT vs. ASBT	ratio IC_{50} SOAT vs. NTCP	ratio IC_{50} ASBT vs. NTCP	Gini selectivity score	summary for selectivity
**1**	3.90	3.01	1.29	0.24413	paninhibitor
**2**	not meaningful	not meaningful	not meaningful	0.26089	no inhibitory potential
**3**	not meaningful	not meaningful	not meaningful	0.32065	does not meet all selectivity criteria
**4**	27.72	9.15	3.03	0.47041	does not meet all selectivity criteria
**5**	46.95	32.70	1.44	0.37979	SOAT selective
**6**	not meaningful	12.04	not meaningful	0.57557	SOAT selective
**7**	20.05	3.58	5.60	0.51565	does not meet all selectivity criteria
**8**	not meaningful	not meaningful	not meaningful	0.1318	no inhibitory potential
**9**	3.43	not meaningful	not meaningful	0.35548	no inhibitory potential
**10**	6.49	2.76	2.35	0.35705	does not meet all selectivity criteria
**11**	not meaningful	not meaningful	not meaningful	0.31489	does not meet all selectivity criteria
**12**	80.35	6988.08	0.01	0.57513	SOAT selective
**13**	5.94	3.84	1.55	0.30549	does not meet all selectivity criteria
**14**	13.67	7.75	1.76	0.37665	does not meet all selectivity criteria
**15**	0.82	0.43	1.92	0.16918	does not meet all selectivity criteria
**16**	17.05	6.32	2.70	0.43903	does not meet all selectivity criteria
**17**	12.47	5.18	2.41	0.41009	paninhibitor
**18**	3.07	0.96	3.19	0.27931	does not meet all selectivity criteria
**19**	12.11	8.18	1.48	0.34775	paninhibitor
**20**	1.96	0.42	4.70	0.30463	paninhibitor
**21**	not meaningful	not meaningful	not meaningful	0	no inhibitory potential
**22**	8.10	not meaningful	not meaningful	0.31813	does not meet all selectivity criteria
**23**	not meaningful	not meaningful	not meaningful	0	no inhibitory potential
**24**	29.05	14.56	1.99	0.41917	SOAT selective
**25**	not meaningful	13.80	not meaningful	0.56895	SOAT selective
**26**	not meaningful	not meaningful	not meaningful	0.32664	SOAT selective
**27**	19.32	36.56	0.53	0.41674	SOAT selective
**28**	not meaningful	1.08	not meaningful	0.48116	no inhibitory potential
**30**	not meaningful	3.60	not meaningful	0.56385	does not meet all selectivity criteria

**31**	18.42	12.47	1.48	0.36414	SOAT selective
**32**	11.40	13.55	0.84	0.32248	SOAT selective
**33**	27.18	7.38	3.68	0.49077	does not meet all selectivity criteria
**37**	3.12	0.62	5.04	0.35176	does not meet all selectivity criteria
**40**	not meaningful	8.95	not meaningful	0.65523	SOAT&NTCP selective
**41**	1.74	1.55	1.12	0.11458	paninhibitor
**45**	1.56	1.09	1.44	0.10271	does not meet all selectivity criteria
**46**	1.56	0.78	2.00	0.15609	does not meet all selectivity criteria
**47**	0.86	0.80	1.07	0.048888	does not meet all selectivity criteria
**48**	1.70	0.69	2.47	0.19939	does not meet all selectivity criteria
**49**	5.43	2.48	2.18	0.33115	does not meet all selectivity criteria
**54**	not meaningful	not meaningful	0.35	0.61808	potentially selective for ASBT & NTCP
**55**	0.05	0.51	0.10	0.40759	ASBT selective
**56**	20.39	14.05	1.45	0.36474	SOAT selective
**57**	not meaningful	14.04	not meaningful	0.47989	does not meet all selectivity criteria
**65**	28.35	1.91	14.81	0.58321	SOAT&NTCP selective
**66**	1.41	1.26	1.12	0.07484	does not meet all selectivity criteria
**67**	1.47	2.07	0.71	0.15777	does not meet all selectivity criteria
**68**	not meaningful	not meaningful	2.97	0.62531	potentially selective for ASBT & NTCP
**69**	18.50	1.13	16.36	0.56549	does not meet all selectivity criteria
**70**	1.52	0.76	2.01	0.15544	does not meet all selectivity criteria
**72**	92.82	44.14	2.10	0.44369	SOAT selective
**73**	4.97	2.61	1.90	0.30841	does not meet all selectivity criteria
**74**	0.78	0.50	1.56	0.14492	does not meet all selectivity criteria

**Legend**	
**ASBT selective**	The IC_{50} value was below 15 µM for ASBT and more than one order of magnitude higher for the other two carriers. Additionally, the IC_{50} of the other two carriers was required to be above 15 µM.
**does not meet all selectivity criteria**	The compound does not meet all selectivity and potency criteria.
**no inhibitory potential**	None of the transporters reaches an IC_{50} below 100 µM with inhibitor X.
**paninhibitor**	All transporters achieve an IC_{50} of less than 15 µM.
**potentially selective for ASBT & NTCP**	The IC_{50} value for ASBT or for NTCP was below 15 µM but still below 50 µM. In addition, SOAT was not inhibited by this compound.
**SOAT selective**	The IC_{50} value was below 15 µM for SOAT and more than one order of magnitude higher for the other two carriers. Additionally, the IC_{50} of the other two carriers was required to be above 15 µM.
**SOAT&NTCP selective**	The IC_{50} value was below 15 µM for SOAT and for NTCP as well as more than one order of magnitude higher for ASBT. Additionally, the IC_{50} of ASBT was required to be above 15 µM.

Screening Data (no IC_{50})

Table S4 | Screening data for compounds that were inactive at a concentration of 100 µM (≥30 % transport activity on all three carriers) and were therefore not selected for IC_{50} determination.

compound	% transporter activity for SOAT at 100 µM compound concentration	% transporter activity for ASBT at 100 µM compound concentration	% transporter activity for NTCP at 100 µM compound concentration
**29**	30.37	89.57	81.62
**34**	91.11	69.76	78.86
**35**	73.64	97.79	66.84
**36**	53.98	87.71	88.64
**38**	92.47	87.37	95.26
**39**	40.17	98.38	116.37
**42**	82.89	104.56	102.22
**43**	64.52	86.80	94.96
**44**	63.71	41.43	49.71
**50**	31.89	35.37	39.56
**51**	33.33	86.23	76.01
**52**	69.13	90.15	72.99
**53**	32.31	81.85	61.89
**58**	86.88	100.69	99.32
**59**	70.39	111.08	98.64
**60**	54.39	42.23	97.24
**61**	58.58	36.48	32.55
**62**	61.25	63.47	36.65
**63**	35.83	100.36	101.07
**64**	91.61	113.91	93.26
**71**	55.27	44.00	51.12

Compound - order information

Table S5 | Purity data and supplier details for the ordered compounds.

Compound	MolPort ID	Supplier	Catalogue number	SMILES	Compound State	Purity
**2**	MolPort-002-299-270	Vitas-M Laboratory, Ltd.	STL072011	Clc1ccc(NC(=O)c2ccccc2NS(=O)(=O)c2ccccc2)cc1Cl	n/a	≥90
**8**	MolPort-002-575-510	Vitas-M Laboratory, Ltd.	STK711372	Cc1ccc(cc1)S(=O)(=O)Nc1ccccc1C(=O)Nc1ccc(Cl)c(Cl)c1	n/a	≥90
**9**	MolPort-005-902-750	ENAMINE Ltd.	Z56566913	Fc1ccc(cc1)S(=O)(=O)Nc1ccccc1C(=O)Nc1ccc(Cl)c(Cl)c1	solid	≥90
**10**	MolPort-020-093-135	ENAMINE Ltd.	Z1216674395	CC(=O)Nc1ccc(cc1)S(=O)(=O)Nc1ccccc1C(=O)Nc1ccc(Cl)c(Cl)c1	solid	≥90
**14**	MolPort-000-748-886	Vitas-M Laboratory, Ltd.	STK017068	[O-][N+](=O)c1cc(ccc1Cl)S(=O)(=O)Nc1ccc(Br)cc1C(=O)Nc1ccc(Cl)c(Cl)c1	n/a	≥90
**15**	MolPort-003-208-758	ENAMINE Ltd.	Z45580419	[O-][N+](=O)c1cc(ccc1Cl)S(=O)(=O)Nc1ccccc1C(=O)Nc1ccccc1	solid	≥90
**16**	MolPort-000-729-425	Vitas M Chemical Limited	STK095310	[O-][N+](=O)c1cc(ccc1Cl)S(=O)(=O)Nc1ccc(Br)cc1C(=O)Nc1cc(Cl)ccc1Cl	n/a	≥90
**17**	MolPort-001-924-570	ChemBridge Corporation	5175529	[O-][N+](=O)c1cc(ccc1Cl)S(=O)(=O)Nc1ccc(Cl)cc1C(=O)Nc1cc(Cl)ccc1Cl	solid	≥90
**18**	MolPort-000-724-152	Vitas M Chemical Limited	STK525903	[O-][N+](=O)c1cc(ccc1Cl)S(=O)(=O)Nc1ccc(Br)cc1C(=O)Nc1ccc(Cl)cc1	n/a	≥90
**19**	MolPort-000-703-374	ChemBridge Corporation	5175347	[O-][N+](=O)c1cc(ccc1Cl)S(=O)(=O)Nc1ccc(Cl)cc1C(=O)Nc1ccc(Cl)cc1	solid	≥90
**26**	MolPort-001-543-190	Vitas M Chemical Limited	STK419526	COC(=O)c1sccc1NS(=O)(=O)c1ccccc1[N+]([O-])=O	n/a	≥90
**27**	MolPort-001-502-887	Vitas M Chemical Limited	STK442735	COC(=O)c1sccc1NS(=O)(=O)c1ccc(cc1)[N+]([O-])=O	n/a	≥90
**29**	MolPort-002-544-987	InterBioScreen Ltd.	STOCK1S-31139	Clc1ccc(C(=O)Nc2ccc(Cl)c(Cl)c2)c(NS(=O)(=O)c2ccccc2)c1	n/a	≥92
**30**	MolPort-010-005-573	ENAMINE Ltd.	Z26480760	Cc1ccc(NC(=O)c2ccc(Cl)cc2[N+]([O-])=O)cc1S(=O)(=O)Nc1ccc(Cl)cc1	oil	≥90
**31**	MolPort-010-005-995	ENAMINE Ltd.	Z26495235	Cc1ccc(NC(=O)c2ccc(Cl)cc2[N+]([O-])=O)cc1S(=O)(=O)Nc1cccc(Cl)c1	solid	≥90
**32**	MolPort-010-005-987	ENAMINE Ltd.	Z26495191	Cc1ccc(NC(=O)c2ccccc2[N+]([O-])=O)cc1S(=O)(=O)Nc1cccc(Cl)c1	solid	≥90
**33**	MolPort-005-894-679	UkrOrgSynthesis Ltd. Stock	PB56920896	NS(=O)(=O)c1cccc(NC(=O)c2ccccc2NS(=O)(=O)c2ccc(Cl)c(c2)[N+]([O-])=O)c1	n/a	≥90
**34**	MolPort-002-232-265	ChemBridge Corporation	7003426	Cc1ccc(cc1S(=O)(=O)Nc1ccccc1C(=O)Nc1cccc(Cl)c1)[N+]([O-])=O	solid	≥90
**35**	MolPort-001-026-945	Specs	AG-690/11821942	CC(=O)Nc1ccc(cc1)S(=O)(=O)Nc1ccccc1C(=O)Nc1ccccc1	n/a	≥90
**36**	MolPort-002-544-895	Specs	AH-034/32474061	Clc1ccc(cc1)S(=O)(=O)Nc1ccc(Cl)cc1C(=O)Nc1ccc(Cl)c(Cl)c1	n/a	≥90
**37**	MolPort-002-276-882	Specs	AO-080/42575528	CSc1ccc(cc1)S(=O)(=O)Nc1ccccc1C(=O)Nc1ccccc1Cl	n/a	≥90
**38**	MolPort-002-814-080	Specs	AH-487/42145232	COC(=O)c1ccc(NC(=O)c2ccccc2N(C)S(=O)(=O)c2ccccc2)cc1	n/a	≥90
**39**	MolPort-003-143-423	Life Chemicals Inc.	F2618-1733	COc1ccc(cc1)S(=O)(=O)Nc1ccccc1C(=O)Nc1ccc2OCCOc2c1	SOLID; s	≥90

**40**	MolPort-003-143-415	Life Chemicals Inc.	F2618-1224	COc1ccc(NC(=O)c2ccccc2NS(=O)(=O)c2ccc(Cl)cc2)c(c1)[N+]([O-])=O	SOLID; s	≥90
**41**	MolPort-003-143-414	Life Chemicals Inc.	F2618-1223	COc1cc(ccc1NC(=O)c1ccccc1NS(=O)(=O)c1ccc(Cl)cc1)[N+]([O-])=O	SOLID; s	≥90
**42**	MolPort-003-143-413	Life Chemicals Inc.	F2618-1222	COc1ccc(cc1NC(=O)c1ccccc1NS(=O)(=O)c1ccc(Cl)cc1)[N+]([O-])=O	SOLID; s	≥90
**43**	MolPort-003-143-406	Life Chemicals Inc.	F2618-0728	COc1ccc(NC(=O)c2ccccc2NS(=O)(=O)c2ccc(F)cc2)c(c1)[N+]([O-])=O	SOLID; s	≥90
**44**	MolPort-002-258-782	TimTec, LLC	ST094740	CSc1ccc(cc1)S(=O)(=O)Nc1ccccc1C(=O)Nc1ccc(F)cc1	n/a	n/a
**45**	MolPort-004-033-584	ChemDiv, Inc.	V029-0721	Cc1ccc(NC(=O)c2ccccc2NS(=O)(=O)c2ccc(F)c(F)c2)cc1	n/a	≥90
**46**	MolPort-005-822-708	ChemDiv, Inc.	V030-5404	Cc1ccc(NC(=O)c2ccccc2NS(=O)(=O)c2cccc(c2)C(F)(F)F)cc1	n/a	≥90
**47**	MolPort-006-650-380	ChemDiv, Inc.	V013-3582	Cc1cc(C)cc(NC(=O)c2ccccc2NS(=O)(=O)c2ccc(OC(F)(F)F)cc2)c1	n/a	≥90
**48**	MolPort-003-209-513	ChemDiv, Inc.	V030-5418	Cc1cccc(NC(=O)c2ccccc2NS(=O)(=O)c2cccc(F)c2)c1	n/a	≥90
**49**	MolPort-002-544-483	Vitas M Chemical Limited	STK093911	Clc1ccc(NC(=O)c2cc(Br)ccc2NS(=O)(=O)c2ccc(Cl)cc2)cc1	n/a	≥90
**50**	MolPort-001-602-512	Vitas M Chemical Limited	STK176303	COc1ccc(NC(=O)c2ccccc2NS(=O)(=O)c2cccc(c2)[N+]([O-])=O)cc1	n/a	≥90
**51**	MolPort-001-926-471	Vitas M Chemical Limited	STK832824	Clc1ccc(NC(=O)c2cc(Cl)ccc2NS(=O)(=O)c2ccc(Cl)cc2)cc1	n/a	≥90
**52**	MolPort-002-269-678	Vitas M Chemical Limited	STL255539	COC(=O)c1cc(NC(=O)c2ccccc2N(C)S(=O)(=O)c2ccccc2)ccc1Cl	n/a	≥90
**53**	MolPort-006-769-582	Vitas M Chemical Limited	STL282133	CN(C(=O)c1ccccc1NS(=O)(=O)c1ccc(C)cc1)c1ccccc1	n/a	≥90
**54**	MolPort-001-602-314	Vitas M Chemical Limited	STK204271	COc1ccc(NC(=O)c2ccccc2NS(=O)(=O)c2cc(Cl)ccc2Cl)cc1	n/a	≥90
**55**	MolPort-002-293-192	Vitas M Chemical Limited	STL087418	CN(c1cccc(NC(=O)c2ccccc2NS(=O)(=O)c2ccccc2)c1)S(C)(=O)=O	n/a	≥90
**56**	MolPort-002-299-522	Vitas M Chemical Limited	STL071981	CC(C)c1ccccc1NC(=O)c1ccccc1NS(=O)(=O)c1ccc(C)cc1	n/a	≥90
**57**	MolPort-002-279-710	Vitas M Chemical Limited	STL118782	FC(F)(F)c1cccc(NC(=O)c2ccccc2NS(=O)(=O)c2ccccc2)c1	n/a	≥90
**58**	MolPort-002-280-084	Vitas M Chemical Limited	STL119063	COC(=O)c1ccccc1NC(=O)c1ccccc1NS(=O)(=O)c1ccccc1	n/a	≥90
**59**	MolPort-006-797-978	Vitas M Chemical Limited	STL121500	CSc1cccc(NC(=O)c2ccccc2N(C)S(=O)(=O)c2ccccc2)c1	n/a	≥90
**60**	MolPort-002-290-007	Vitas M Chemical Limited	STL114459	CSc1cccc(NC(=O)c2ccccc2NS(=O)(=O)c2ccc(C)cc2)c1	n/a	≥90
**61**	MolPort-002-282-825	Vitas M Chemical Limited	STL101276	COC(=O)c1cc(NC(=O)c2ccccc2NS(=O)(=O)c2ccccc2)ccc1Cl	n/a	≥90
**62**	MolPort-002-279-964	Vitas M Chemical Limited	STL118945	CCOc1ccccc1NC(=O)c1ccccc1NS(=O)(=O)c1ccccc1	n/a	≥90
**63**	MolPort-002-271-214	Vitas M Chemical Limited	STL121546	CN(c1ccccc1C(=O)Nc1ccc(Cl)c(c1)C(F)(F)F)S(=O)(=O)c1ccc(C)cc1	n/a	≥90
**64**	MolPort-002-729-822	Vitas M Chemical Limited	STK229377	CC(=O)NCCc1ccc(cc1)S(=O)(=O)Nc1ccccc1C(=O)Nc1ccccc1	n/a	≥90
**65**	MolPort-002-729-855	Vitas M Chemical Limited	STK229389	FC(F)(F)c1cccc(c1)S(=O)(=O)Nc1ccccc1C(=O)Nc1cccc(Cl)c1	n/a	≥90
**66**	MolPort-001-908-026	Vitas M Chemical Limited	STK826064	COC(=O)c1ccc(NC(=O)c2ccccc2NS(=O)(=O)c2ccc(C)cc2)cc1	n/a	≥90
**67**	MolPort-002-296-763	Vitas M Chemical Limited	STL072047	CSc1cccc(NC(=O)c2ccccc2NS(=O)(=O)c2ccccc2)c1	n/a	≥90
**68**	MolPort-002-297-282	Vitas M Chemical Limited	STL071766	Cc1ccc(cc1)S(=O)(=O)Nc1ccccc1C(=O)Nc1ccc(cc1)C(F)(F)F	n/a	≥90

**69**	MolPort-000-752-317	Vitas M Chemical Limited	STK525252	Clc1ccc(cc1)C(=O)c1ccc(NC(=O)c2cc(Br)ccc2NS(=O)(=O)c2ccc(Cl)cc2)cc1Cl	n/a	≥90
**70**	MolPort-002-747-441	Vitas M Chemical Limited	STK665290	CC(C)c1ccccc1NC(=O)c1ccccc1NS(=O)(=O)c1ccc(Cl)cc1	n/a	≥90
**71**	MolPort-001-950-614	Vitas M Chemical Limited	STK758752	Clc1ccc(NC(=O)c2cc3ccccc3cc2NS(=O)(=O)c2ccccc2)cc1	n/a	≥90
**72**	MolPort-002-134-495	ChemBridge Corporation	5175496	Clc1ccc(cc1)C(=O)c1cc(Cl)ccc1NC(=O)c1cc(Br)ccc1NS(=O)(=O)c1ccc(Cl)cc1	SOLID; Solid	≥90
**73**	MolPort-002-228-464	ChemBridge Corporation	6942753	NC(=O)c1ccccc1NC(=O)c1ccccc1NS(=O)(=O)c1ccc(Cl)cc1	SOLID; Solid	≥90
**74**	MolPort-002-276-738	ChemBridge Corporation	7900286	CCc1ccc(NC(=O)c2ccccc2NS(=O)(=O)c2cccc(c2)[N+]([O-])=O)cc1	SOLID; Solid	≥90

NMR Data

N-(3,4-Dichlorophenyl)-2-[(3-nitrophenyl)sulfonamido]benzamide (1)

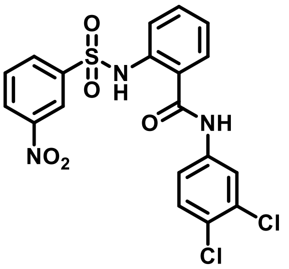

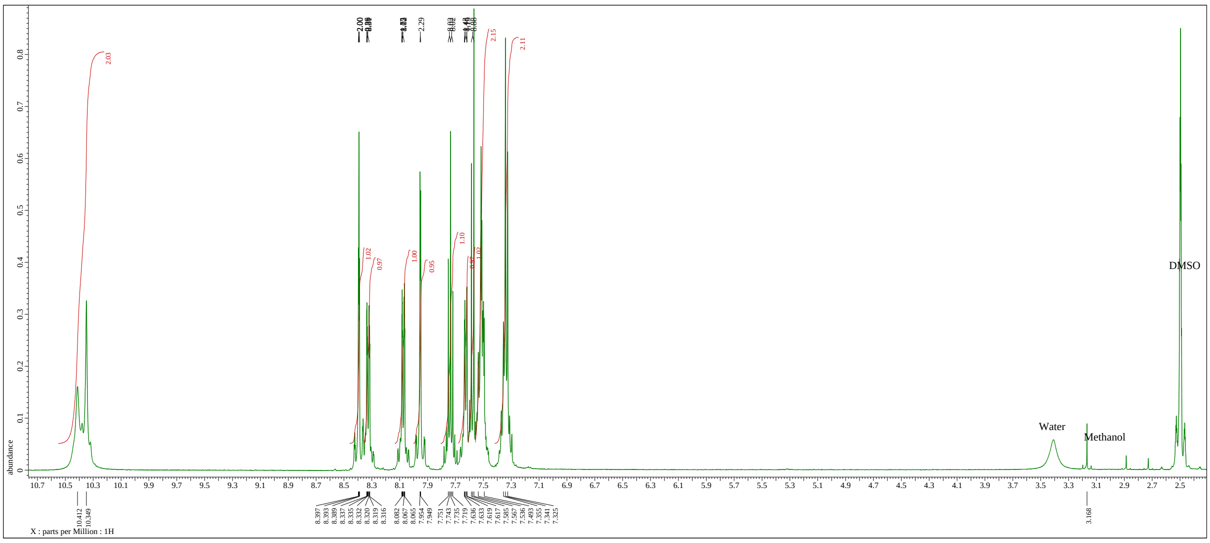

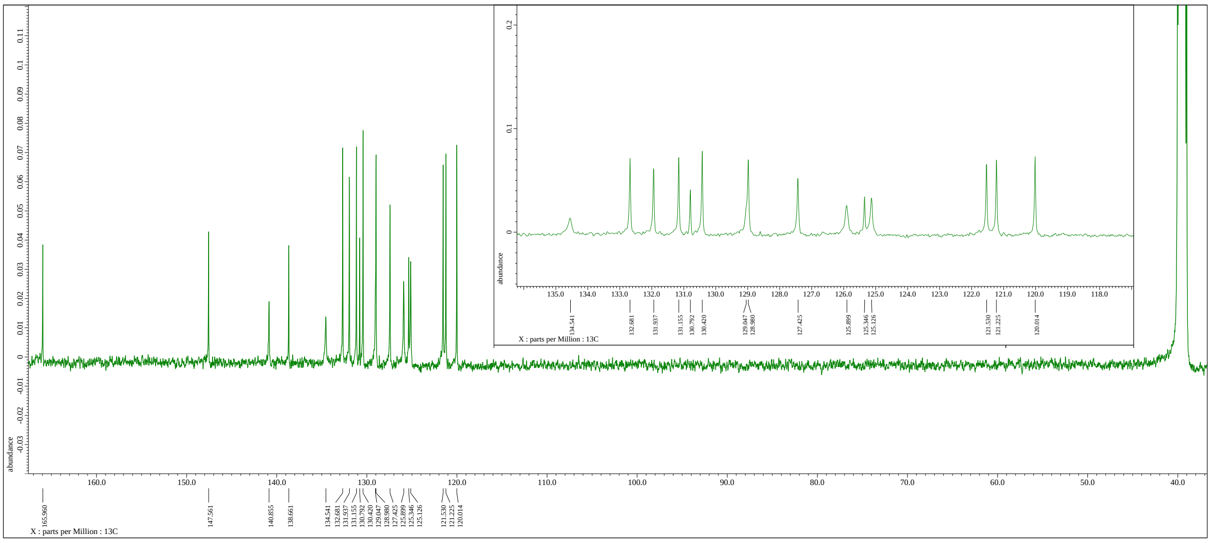

N-(3,4-dichlorophenyl)-2-[(2-nitrophenyl)sulfonamido]benzamide (3)

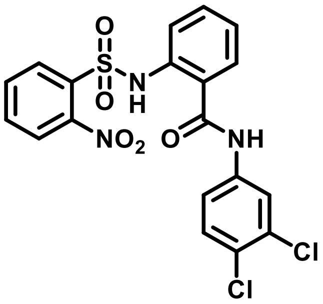

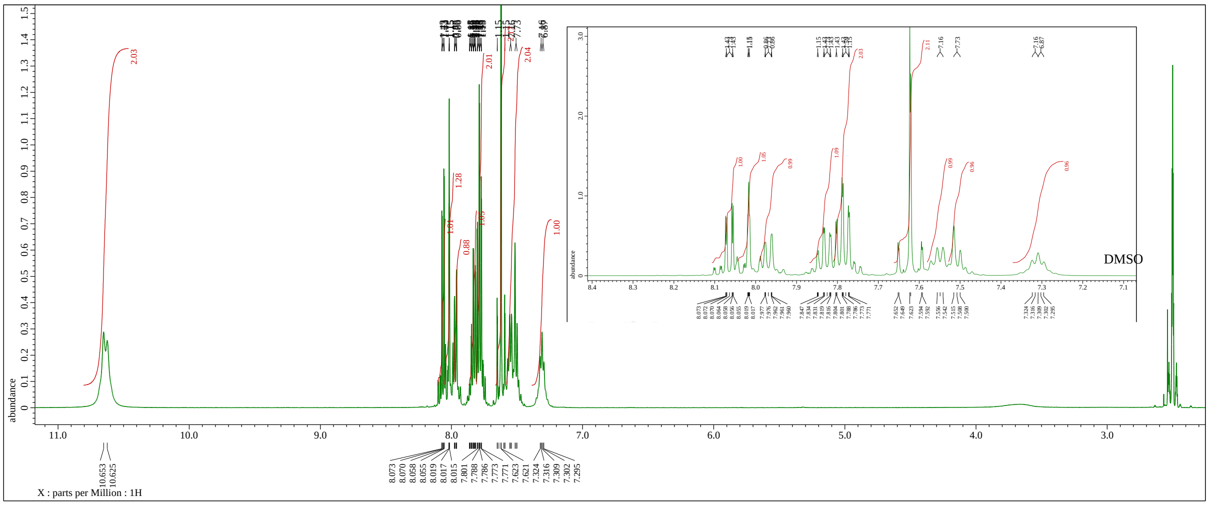

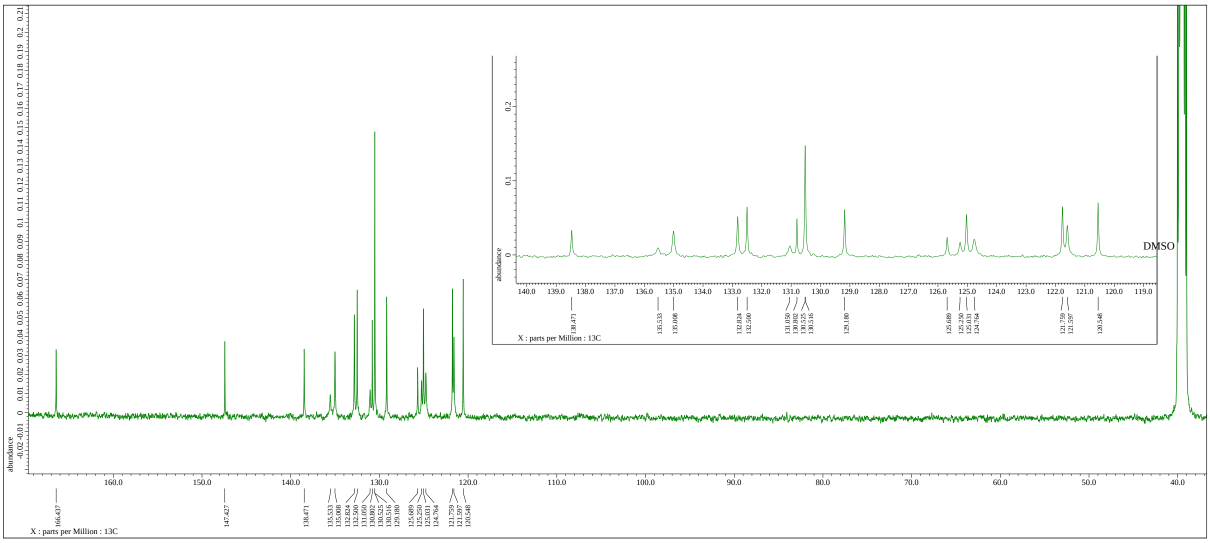

2-[(3-Cyanophenyl)sulfonamido)-*N*-(3,4-dichlorophenyl)benzamide (4)

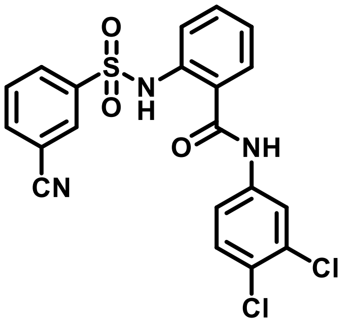

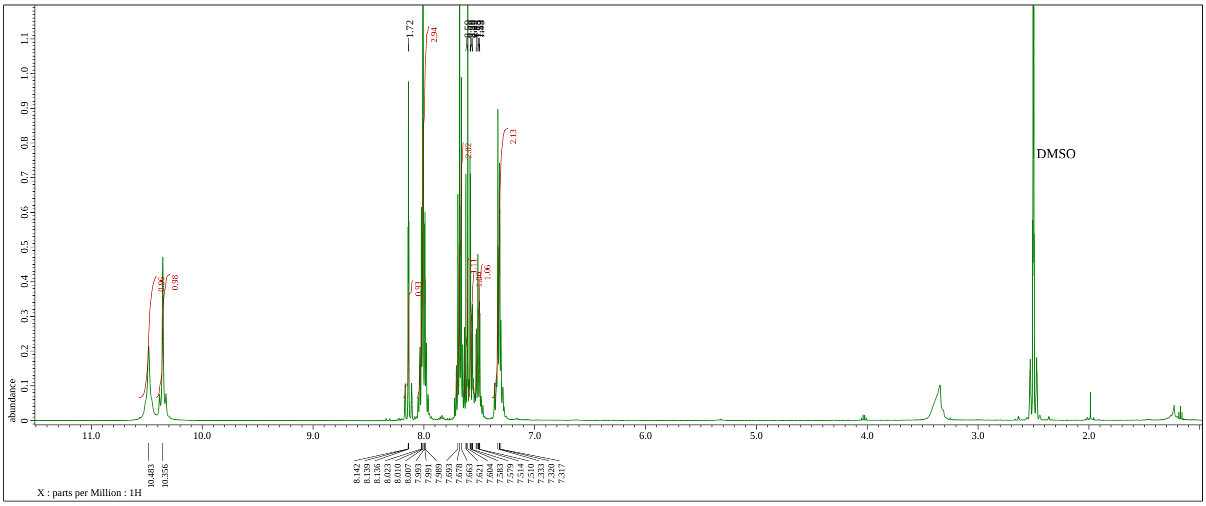

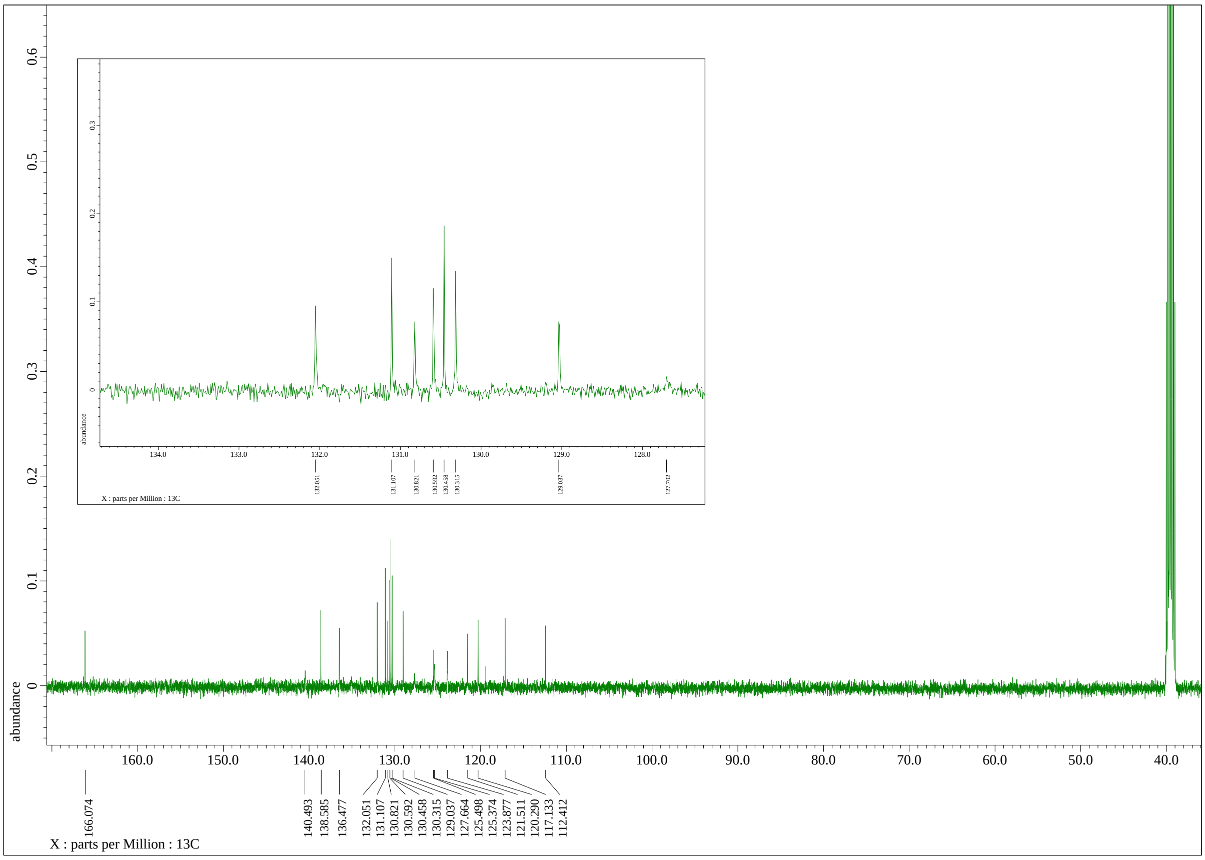

2-{[3-(1*H*-Tetrazol-5-yl)phenyl]sulfonamido}-*N*-(3,4-dichlorophenyl)benzamide (5)

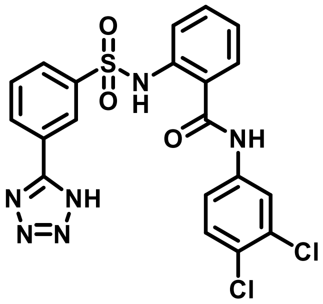

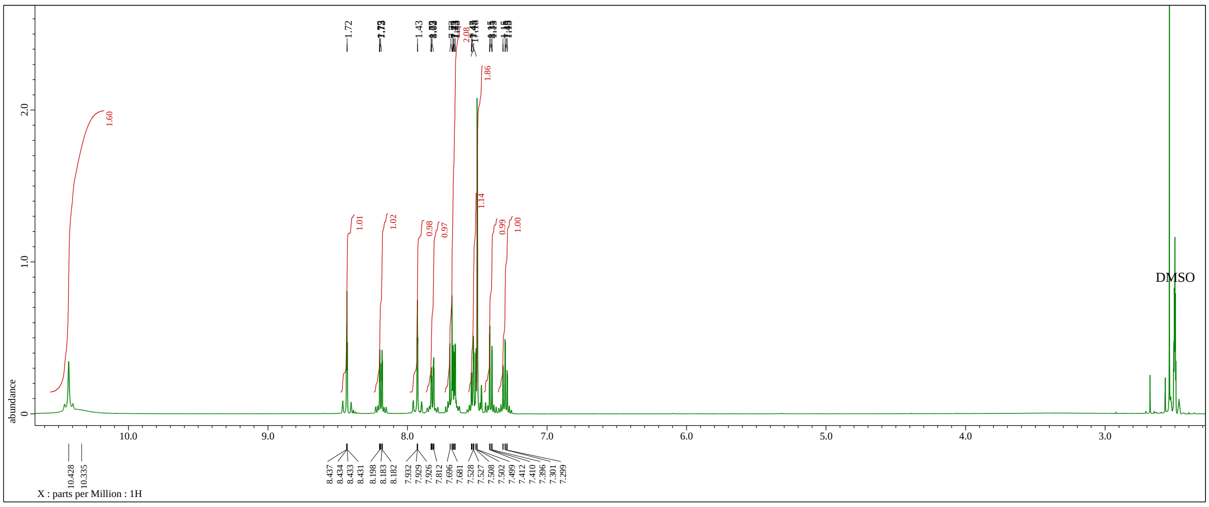

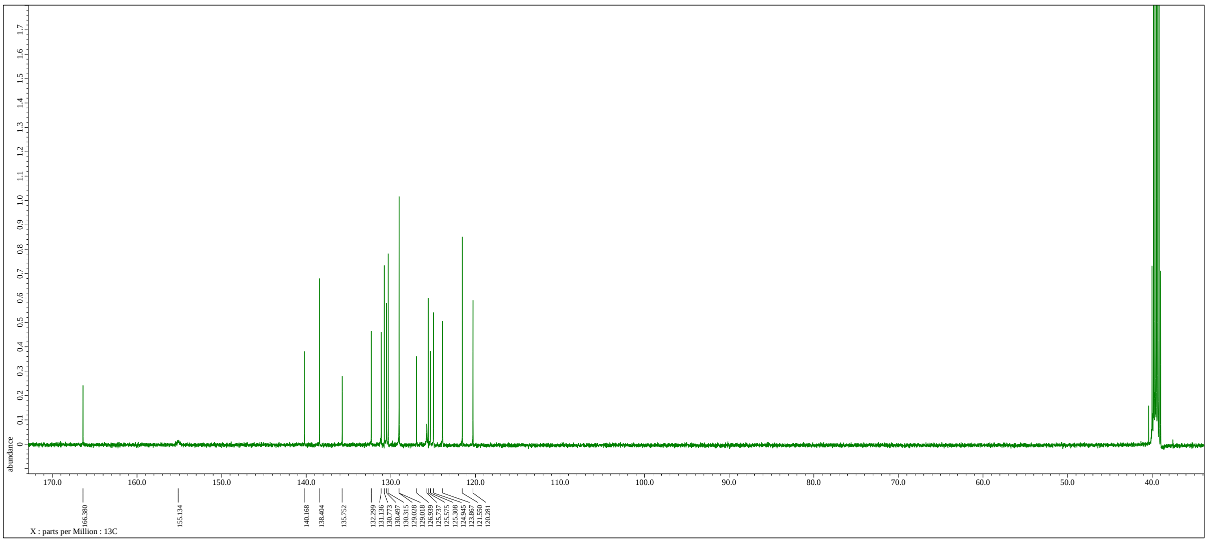

3-(*N*-{2-[(3,4-dichlorophenyl)carbamoyl]phenyl}sulfamoyl)benzoic acid (6)

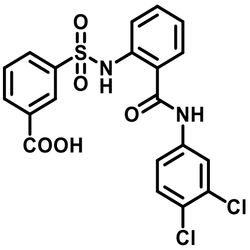

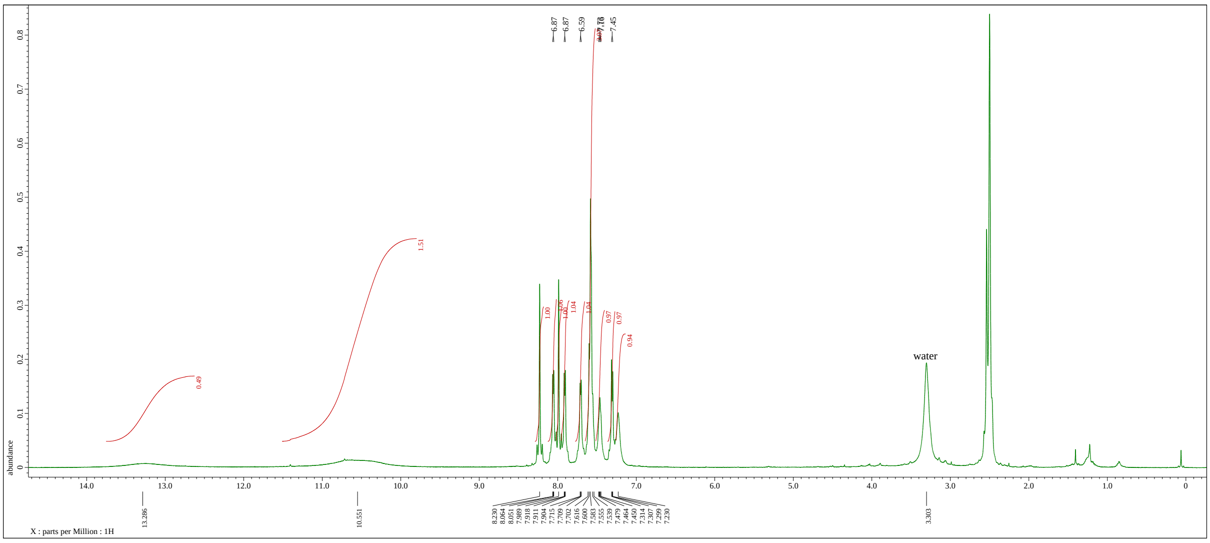

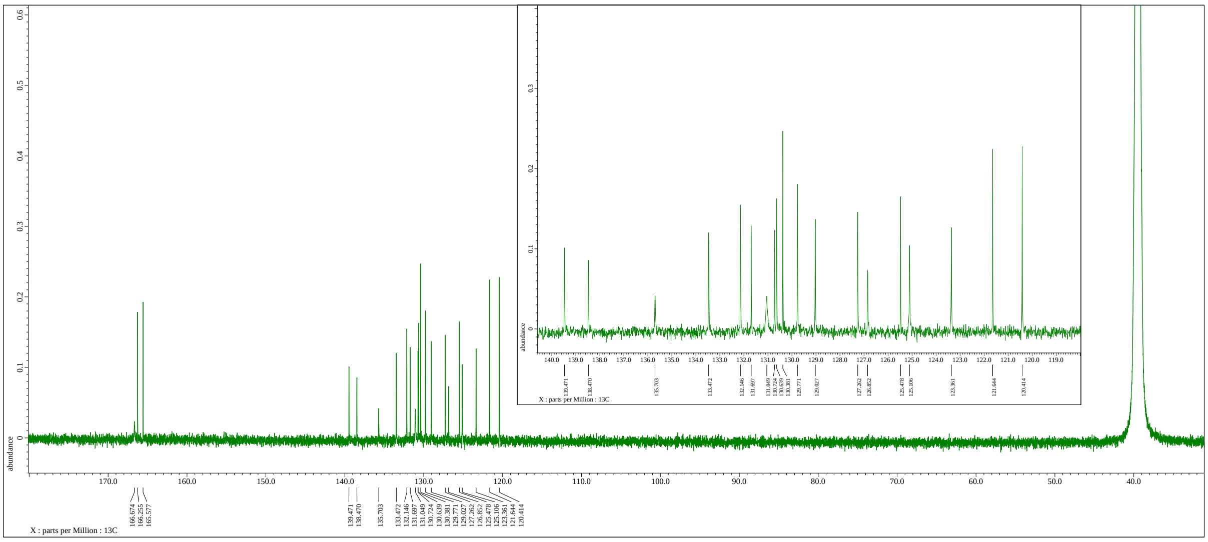

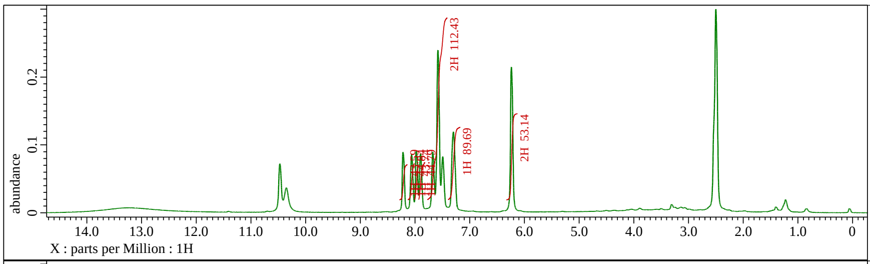


```
Compound                = maleic acid
Integral Position       = 6.228[ppm]
Integral Value(I)       = 18.47[abn]
Molecular Weight(M)     = 116.07
Protons(H)              = 2
Weight(m)               = 4.32[mg]
Volume(L)               = 0.7[mL]
Volume Conc.(V)         = 0.61714[%(w/v)]
Purity(P)               = 99.94[%]
Molarity(C)             = 53.13798[mmol/L]
recvr_gain(G)           = 40
temp_get(T)             = 303.16[K]
x_pulse(d)              = 5.725[us]
scale factor(S)         =
average factor(A)       =
normalize factor(N)     = 1
```

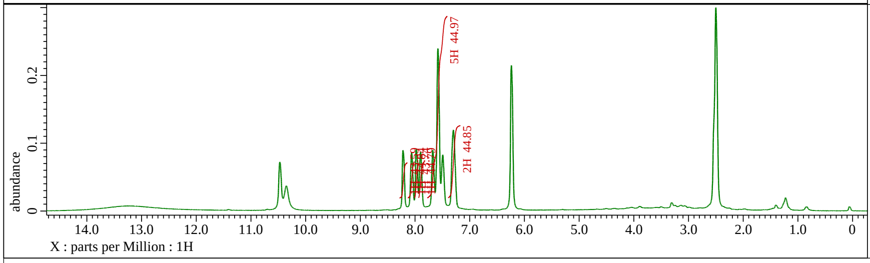


```
Compound                = (6)
Molecular Weight(M')    = 465.3
Mixture                 = FALSE
Weight(m')              = 15.08[mg]
Volume(L')              = 0.7[mL]
Volume Conc.(V')        = 2.15429[%(w/v)]
recvr_gain(G')          = 40
temp_get(T')            = 303.16[K]
x_pulse(d')             = 5.725[us]
scale factor(S')        =
average factor(A')      =
normalize factor(N')    = 1
```

Position	Deconv.	Group	Integral(I')	Protons(H')	Molarity(C')	Purity(P')	Avg. Calc.
7.283[ppm]			15.59[abn]	2	44.85[mmol/L]	96.86[%]	*
7.593[ppm]			39.09[abn]	5	44.97[mmol/L]	97.13[%]	*
7.880[ppm]			7.79[abn]	1	44.79[mmol/L]	96.74[%]	*
7.973[ppm]			7.60[abn]	1	43.74[mmol/L]	94.47[%]	*
8.071[ppm]			7.79[abn]	1	44.84[mmol/L]	96.85[%]	*
8.212[ppm]			7.58[abn]	1	43.59[mmol/L]	94.15[%]	*

Gain Scale = 20, Avg. Molarity = 44.46[mmol/L], Avg. Purity = 96.03[%]
Molarity : C' = C * I'/I * H/H' * T'/T * d'/d, Purity : P' = C' * M' / (100 * V')

2-(Benzo[*c*][1,2,5]oxadiazole-4-sulfonamido)-*N*-(3,4-dichlorophenyl)benzamide (7)

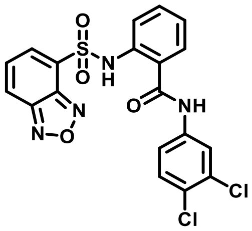

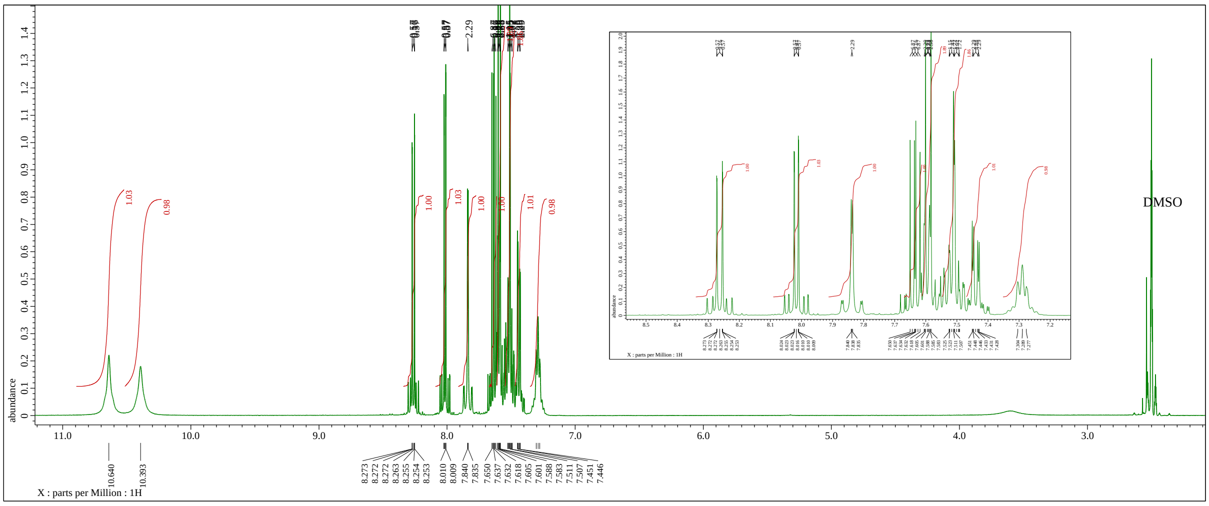

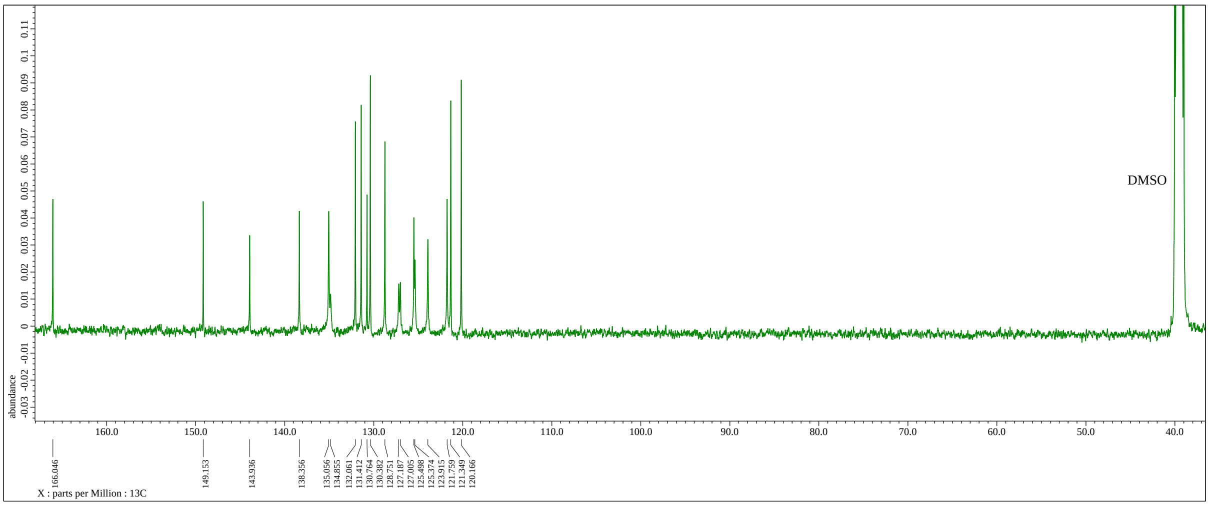

2-[(4-Chloro-3-nitrophenyl)sulfonamido]-*N*-(3,4-dichlorophenyl)benzamide (11)

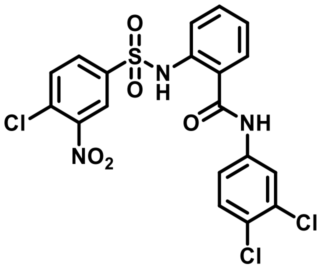

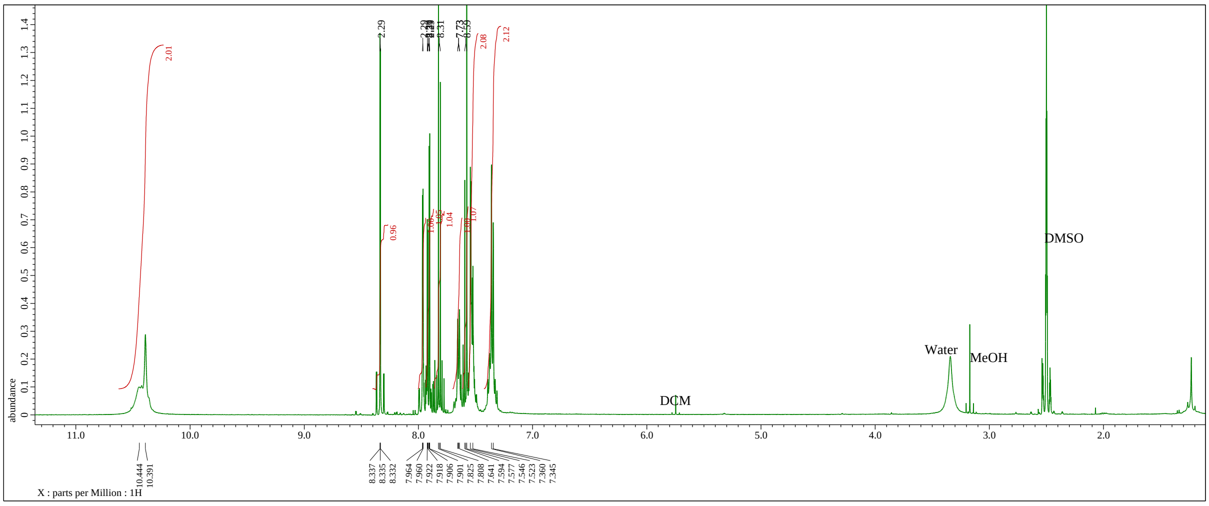

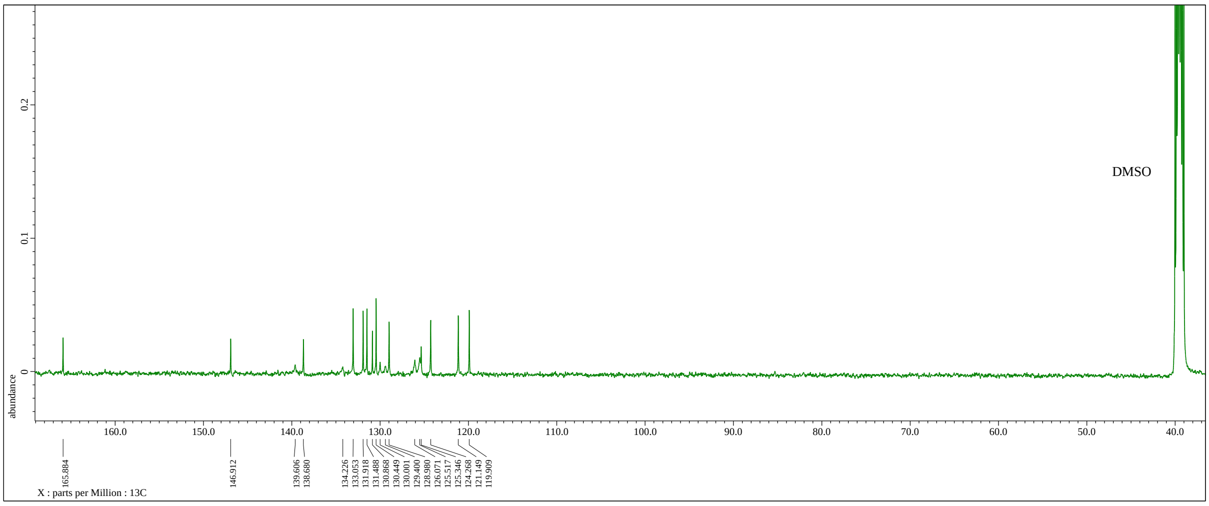

5-Chloro-*N*-(3,4-dichlorophenyl)-2-[(3-nitrophenyl)sulfonamido]benzamide (12)

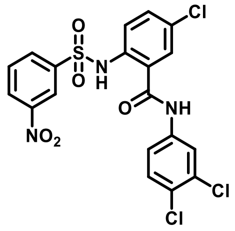

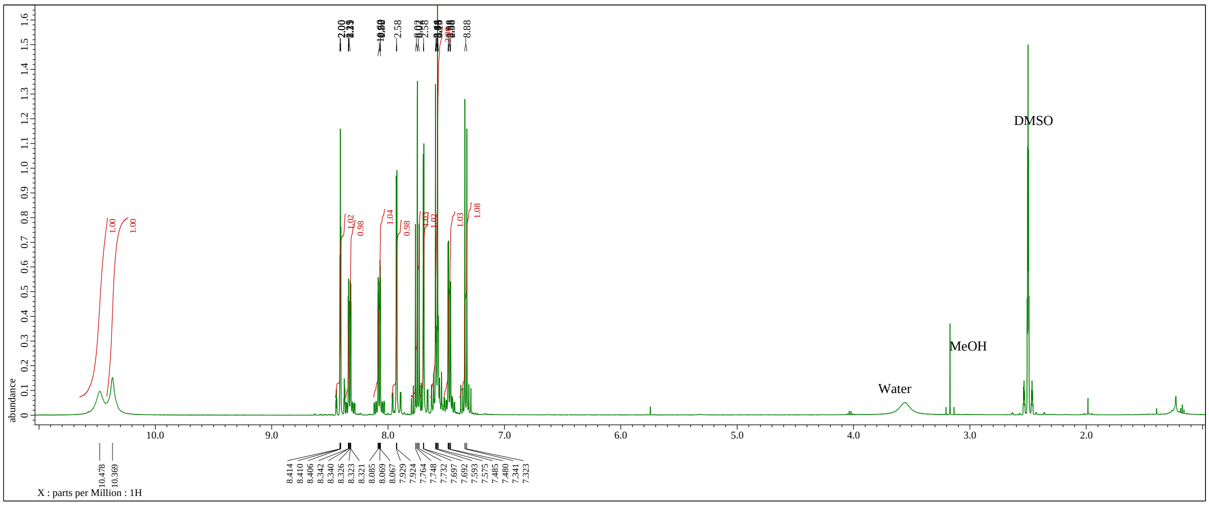

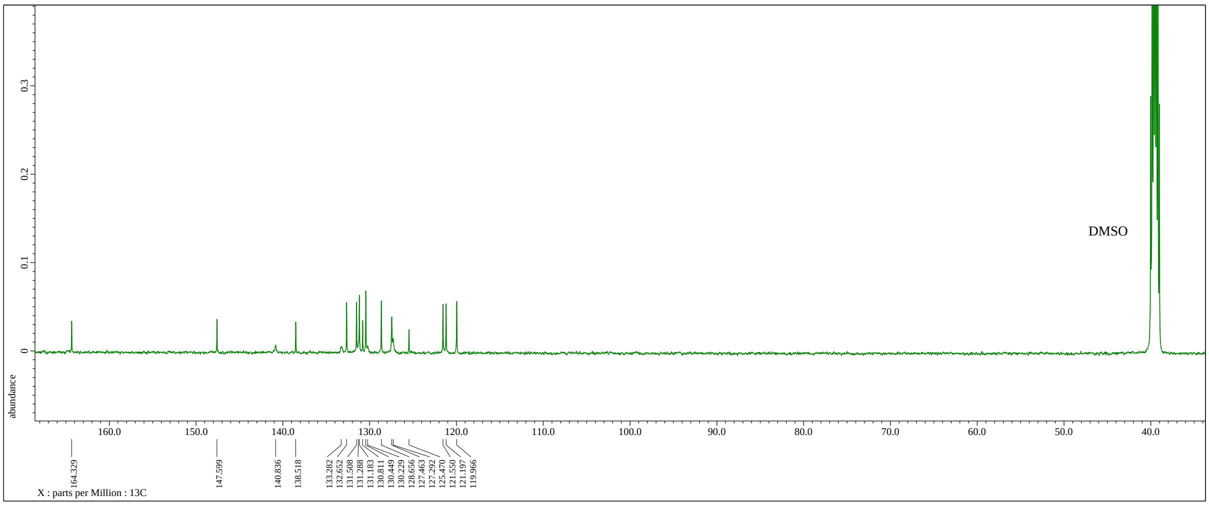

5-Chloro-2-[(4-chloro-3-nitrophenyl)sulfonamido]-*N*-(3,4-dichlorphenyl)benzamide (13)

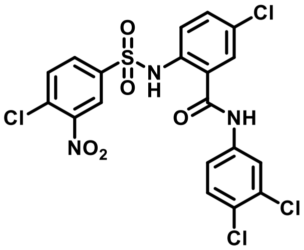

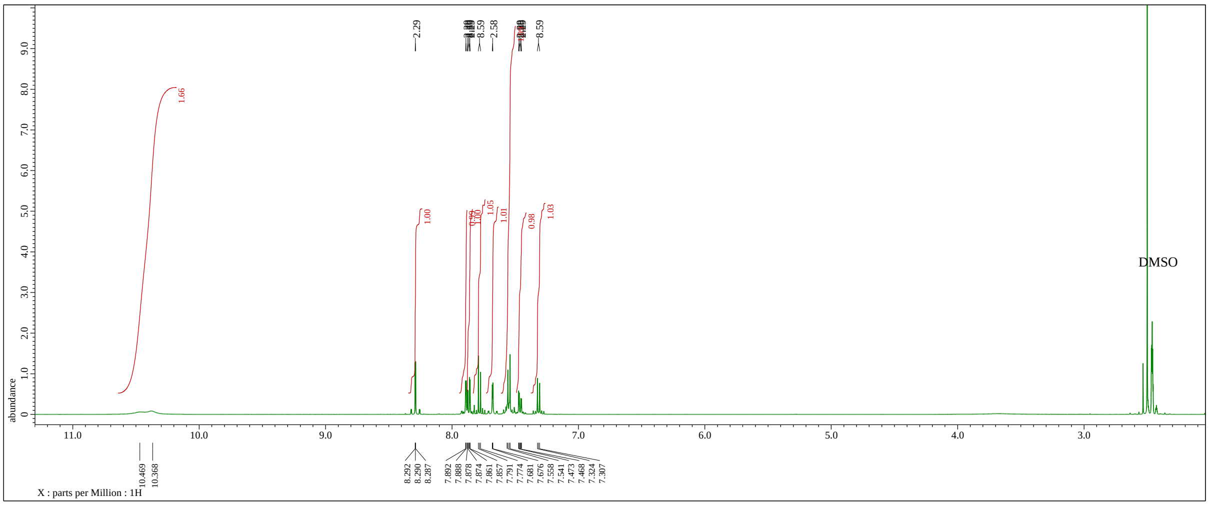

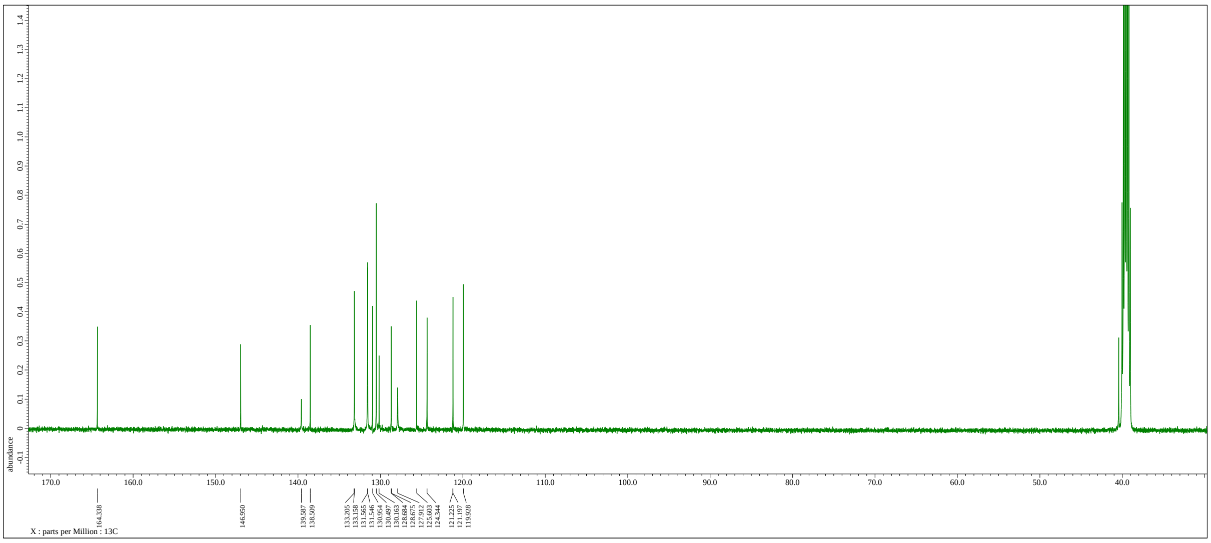

N-(2,4-Dichlorophenyl)-2-[(3-nitrophenyl)sulfonamido]benzamide (20)

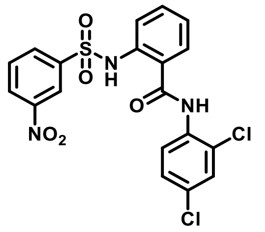

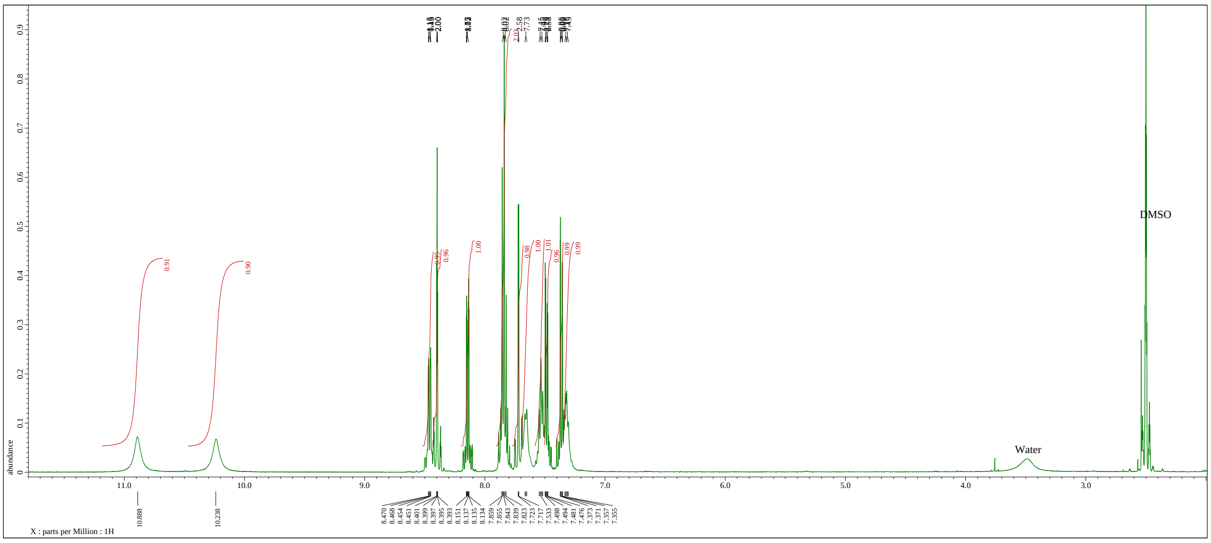

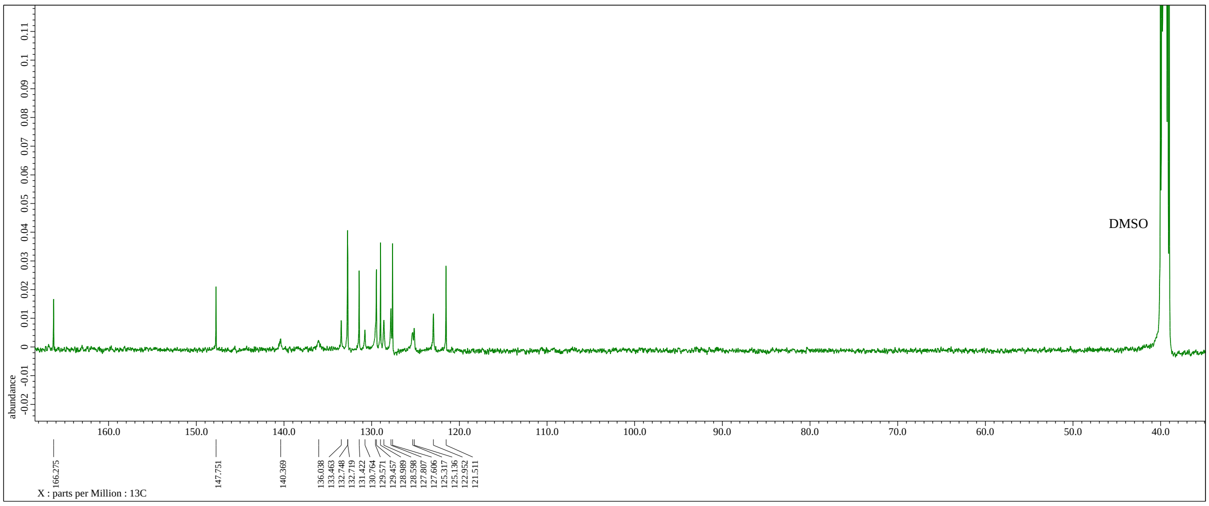

N-(3,4-Dichlorophenyl)-3-[(3-nitrophenyl)sulfonamido]propanamide (21)

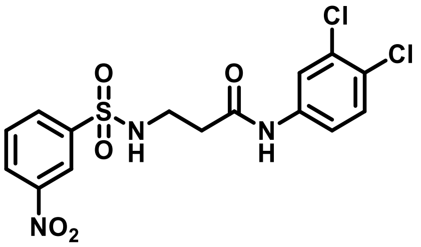

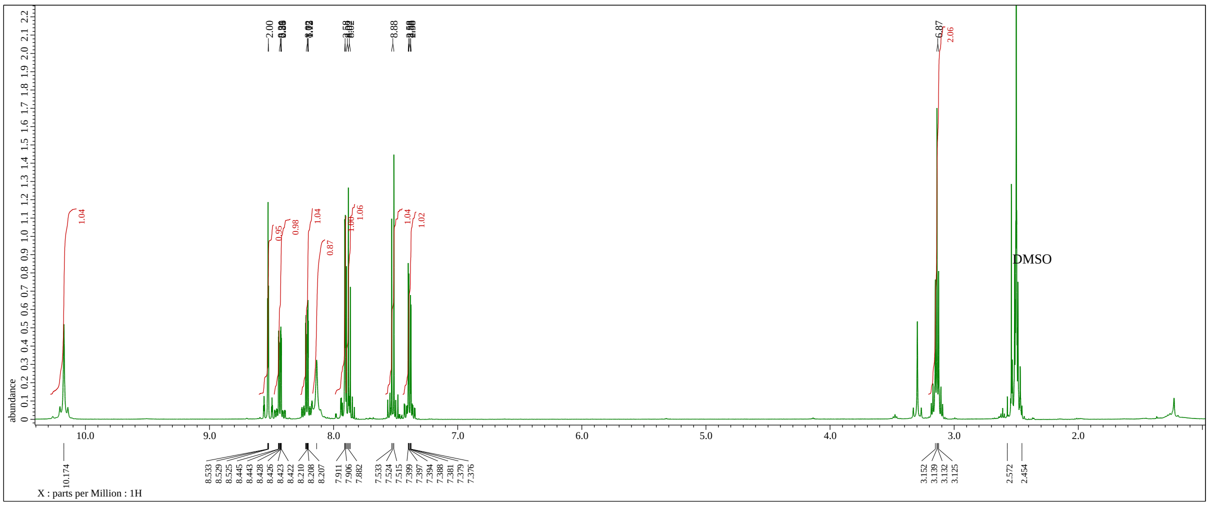

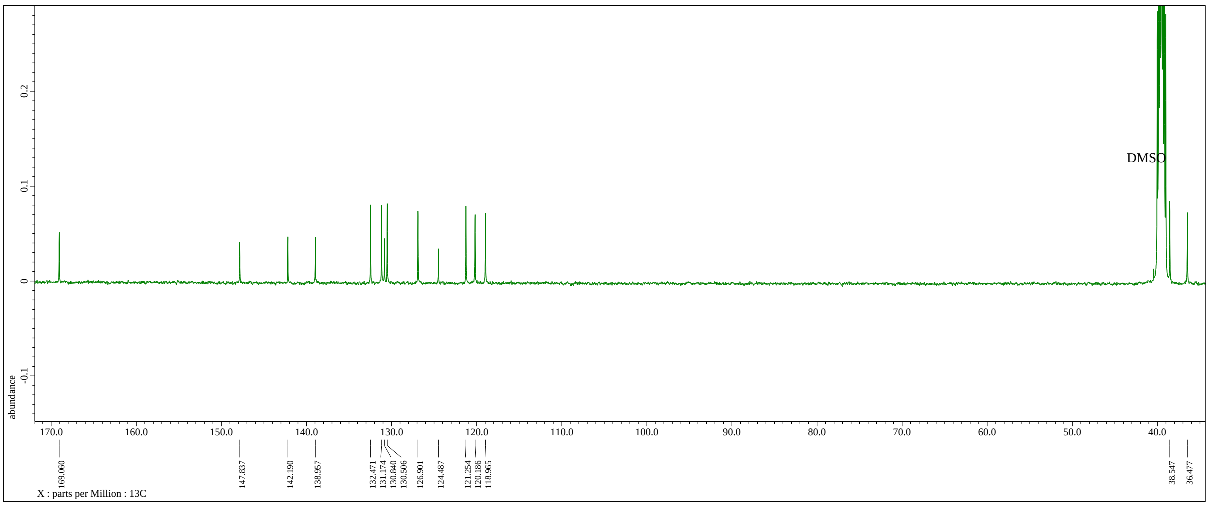

N-(3,4-Dichlorophenyl)-2-[(*N*-methyl-3-nitrophenyl)sulfonamido]benzamide (22)

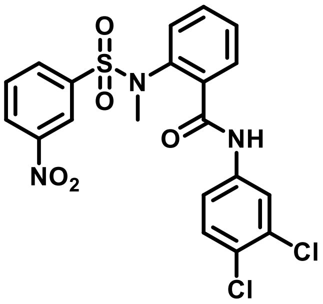

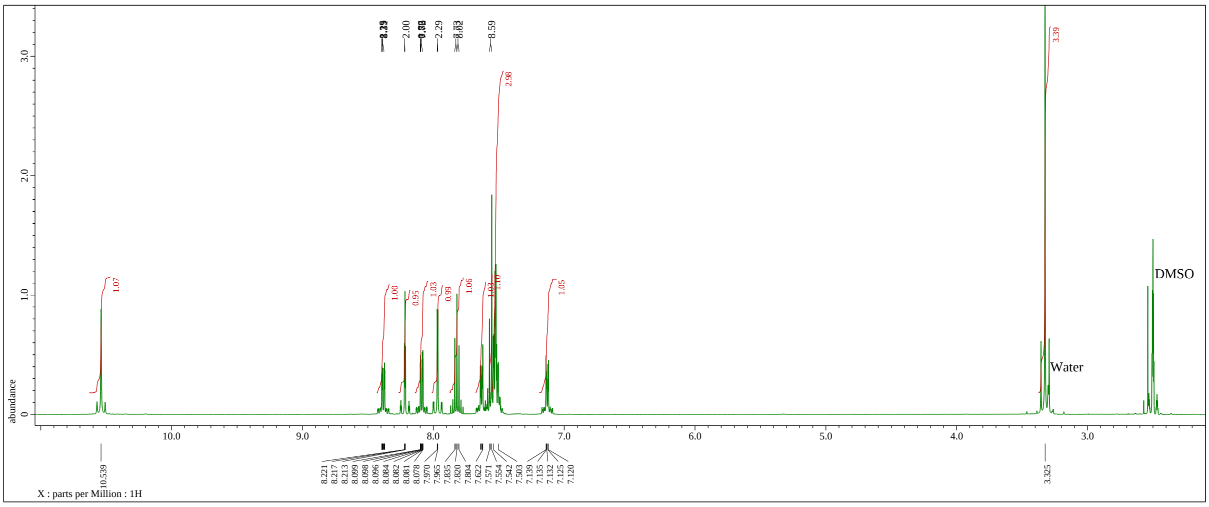

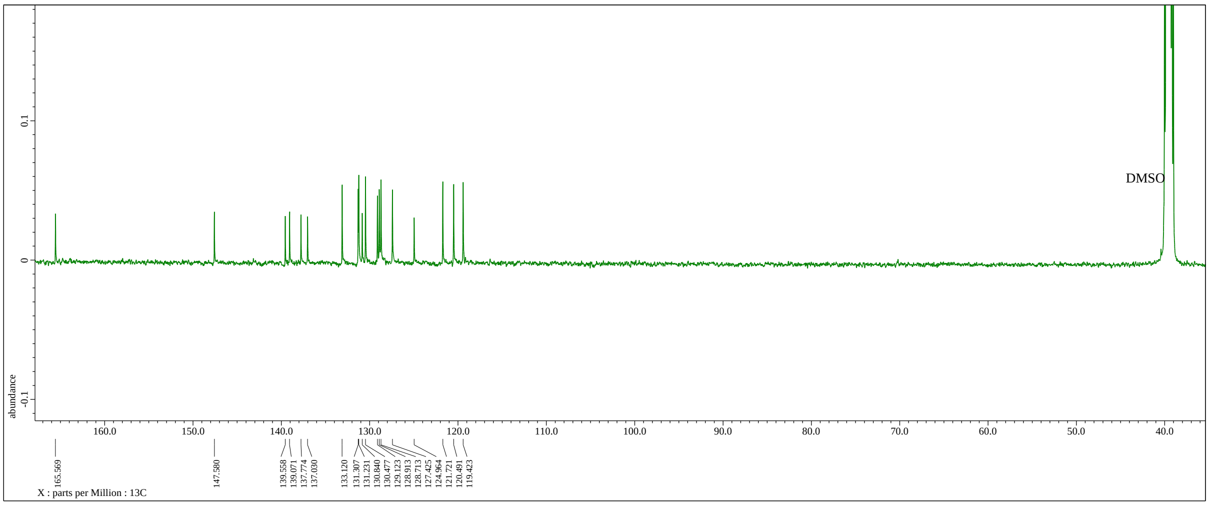

N-(3,4-Dichlorophenyl)-2-(3-nitrobenzamido)benzamide (23)

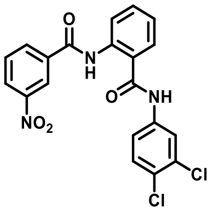

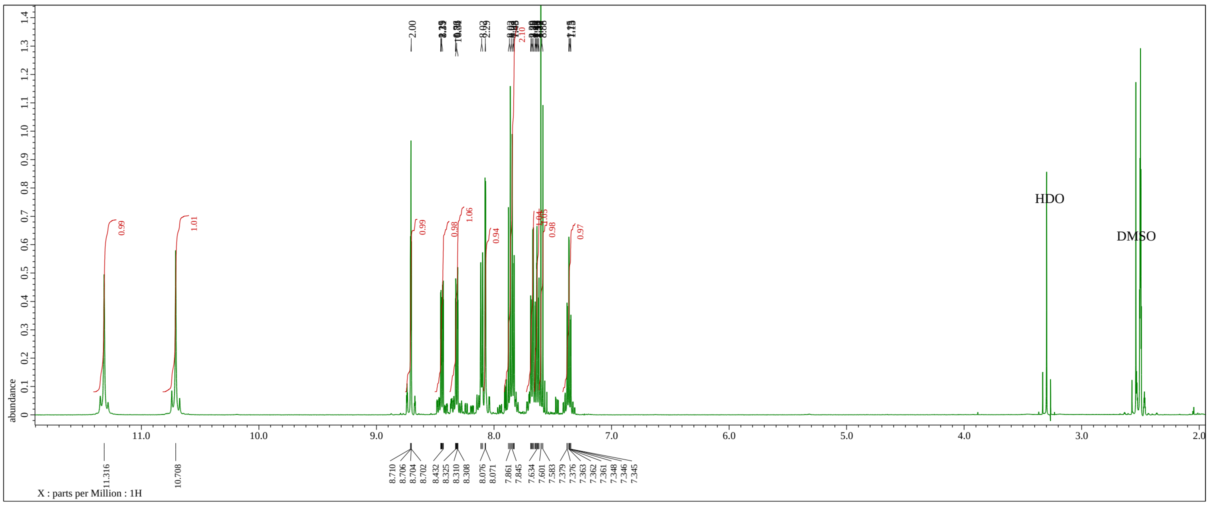

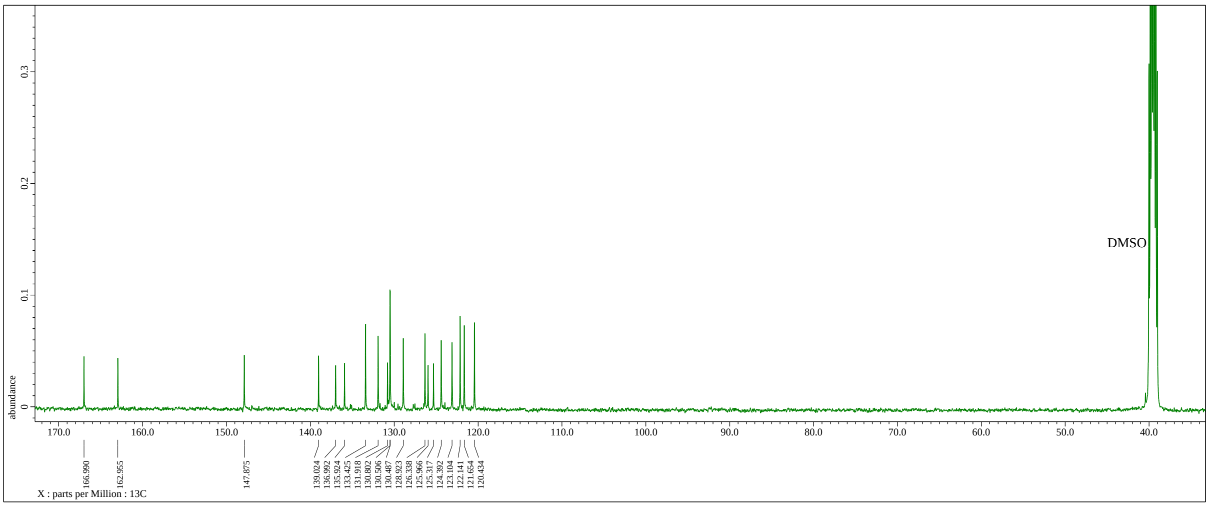

N-(3,4-Dichlorophenyl)-3-[(3-nitrophenyl)sulfonamido]thiophene-2-carboxamide (24)

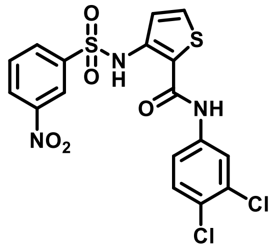

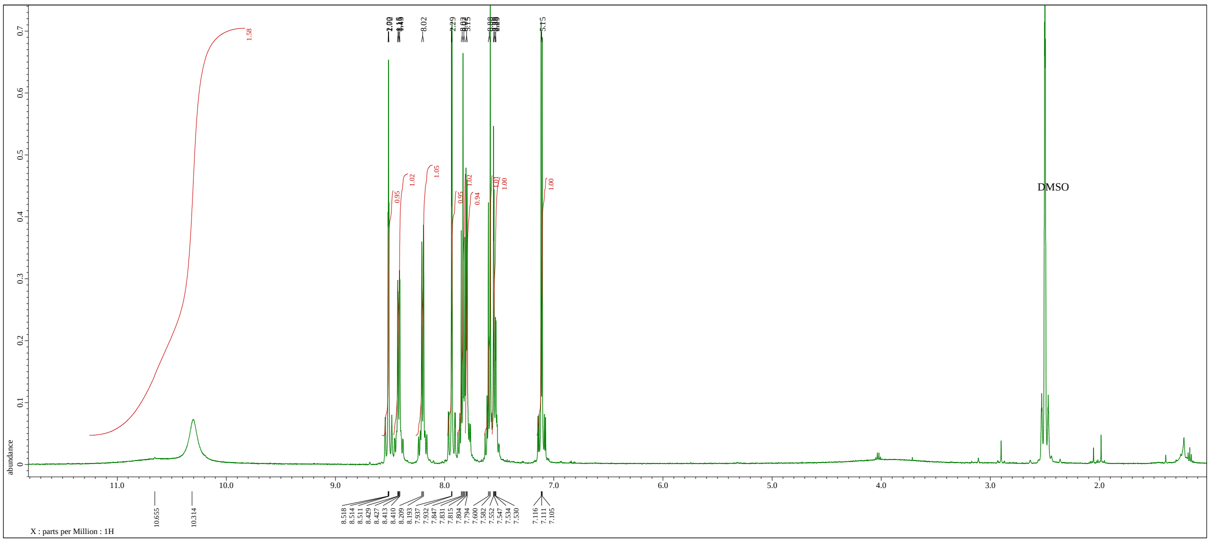

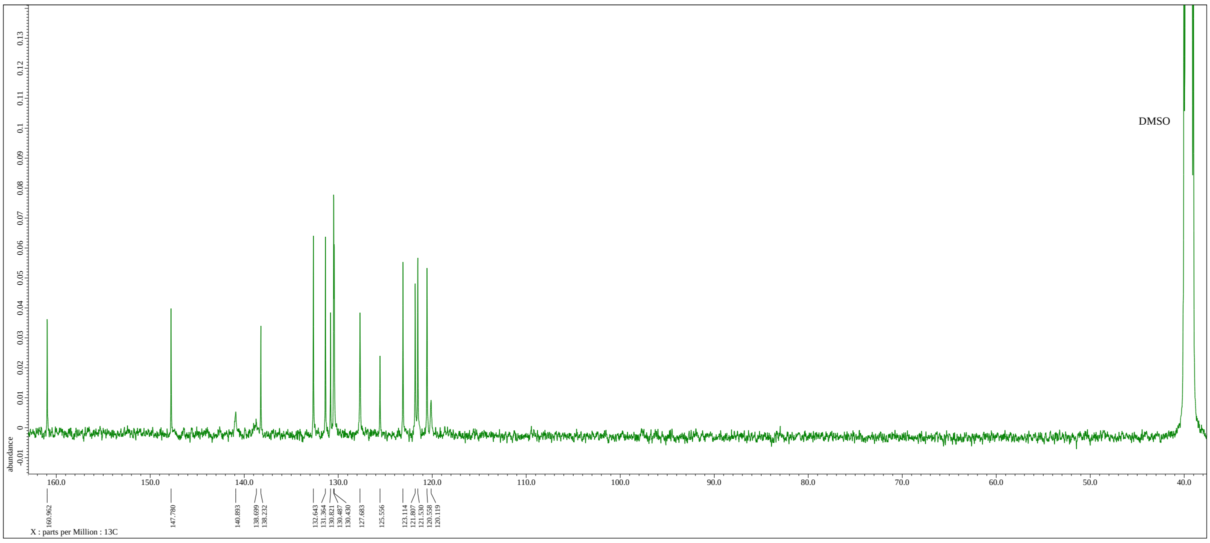

Methyl-2-[(3-nitrophenyl)sulfonamido]benzoate (28)

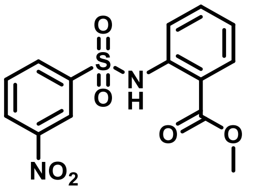

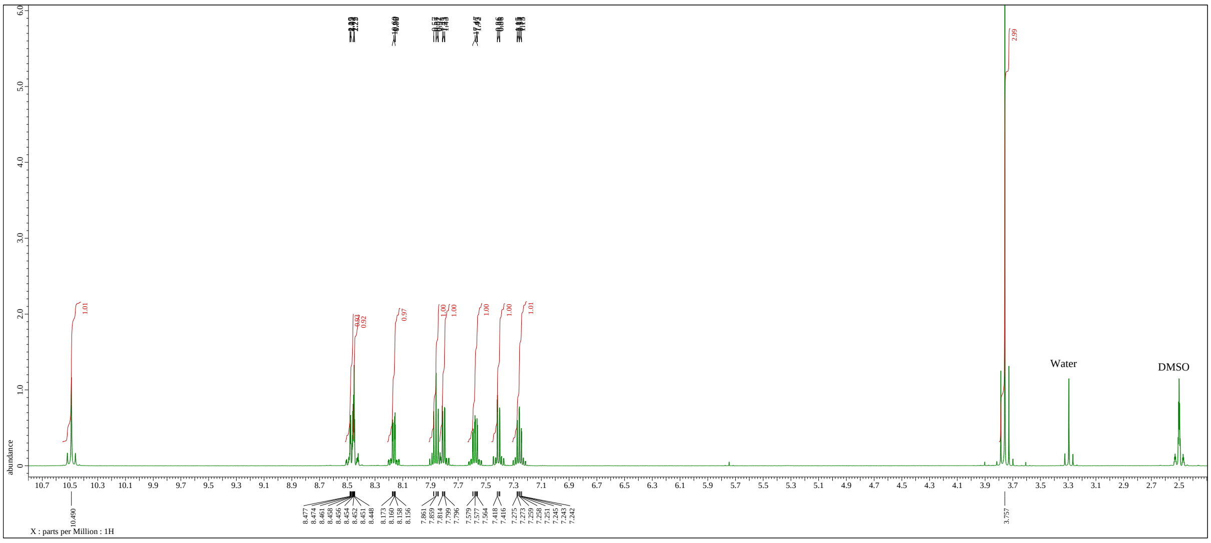

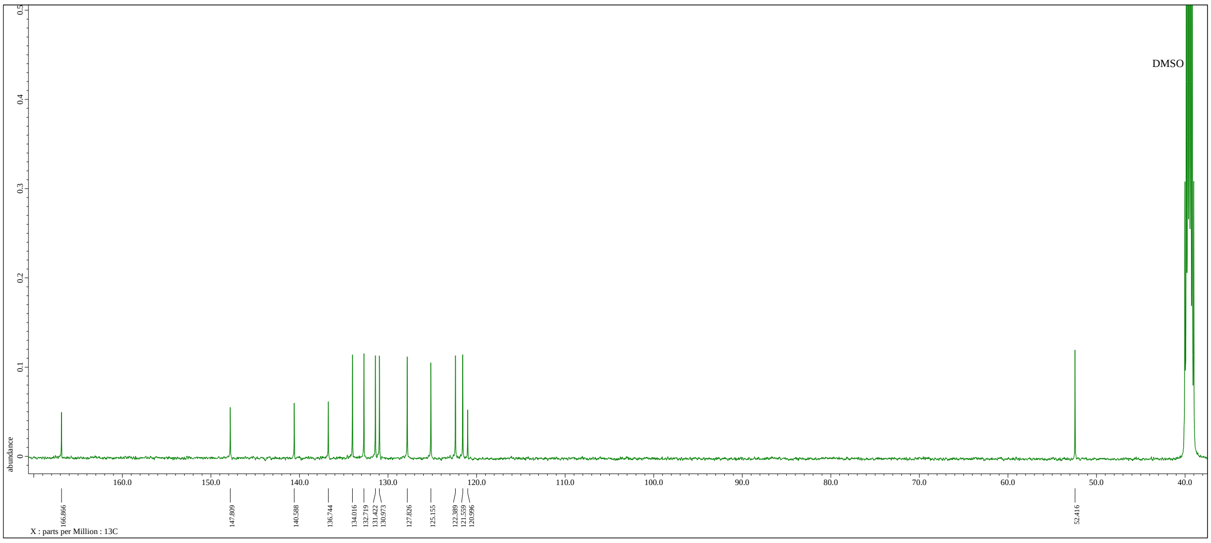

2-[(3-Nitrophenyl)sulfonamido]benzoic acid (76)

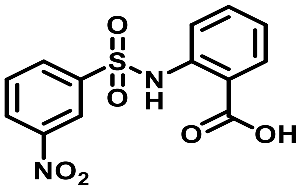

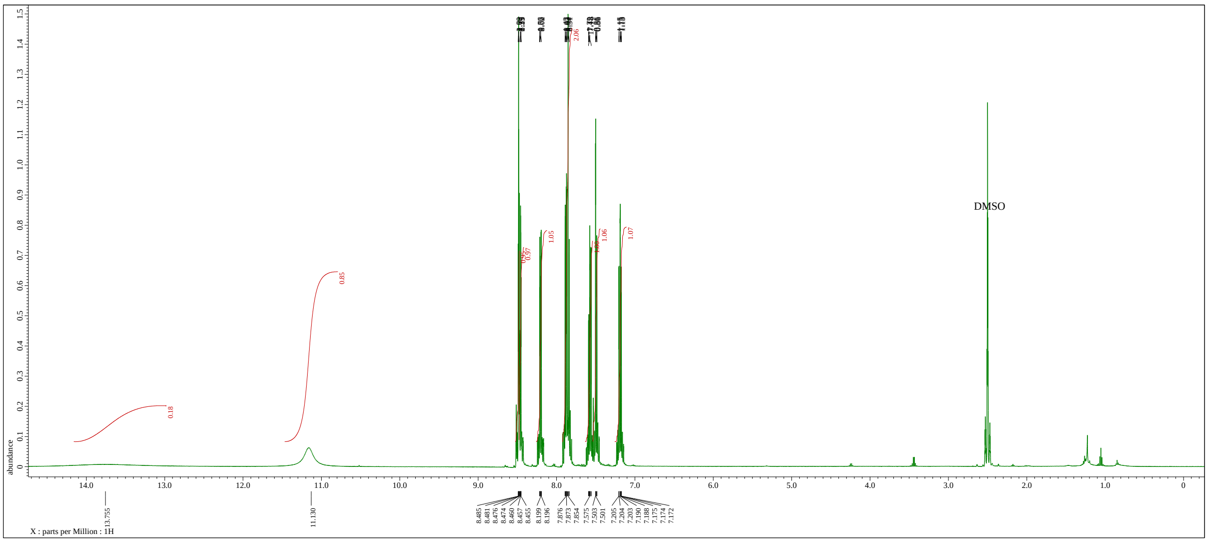

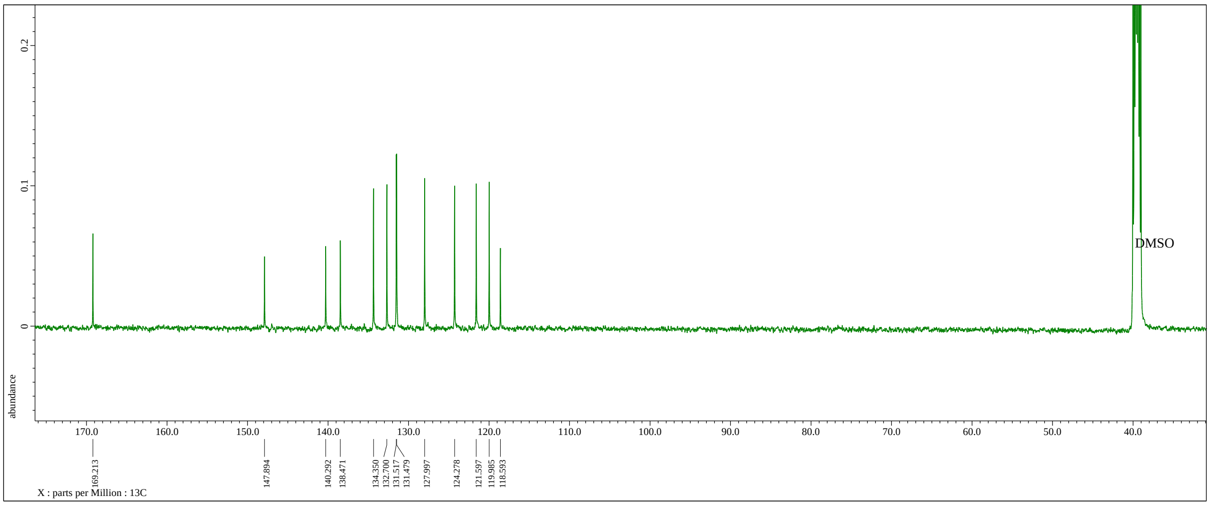

tert-Butyl-(4-chloro-2-[(3,4-dichlorophenyl)carbamoyl)phenyl]carbamate (83)

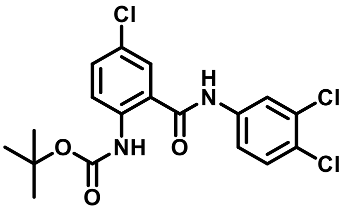

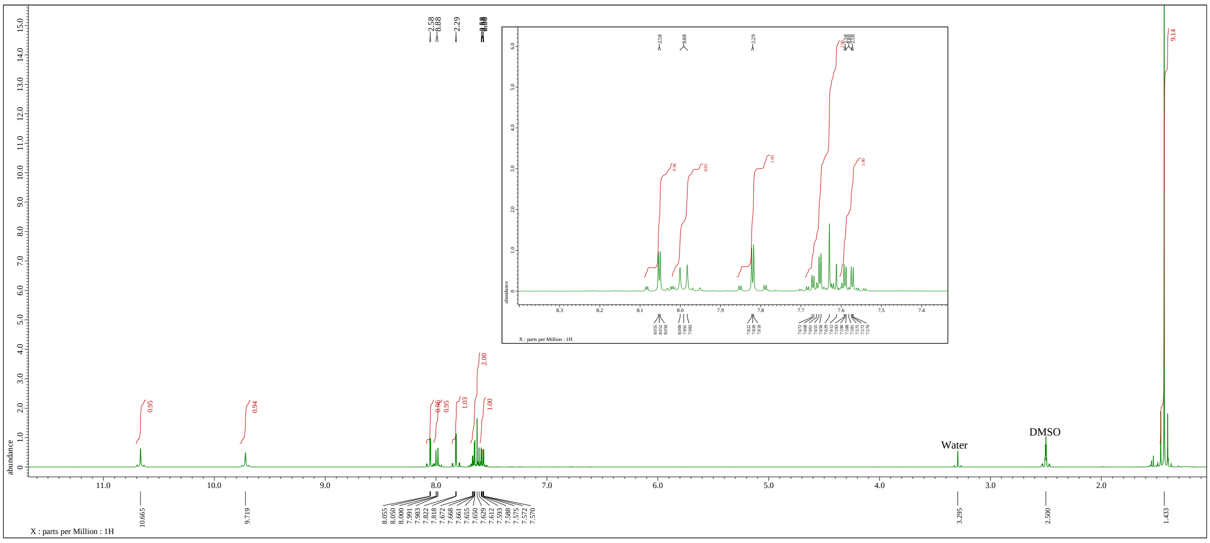

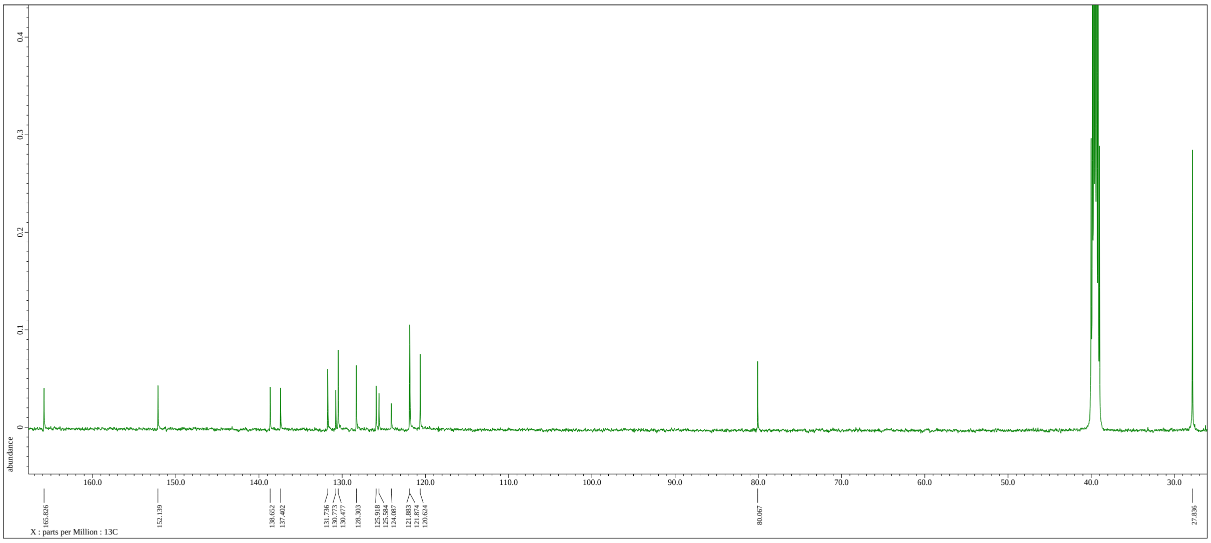

N-(3,4-Dichlorophenyl)-2-(methylamino)benzamide (89)

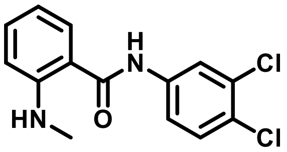

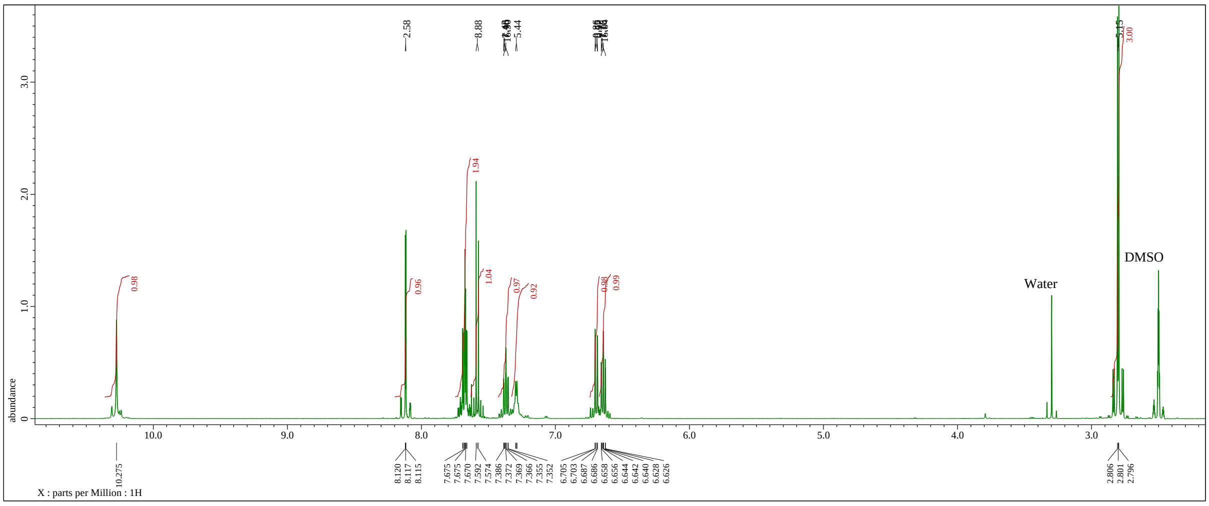

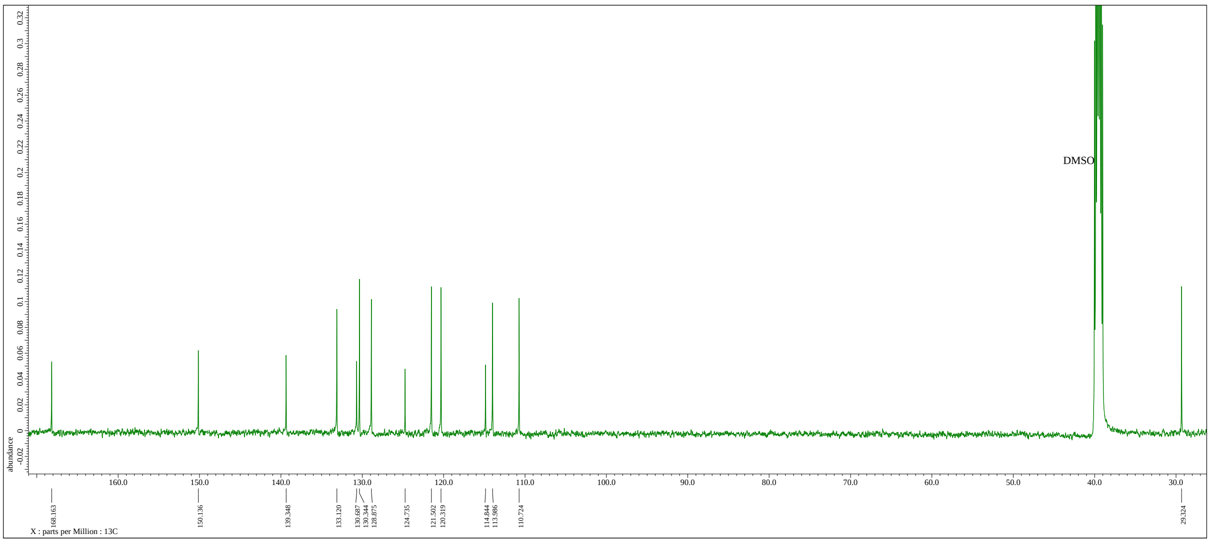

Cytotoxicity testing

Figure S3 | MTT Cytotoxicity Assay. A 3-[4,5-dimethylthiazole-2-yl]-2,5-diphenyltetrazolium bromide (MTT) assay was performed to measure the cytotoxicity of all compounds. The bars show the % viability of NTCP-HEK293 cells after incubation (15 min) with the indicated compound (100 µM). Shown are triplicates with standard deviation for each compound. DMSO 1% served as negative control (black color), cisplatin as positive control (red color).

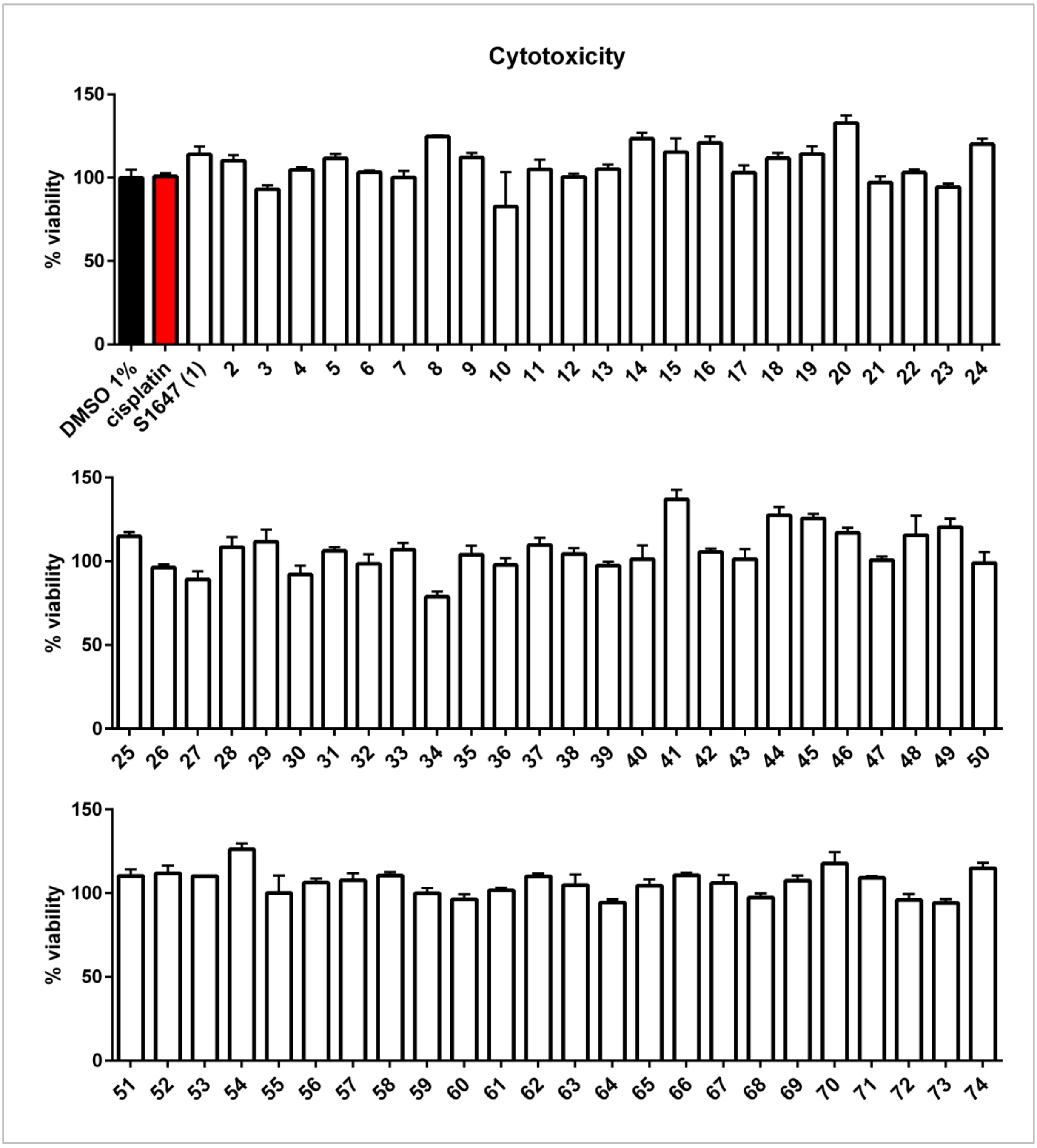

Supplement: Supplementary file 2 — jm4c01743_si_002.pdf [file jm4c01743_si_002.pdf]
